# How interfaces limit nanoscale stress concentrations and prevent catastrophic failure in single-asperity contacts

**DOI:** 10.1038/s43246-026-01239-1

**Published:** 2026-06-30

**Authors:** Michael Meindlhumer, Juraj Todt, Markus Alfreider, Asma Aicha Medjahed, Jakob Grau, Michal Zitek, Antje Dollmann, Fatih Uzun, Rostislav Daniel, Doris Steinmüller-Nethl, Hadwig Sternschulte, Manfred Burghammer, Petr Zeman, Verena Maier-Kiener, Alexander M. Korsunsky, Jozef Keckes

**Affiliations:** 1 Department of Materials Science, Montanuniversität Leoben, Leoben, Austria; 2 ESRF – The European Synchrotron, Grenoble, France; 3 Fakultät für Geistes- und Naturwissenschaften, Technische Hochschule Augsburg, Augsburg, Germany; 4 Department of Physics and NTIS – European Centre of Excellence, University of West Bohemia in Pilsen, Pilsen, Czech Republic; 5 Karlsruhe Institute of Technology (KIT), Institute for Applied Materials (IAM), Karlsruhe, Germany; 6 MBLEM - University of Oxford, Department of Engineering Science, Oxford, UK; 7 CarbonCompetence GmbH, Wattens, Austria; 8 Present Address: Center for Digital Engineering, Moscow, Russian Federation

**Keywords:** Mechanical properties, Characterization and analytical techniques, Materials science

## Abstract

Contact between materials occurs through microscale asperities, where stress evolves under highly localized, multiaxial deformation. Despite the significant importance to numerous scientific fields, to date, direct experimental quantification of these stress fields across the materials in contact has remained elusive. Here, we combine in situ scanning synchrotron X-ray nanodiffraction with 80 nm spatial resolution and a custom indentation setup to resolve nanoscale stress fields developing at the contact between a diamond indenter and both single-layer ZrN and multilayered ZrN–ZrCu thin films. We introduce a nanomechanical probe comprising a single-crystalline diamond wedge coated with nanocrystalline diamond to simultaneously probe stress accumulation within the indenter. X-ray scattering and scanning electron microscopy, micromechanical testing, analytical and elastic-plastic finite element modeling corroborate the nanoscale stress maps obtained for both sides of the tip-surface contact region. The individual interfaces along the projected load paths diffuse stresses in both the tip and the multilayer, thereby controlling the overall mechanical response at the asperity. Consequently, the stored elastic energy is reduced by 30% in the multi-layered film in comparison with the monolithic ZrN. Finally, the measured nanoscale stress distributions elucidate the fundamental mechanisms by which artificial multi-layered materials effectively dissipate energy and prevent catastrophic failure.

## Abstract (web summary)

Direct experimental quantification of stress fields during the contact of surface asperities remains elusive. Here in situ scanning synchrotron X-ray nanodiffraction with 80 nm spatial resolution and a custom indentation setup resolve nanoscale stress fields between a diamond indenter and single-layer ZrN and multilayered ZrN–ZrCu thin films.

## Introduction

Mechanical contact between dissimilar materials is a fundamental phenomenon with wide-ranging implications in both natural and engineering systems. Such contact rarely occurs between two perfectly oriented flat materials, but mostly takes place along single or multiple asperities, inducing highly localized, large multiaxial deformations on the materials in contact^1–4^. This observation fuels the field of contact mechanics, which is critical across many areas of materials science, including but not limited to bearings^5,6^, electrical contacts (i.e., in solid-state batteries^7,8^), sensors^9,10^, tire/road contacts^11,12^, and biomechanical systems^13,14^. Maintaining structural and functional reliability under these conditions remains a central challenge in modern materials science^15–18^.

Hard protective thin films are particularly affected, as they must operate in harsh and mechanically demanding environments, where both structural and functional reliability are essential^19^. The loading conditions may include elevated temperatures, corrosive environments, and complex multi-axial mechanical stress concentrations over sub-micrometer-scale distances. Consequently, improving resistance to deformation under single-asperity contact has motivated numerous optimization strategies to enhance mechanical properties of nanoceramic protective thin films.

In the case of nanoceramic monolithic transition nitride thin films, these strategies include alloying^20^, precipitation hardening^21–23^, and vacancy or interstitial engineering^24–27^ to strengthen columnar grain cohesion. However, due to the ceramic nature of protective (nitride) thin films, improving fracture resistance is fundamentally limited by the intrinsic properties of nanoceramic materials, which are inherently brittle under tensile loading, with ductility achievable only under compressive stress^25,28,29^. Therefore, modern thin films feature multilayered architectures and carefully controlled microstructures to enhance fracture resistance. Examples include tilted grain boundaries relative to the projected (expected) load path^30,31^, multilayers composed of alternating stiff and compliant phases across the film’s cross-section^32^, superlattice structures^20,26,33^, or various combinations of these approaches, complementing the toughening strategies for monolithic thin films.

To quantify the impact of the thin film design strategies outlined above, the state of the art methods for determining the average micro-mechanical properties of advanced thin films include analysis by nanoindentation, micropillar compression^25,28,29^ and in situ microcantilever bending tests in the SEM^17,21,22,34^. For the latter method, Young’s modulus, fracture stress and fracture toughness, respectively, can be extracted from the experiments. Nevertheless, the vast majority of protective nanoceramic thin films still exhibit brittle, linear-elastic failure upon crack initiation under tensile loading due to their ceramic nature^17,21,22,34^ (with only a few notable exceptions^22,35^). There is, however, a fundamental limitation in the current testing regime for nanoceramic protective thin films-mechanical characterization typically focuses on averaged properties measured usually under tensile loading. In reality, protective nanoceramic thin films are subjected to complex, multi-axial contact stresses during service^19,36^, where the transmission of mechanical forces between facing parts is highly localized, resulting in steep cross-sectional and lateral stress gradients. Some of these challenges can be partially addressed by nanoindentation^37,38^, which is commonly used to screen thin films for desirable mechanical properties. Nonetheless, microscopy-based methods remain limited to post mortem analysis and lack the capability to reveal how failure progresses locally within the material during loading. An approach to address this contact problem in more detail would be photoelasticity, which yields fringe patterns in dielectric materials under load that are proportional to the stress present in the material^39–41^. However, the spatial resolution of photoelasticity is limited to the order of 100 µm, and the stresses must be inferred through finite element (FE) modelling. While this remains the most widely used method for investigating contact mechanics^3,42–44^, it is clearly far from providing the resolution required for modern materials applications.

On the other hand, we have recently shown that, by coupling in situ nanoindentation with cross-sectional X-ray nanodiffraction, it is possible to reveal the nanomechanical response of protective thin films, highlighting the effect of specific residual stress gradients within single-material (monolithic) thin films^45,46^, as well as in multi-layered, multi-material thin films^47–49^. To fully resolve the nanomechanical contact between the imprinting indenter and the protective nanoceramic film, mechanical and structural processes on both sides of the contact—within the indenter and the film—must be analyzed simultaneously. Due to the limitation of the X-ray nanodiffraction approach to polycrystalline materials, the complementary response of the single-crystalline (sc) diamond indenter tip in these experiments has remained inaccessible^45–49^. Therefore, a dual-sided experimental analysis of the single-asperity contact has not been possible up to now, leaving a crucial gap in the existing knowledge. Nevertheless, only a dual-sided approach can uncover the nanoscale multi-axial stress gradients generated during single-asperity contact, which have remained largely elusive to materials scientists until now.

**Fig. 1:**
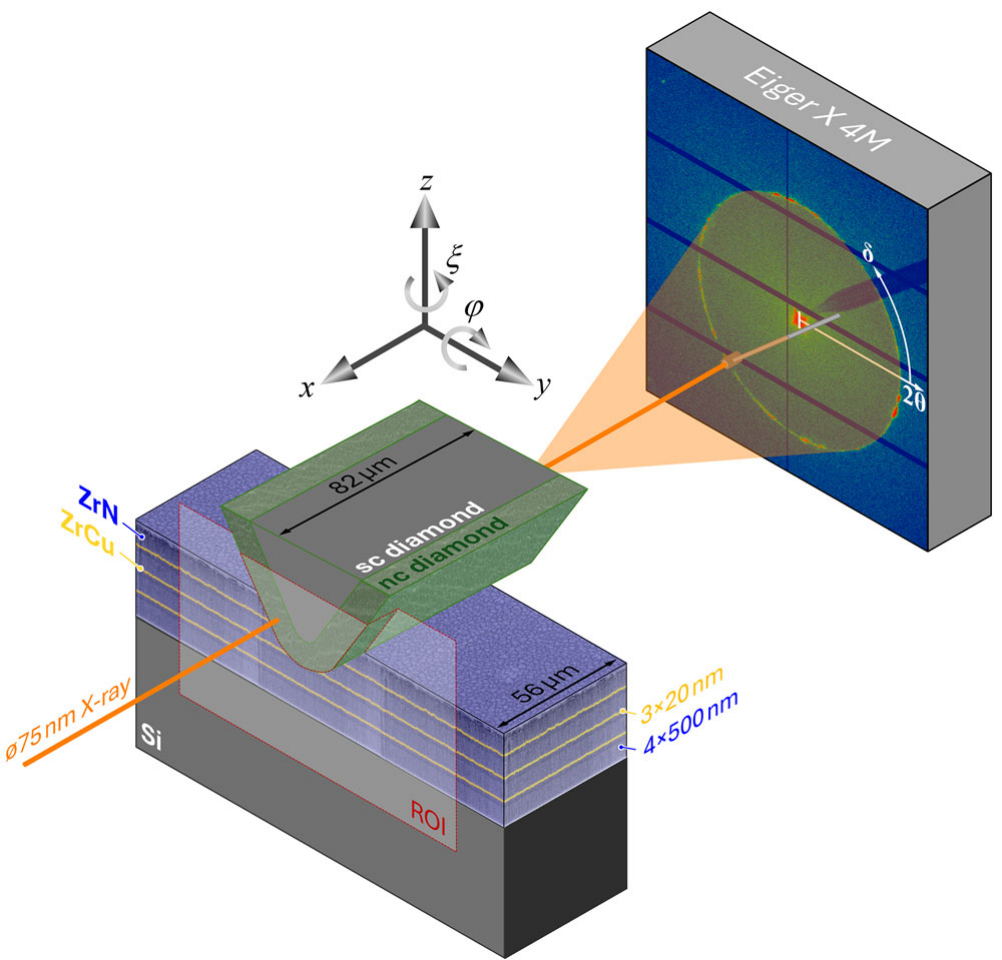
Schematic of the in situ CSnanoXRD experiment. The novel nanomechanical probe consists of a single-crystal (sc) diamond indenter tip, which was coated with nanocrystalline (nc) diamond by chemical vapour deposition. The probe is depicted in contact with the ML ZrN-ZrCu thin film at a constant load, while the region of interest (ROI) of *y* = 20 µm and *z* = 12 µm is scanned with an X-ray beam of 75 nm in diameter. A step size of 80 nm results into 37500 2D diffractograms analyzed per load step.

In this study, we adopted a two-fold approach to overcome this limitation and advance the state of the art using synchrotron cross-sectional X-ray nanodiffraction (CSnanoXRD). First, a diamond wedge indenter tip was coated with nanocrystalline diamond to create a unique nanomechanical diamond probe (NDP) (Fig. 1). Second, in situ micromechanical analysis, combining nanoindentation with X-ray nanodiffraction, was carried out on a multi-layered (ML) ZrN thin film with amorphous ZrCu interlayers and a single-layer (SL) ZrN thin film in contact with the NDP. This novel approach enables, for the first time, the quantification of evolving multi-axial stress distributions in both the indenter tip and the indented material with unprecedented spatial resolution. Thus, a dual-sided stress analysis of the single-asperity contact is enabled, in addition to the evaluation of morphological and microstructural changes across the contact area. The collected data provide a quantitative mechanistic explanation of how tough ZrCu interlayers in multilayered ZrN/ZrCu architectures mitigate high stress concentrations and fracture. The experimental synchrotron data are complemented by conventional in situ micromechanical testing, analytical considerations, and finite element simulations, which together confirm the observed deformation and failure mechanisms. Additionally, correlative electron microscopy is used to trace the post-failure crack paths. Collectively, these integrated experimental and computational approaches offer new insights into, and validate, the fundamental mechanical interactions governing single-asperity contacts, while highlighting the critical role of interface-engineered protective thin films.

## Results

### Laboratory micromechanical and electron microscopy analyses

**Fig. 2:**
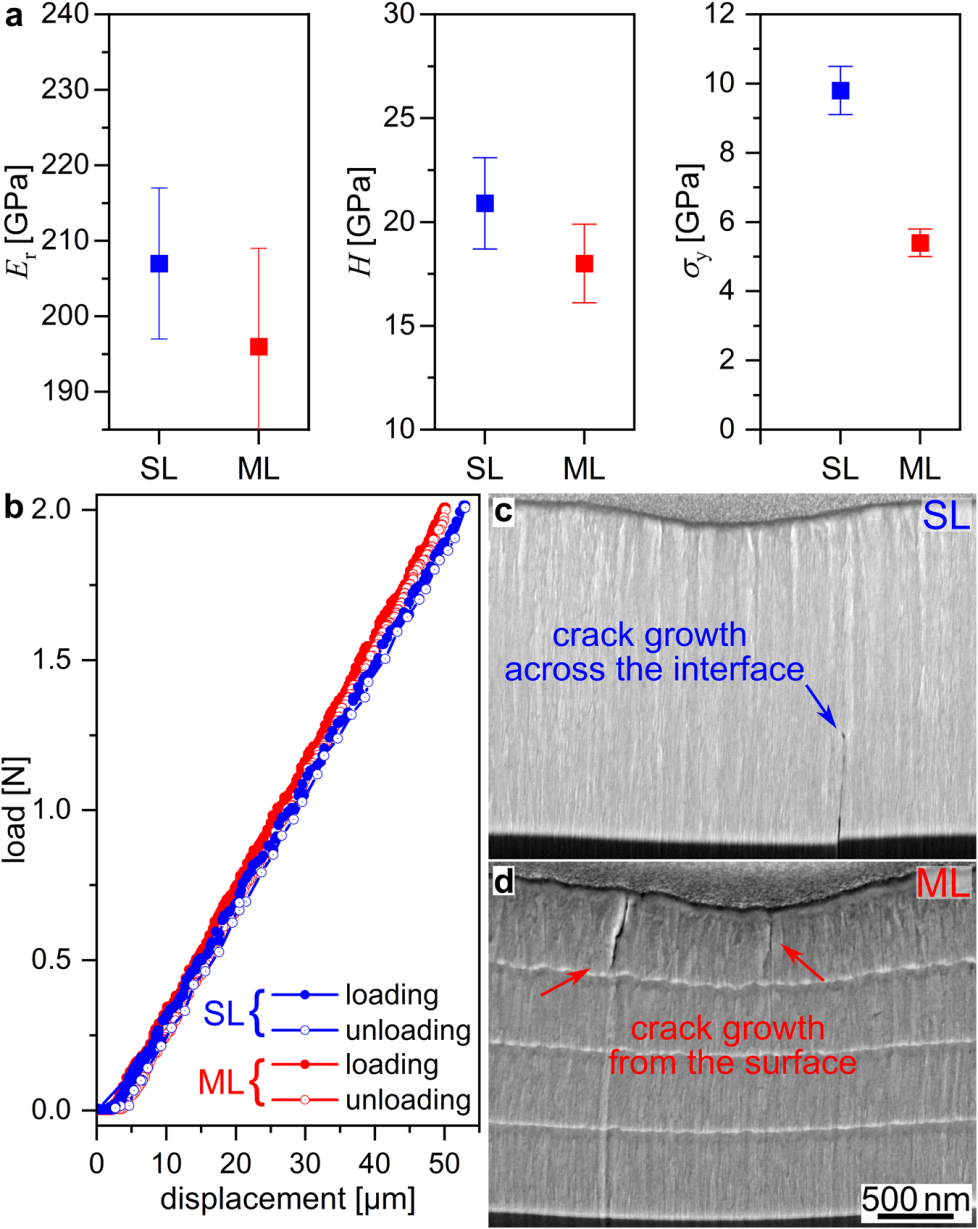
Results from micromechanical tests obtained from the monolithic (SL) and multilayered (ML) films. In (**a**), micromechanical properties of the films are presented, from left to right, the indentation modulus *E*_r_, the nanoindentation hardness *H*, as well as the composite yield stress *σ*_y_ obtained by spherical nanoindentation. The load-displacement curves recorded from the in situ indentation experiments coupled with X-ray nanodiffraction (*cf*. Fig. 1) are presented in (**b**). In (**c**, **d**) the FIB-polished post mortem cross-sections from the center of the imprint are shown for SL and ML films, respectively. The scale bar in (**d**) is applicable to (**c**, **d**).

Results from laboratory micromechanical testing performed on the ZrN single-layer (further denoted as SL) and ZrN-ZrCu multi-layered (further denoted as ML) thin films are presented in Fig. 2a, showing the reduced modulus *E*_r_ and hardness *H* obtained from nanoindentation measurements using a Vickers indenter tip, as well as the composite yield stress *σ*_y_ that was evaluated by spherical nanoindentation (Fig. 2a). Additionally, the Young’s modulus *E*, fracture stress *σ*_F_, and fracture toughness *K*_IC_ were measured via microcantilever bending and are presented in Suppl. Note 2. The micromechanical results indicate that both films exhibit nearly identical mechanical properties, as a consequence of the averaging effect inherent to Vickers indentation or predominantly tensile loading conditions (Suppl. Note 2, Fig. S3). On the other hand, spherical nanoindentation, which is intrinsically closer to the single-asperity contact, reveals highly different composite yield stresses (Fig. 2a). This is a first hint that the ML and SL films have significantly different mechanical response to realistic contact mechanical loading conditions. Another minor difference lies in the elastic (bending) moduli of the SL and ML films, which can be explained by their different texture evolution during deposition^50^. The overall similarity of the micromechanical properties is further supported by analysis of the pristine microstructures (Fig. S1) and the fracture surfaces of the tested cantilevers (Fig. S4). First, a certain degree of porosity is detected in both the SL and ML films (Fig. S1, Suppl. Note 1). Second, any sign of plasticity is absent in the fractures surfaces obtained from the microcantilever experiments on the SL and ML thin films (Fig. 2c, d, Fig. S4). Additionally, nanoindentation testing was carried out on the nc diamond deposited on sc Si to assess the hardness of the NDP, which was evaluated as 61.6 ± 6.1 GPa, while the Young’s modulus of 460 ± 53 GPa of the same thin film was already measured in a prior study^51^.

Load-displacement data obtained from the in situ indentation experiments coupled with X-ray nanodiffraction using the nanomechanical diamond probe (further denoted as NDP, Fig. 1) on the SL and ML films are presented in Fig. 2b. As evident from both load-displacement curves, the overall deformation response is quite similar, with the load-displacement curve obtained from the ML film being slightly stiffer compared to the one recorded during deformation of the SL film. However, detailed post mortem cross-sectional SEM analyses of the SL and ML films presented in Fig. 2c, d highlight the differences in thin film failure modes. While the cracking initiates from the interface between protective thin film and substrate when loading the SL film (Fig. 2c), the crack formation and propagation in case of the ML film is observed exclusively in the topmost ZrN layer.

While individual fracture features, like those in Fig. 2c, d, are inherently statistical, post mortem SEM analysis of the indents produced by complementary spherical nanoindentation (Suppl. Note 2, Fig. S5) revealed a comparable overall failure behavior for both the SL and ML films. This observation confirms that the results obtained from the in situ X-ray nanodiffraction experiments are not incidental but reflect a physically meaningful and reproducible mechanical response. Consequently, although the benefits of multi-layered films reported throughout the thin film literature^20–27,30,31^ are completely hidden in the micromechanical data presented in Fig. 2a, the distinct differences in failure characteristics between the SL and the ML films, as seen by microscopy techniques (Fig. 2c, d, Suppl. Note 1, Fig. S2) in this load case, are striking.

### In situ synchrotron X-ray nanodiffraction analysis of microstructural changes and stress distributions in the NDP, SL, and ML thin films

Microstructural changes within the loaded SL and ML films at the peak load of 2.0 N, as revealed by X-ray nanodiffraction, are presented in Fig. 3, while the full details of additional loading steps and the progression of deformation accumulation are provided in Supplementary Notes 3 and 4 for the SL (Figs. S6–S11) and ML (Figs. S12–S17) thin films, respectively.

**Fig. 3:**
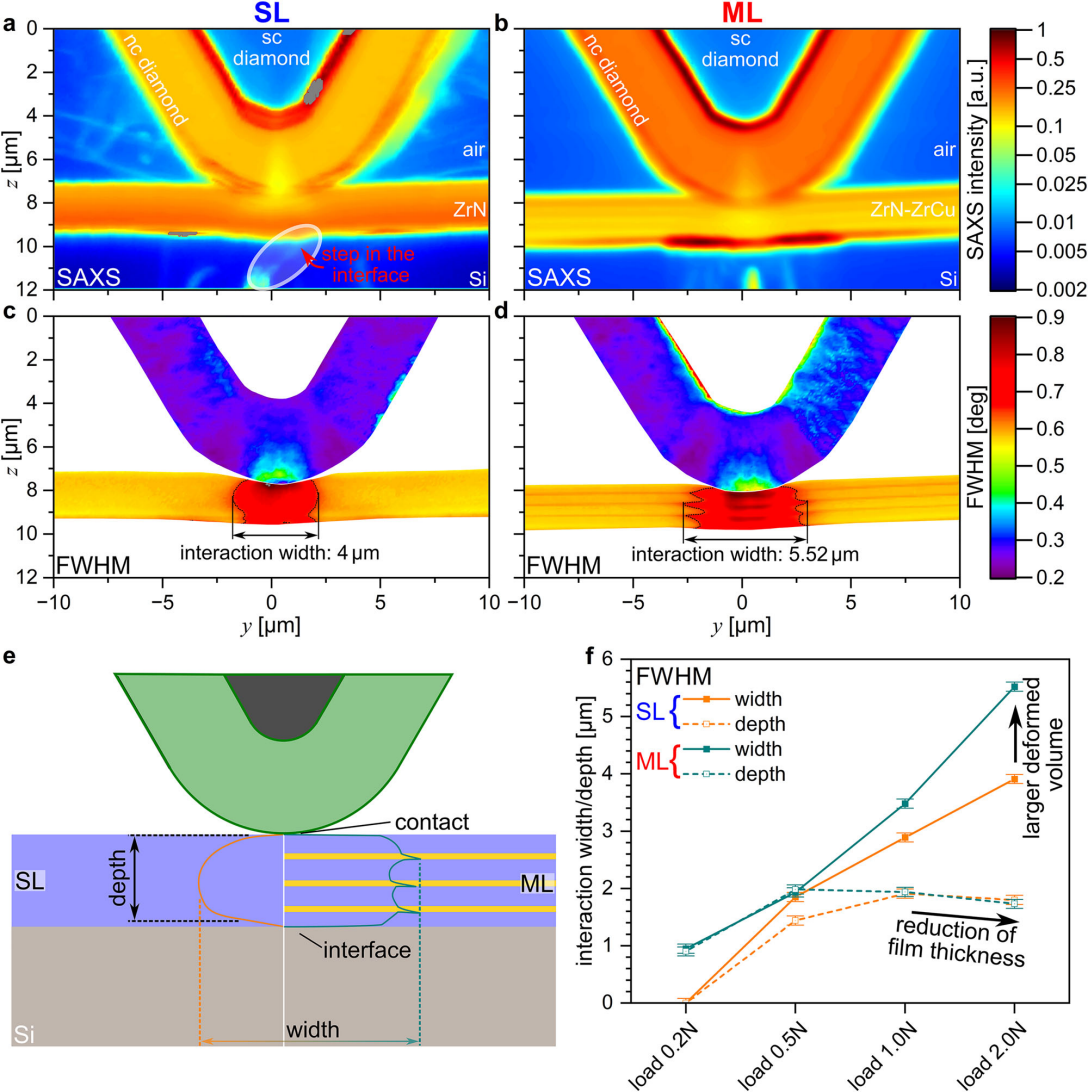
Microstructural changes in the SL and ML as well as in the nanocrystalline tip probe at a load of 2.0 N. In (**a**) and (**b**) the SAXSM micrographs obtained from the SL and ML are shown. The highlighted region in (**a**) represents a step in the Si/ZrN interface. In (**c**, **d**) micrographs depicting the azimuthally averaged FWHM are presented, respectively. The dotted black line in (**c**, **d**) represents a FWHM of ~0.65 deg. The schematic experimental setup (**e**) is shown alongside the progressive evolution of the FWHM interaction *width* and *depth* during incremental indenter loading presented in (**f**).

Small-angle X-ray scattering microscopy (SAXSM^52^) in Fig. 3a, b highlights the variation of electron density within the X-ray gauge volume. This allows us to qualitatively evaluate the occurrence of pores, interfaces, cracks, and inclusions, as well as their changes within the gauge volume during in situ experiments. In the SAXS micrographs of the SL and the ML films presented in Fig. 3a, b, a narrow region of lower scattered intensity can be recognized in the central part of the diamond indenter for both samples, respectively. This lower intensity represents the growth of micron-sized diamond crystals during deposition and is directly related the CVD diamond growth conditions (*cf*. Methods, Suppl. Note 10).

**Fig. 4:**
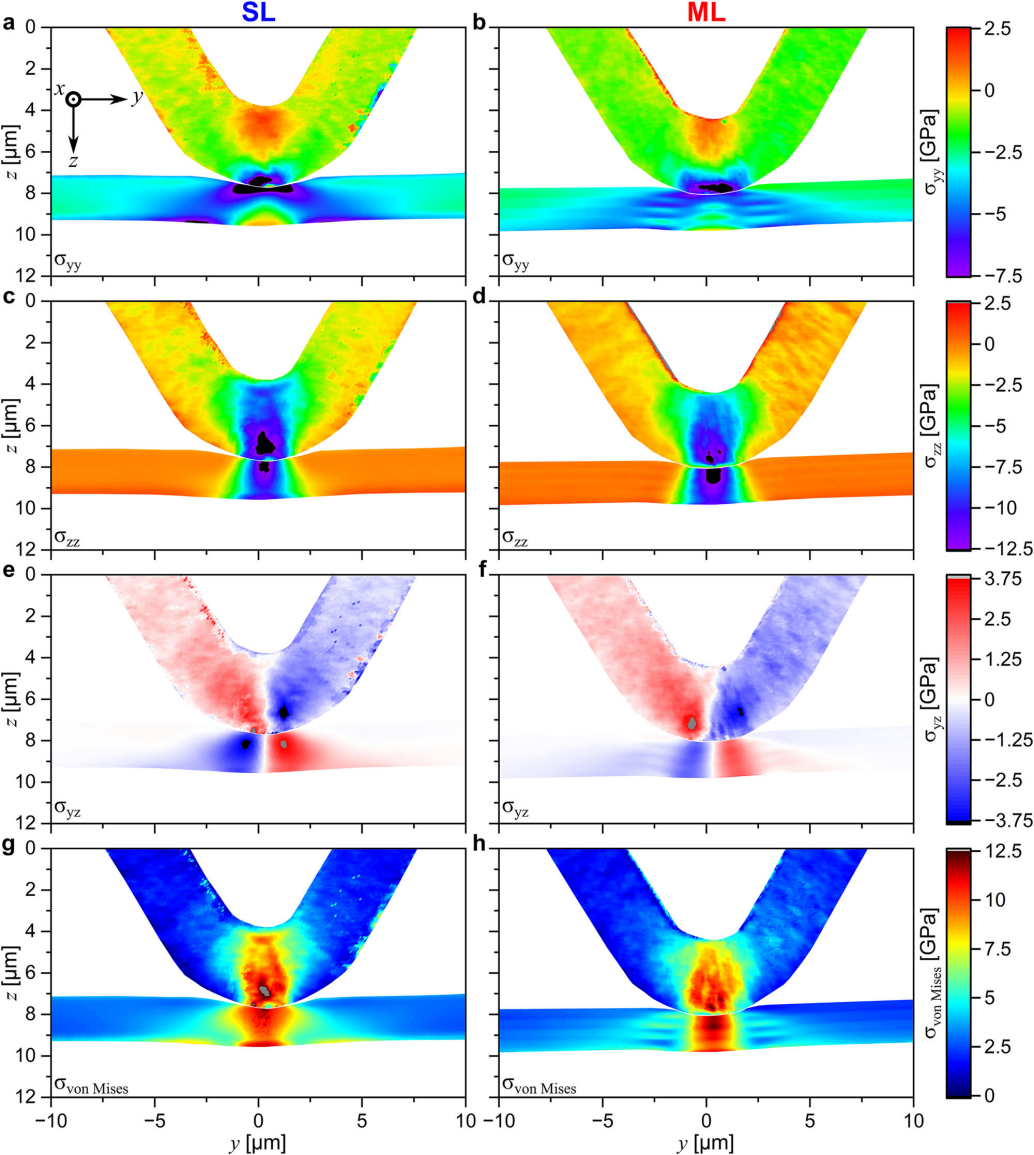
CSnanoXRD data from the NDP, SL and ML films obtained during in situ indentation experiments coupled with X-ray nanodiffraction at a load of 2.0 N. The *σ*_yy_, *σ*_zz_ and *σ*_yz_ components of the stress tensor are presented in (**a**–**f**) for the ML and SL in contact with the NDP, respectively. Additionally, in (**g**, **h**) the *σ*_von Mises_ calculated from the stress components are displayed for the experiments involving the SL and ML, respectively.

In Fig. 3a, a reduction of the SAXS intensity can be seen in the SL within a ~ 500 nm high region located beneath the indenter tip, which is interpreted as a result of densification of the porous thin film due to the high compressive load (*cf*. Fig. 4). Regions of raised SAXS intensity include a step at the interface between ZrN and Si and an adjacent line of increased intensity under an angle of 54 deg with respect to the ZrN/Si interface, terminating in a vertical zone of highly elevated SAXS intensity (Fig. 3a). Both these regions are indicative of cracks, which were also confirmed by post-experiment ex situ SEM analysis (Suppl. Note 1).

On the contrary, the ML thin film shows a highly extended zone of slightly reduced SAXS intensity beneath the indenter tip (Fig. 3b), spanning over the entire film thickness, which indicates some densification due to the compressive stress state (Fig. 4). However, the markedly smaller intensity change indicates that this densification occurs to a significantly lesser extent compared to the SL film. Furthermore, a zone of increased intensity lines is also found in the Si substrate, again indicating fracture of the Si, but with the major difference that this zone remains disconnected to the ML thin film (Fig. 3b), which is also corroborated by post-experiment ex situ SEM (Suppl. Note 1).

Full width at half maximum (FWHM) microscopy^53^ gives insight into intracrystalline defects, including varying dislocation and vacancy distributions that can be interpreted as strains of 2^nd^ and 3^rd^ order, as well as gradients of 1^st^ order strains within the X-ray gauge volume. Starting with the diamond-coated indenter tip, which undergoes only elastic deformation during the indentation of the SL and ML thin films (see Suppl. Notes 3 and 4), the changes in the averaged FWHM of the diamond 111 Debye-Scherrer (DS) ring in the NDP during loading can be interpreted exclusively as strong gradients of 1^st^ order stresses present within the X-ray gauge volume. In both cases, stresses change markedly over short *z*-distances: between −15 and 2 GPa over the course of only 3.8 µm, as detailed below for both the SL and ML thin films, while the lateral stress variations are even more pronounced. Consequently, in nanocrystalline diamond, the FWHM increase serves almost directly as a measure of the stress gradient and is observed only near the contact area with SL and ML thin films (Fig. 3c, d, respectively).

For the SL and ML thin films, however, the increase of the averaged FWHM of ZrN 111 DS ring reflects not only these stress gradients. A FWHM value of > 0.65 deg was chosen to highlight the zone of maximum deformation with a contour line in Fig. 3c, d. This specific threshold was selected as it exceeds the average FWHM in the SL and ML films before deformation (Suppl. Notes 3 and 4) by three times the standard deviation, thereby exceeding any statistical variation of the FWHM. In case of the SL thin film, this zone has a width of ~4 ± 0.12 µm. Additionally, the area of reduced SAXS intensity (Fig. 3a) discussed above coincides with an FWHM increase well above 0.65 deg, indicating possible grain boundary sliding and fracture of the individual ZrN columns, in addition to densification of the film, which is in agreement with literature^53^.

In direct comparison of Fig. 3c, d, it can be deduced that the width of the region where FWHM > 0.65 deg is significantly larger in case of the ML thin film, measuring 5.52 ± 0.12 µm. Despite the fact that the overall FWHM of the undeformed material is quite similar for the SL and ML films (compare Fig. 3c, d), the zones where the FWHM well exceeds 0.65 deg are not limited to the vicinity of contact area in case of the ML film (Fig. 3d). Here, these zones are present in the upper parts of all individual layers and indicate the presence of plastic deformation throughout the ML film thickness.

For a better understanding of the similarities and differences between the mechanical and structural behavior of the SL and the ML thin films, a direct comparison of several distinct features identified in the mappings is presented in Fig. 3f as indicated in the schematic in Fig. 3e. The widths (in *y*-direction) and depths (in *z*-direction) of the regions of FWHM > 0.65 deg (equivalent to a size of coherently diffraction domains of 8.5 nm) are compared for the SL and ML film as a qualitative measure for the deformed volume. While this interaction depth is limited by the film thickness and its slight decrease during later loading stages (Fig. 3f), the width of the interaction is increasing steadily during loading. As one can see from Fig. 3c and Fig. 3d for the SL and ML thin films, respectively, as well as from the direct comparison in Fig. 3f, the interaction width is constantly higher for the ML film, indicating a greater spread of the non-reversible deformation, which is manifested as an FWHM increase.

Evolving stress components observed during the in situ experiments are presented in Fig. 4 for the SL and ML films at the peak load of 2.0 N, while the stress distributions recorded at other loads are shown for the SL (Figs. S6–S11) and ML (Figs. S12–S17) thin films in Suppl. Notes 3 and 4, respectively.

In the case of the SL sample, the horizontal stress component *σ*_*yy*_ shows a maximum stress level of −8.7 ± 1.2 and −9.3 ± 0.3 GPa in diamond and ZrN directly at the contact, respectively (Fig. 4a). The compressive stress concentrations are spatially constricted both vertically and horizontally, with significant stress levels limited to the region between *y* = −2.5 and 2.5 µm. In addition to the high compressive stress, tensile stresses were evaluated close to the nc diamond/sc diamond interface (*z* = 4 µm) as well as the ZrN/Si interface (*z* = 9.4 µm). While the stresses are symmetrical at the contact (in compression), the tensile *σ*_*yy*_ reaches up to 1.9 ± 0.2 GPa in nc diamond in comparison to 0.2 ± 0.2 GPa evaluated close to the ZrN/Si interface. The comparably lower tensile stress at the ZrN/Si interface is likely a result of crack formation, leading to local removal of constraint as is also observed in the ex situ SEM analysis (Fig. 2). In contrast, the tensile stress in diamond was not high enough to induce fracture, as indicated by a fracture stress of 7.8 ± 1.0 GPa recorded from micromechanical tests^51^.

The experimental *σ*_*yy*_ data for the ML film in contact with the NDP are presented in Fig. 4b. The horizontal stress component *σ*_*yy*_ also exhibits a high stress concentration at the contact, reaching up to −8.2 ± 0.7 GPa and −8.2 ± 0.5 GPa in diamond and ZrN, respectively (Fig. 4b). The stress distribution in the NDP in contact with the ML (Fig. 4b) is similar to that obtained for the indenter in contact with the SL (Fig. 4a), except that the absolute stress magnitudes are slightly lower. However, the response of the ML film is completely different (Fig. 4b): The absolute stress magnitudes at the contact are significantly lower, while the compressively stressed area is considerably broader in the lateral direction (compare Fig. 4a, b). Additionally, the maximum *σ*_*yy*_ reaches only −1.0 ± 0.4 GPa (Fig. 4b), meaning it remains well within the compressive regime even at the substrate interface.

The vertical stress component *σ*_*zz*_ within the SL and the NDP is presented in Fig. 4c and shows an even higher compressive stress concentration at the contact reaching up to −13.8 ± 1.2 GPa in diamond and −13.4 ± 0.4 GPa in ZrN (Fig. 4c). In the case of the ML film, *σ*_*zz*_ shows a compressive stress concentration at the contact reaching up to −13.4 ± 1.2 GPa in diamond and and −12.4 ± 0.6 GPa in ZrN (Fig. 4d). Therefore, within the accuracy of the experiments, the fundamental requirement of force equilibrium at the contact is fulfilled^54^.

In general, while the profiles of the *σ*_*zz*_ distributions are highly similar for both experiments (compare Figs. 4c and 4d), the absolute stress level of the ML thin film is slightly lower (Fig. 4d). Furthermore, in both experiments, the compressive stress zone is highly localized, ranging between *y* = −2.0 and 2.0 µm, while its width increases with the distance from the contact (Fig. 4c). In comparison to the *σ*_*yy*_ component presented in Fig. 4a, the gradient of *σ*_*zz*_ is less pronounced (Fig. 4c). This contrasts with linear-elastic Hertz contact theory^1,55^ and indicates a significant amount of inelastic deformation in both films.

The shear stresses *σ*_yz_ within the contact area between the NDP and the SL and ML films are presented in Fig. 4e, f, respectively. An antisymmetric shear profile across the contact center is observed in both experiments. While the maximum stresses *σ*_yz_ reach ± 4 GPa in both the NDP-SL (Fig. 4e) and NDP-ML contacts (Fig. 4f), the antisymmetric shear profile in the ML film exhibits stresses of just ± 2.5 GPa. In agreement with the inherent symmetry of the single-asperity contact, the shear stress magnitudes are point symmetric across the contact center^56^. Moreover, the shear stress distributions in the ML film show a periodic reduction close to *σ*_yz_ = 0 at the cross-sectional positions of the amorphous ZrCu interlayers (Fig. 4f). This *σ*_yz_ stress level suggests a significant influence of the metallic glass on the deformation response of the ML film during the in situ indentation experiment coupled with X-ray nanodiffraction.

Finally, the von Mises stress *σ*_von Mises_ distribution representing the stored distortion energy in the NDP and the SL film is presented in Fig. 4g, where maximum *σ*_von Mises_ of 12.5 and 13.6 GPa are found for ZrN and diamond close to the contact, respectively. Additionally, a secondary maximum is found in ZrN close to the ZrN/Si interface, where the *σ*_von Mises_ reaches 11.7 GPa (Fig. 4g), indicating build-up of stored distortion energy close to the ZrN/Si interface. The *σ*_von Mises_ distribution in the NDP in contact with the ML film is presented in Fig. 4h, where maximum *σ*_von Mises_ of 12.2 and 12.1 GPa are found for ZrN and diamond close to the contact, respectively. In addition to the von Mises stress accumulation at the contact, lines of increased *σ*_von Mises_ at the positions of the ZrCu interlayers indicate build-up of distortion energy close to the ZrN/ZrCu interfaces (Fig. 4h).

### Analytical and numerical recreation of the experimentally obtained stresses in the NDP, SL and ML films

**Fig. 5:**
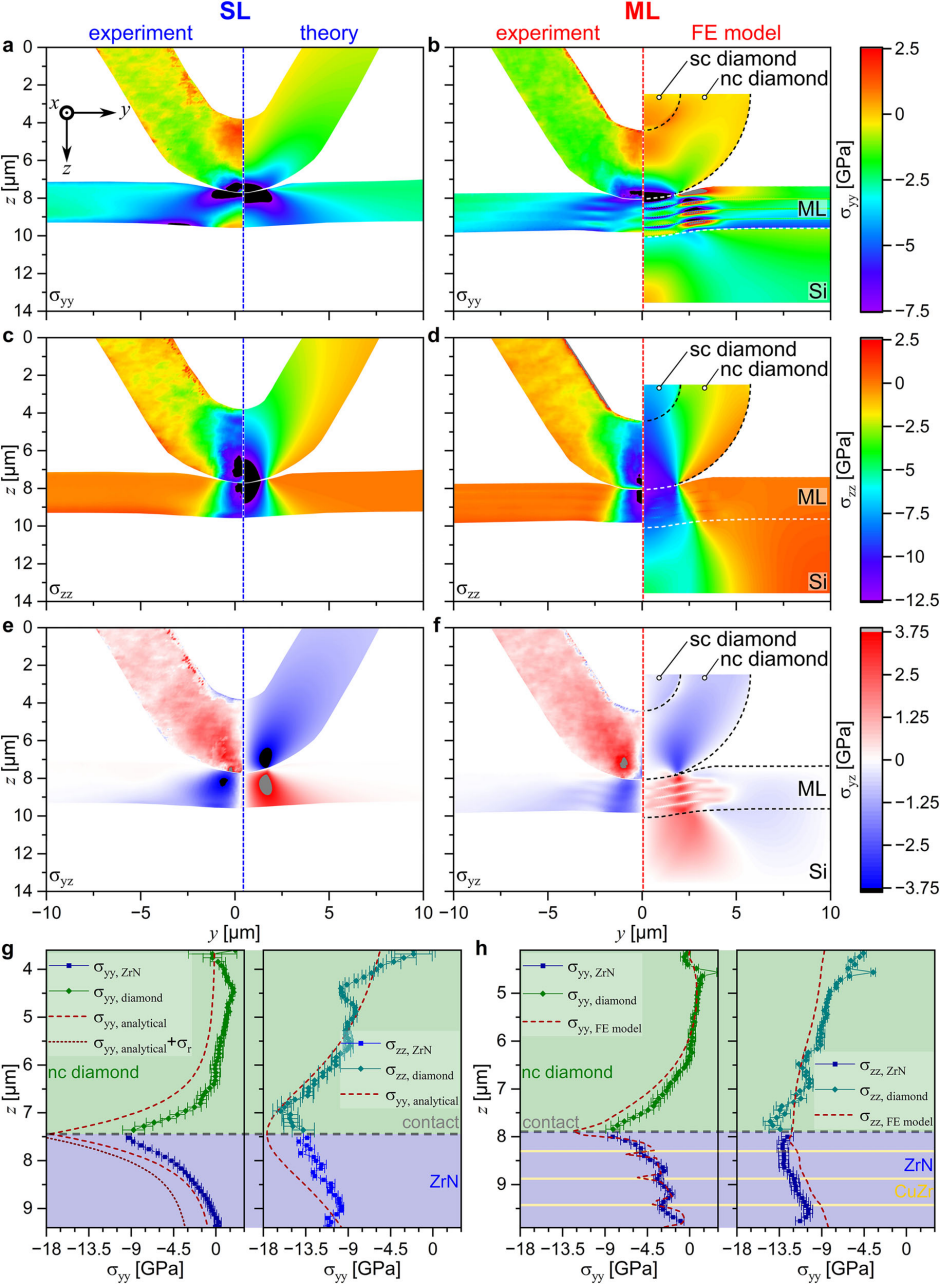
Comparison of the experimental data with analytically-derived and FE-modelled stress distributions. The *σ*_*yy*_*, σ*_*zz*_ and *σ*_*yz*_ stress distributions derived by the linear-elastic analytical solution given by M’Ewen^55^ are shown in comparison with the experimental data from the NDP in contact with the SL film in (**a**, **c**, **e**), respectively, while the FE-modelled *σ*_yy_,* σ*_zz_ and *σ*_yz_ stress distributions are presented against the experimental data of the ML in (**b**, **d**, **f**), respectively. Additionally, virtual sections along the symmetry plane were taken from the SL and ML in contact with the NDP and compared with the analytical and numerical data in (**g**, **h**), respectively. In (**g**), ${\sigma }_{{{\rm{r}}},{{\rm{ZrN}}}}$ represents the average residual stress present in the SL before the experiment (Suppl. Note 3).

In this section, the experimental results obtained from the nanomechanical contact of the NDP with the SL and the ML thin films will be compared with theoretical predictions using the linear elastic contact solutions^1,55^ and a finite element (FE) model for the more complex multi-layered case. The full descriptions of the elastic contact solution (Fig. S19) are presented in Suppl. Note 6. Please note that the complete datasets of the FE model including a parametric study with varying friction coefficients is present in Suppl. Note 7. However, since no significant influence of interfacial friction was observed, the numerical data of the frictionless contact between the NDP and the ML film are presented and compared with the experimental data of the ML film Fig. 5b, d, f.

Using Eq. S13 (Suppl. Note 12), a contact pressure of 17.0 GPa can be calculated, which corresponds to the maximum stresses in *y*- and *z*-direction directly at the contact assuming linear elastic behavior. Further, a contact width of 2*a* = 2.66 µm can be calculated using Eq. S14. In comparison with the experimental data presented above (*cf*. Fig. 4), this simple model estimates the *σ*_*zz*_ stress component quite accurately but overestimates *σ*_*yy*_ by a factor of 2 (*cf*. Fig. 3).

Furthermore, using the analytical treatment by M’Ewen^55^ (Eqs. S15-S17, Suppl. Note 12), the 2D stress distributions were calculated based on the contact pressure and width, and are displayed against the experimental data in Fig. 5a, c, e. In order to ensure a better quantitative comparison, the average residual stress of −2.42 GPa evaluated from the ZrN SL thin films was added to the theoretically derived *σ*_*yy*_ stress distributions presented in Fig. 5a. To accommodate a more complex case of the ML thin film in contact with the NDP, FE modelling was performed using the eigenstrain reconstruction method (ERM^57,58^, *cf*. Methods). The resulting *σ*_*yy*_*, σ*_*zz*_ and *σ*_*yz*_ stress distributions at the peak load of 2.0 N are displayed against the experimental data of the ML thin film and the NDP, as well as the sc diamond indenter tip in Fig. 5b, d, f, respectively. Finally, virtual sections along the symmetry plane were taken from both experiments and compared with the analytical and numerical data in Fig. 5g, h, respectively.

Overall, the agreement in the outline of the stressed regions between the simplistic linear elastic considerations and the experimental data obtained from the SL thin film in contact with the NDP (Fig. 5a, c, e, g) is striking, despite the fact that plastic deformation, the presence of dissimilar interfaces, as well as fracture in the ZrN and the Si substrate are not considered by the analytical estimate.

Furthermore, the analytical data fit the experimental case studies very well in the NDP, as can be seen for the contact with the SL film in Fig. 5g. In particular, *σ*_*zz*_ is represented accurately, even in magnitude by the analytical solution (Fig. 5g), while a shift towards more tensile stress is observed in the experimental data for *σ*_*yy*_ (Fig. 5g).

The numerically recreated *σ*_*yy*_ stress component is presented in Fig. 5b. In addition to the stress distribution in the NDP, also the *σ*_*yy*_ magnitudes in the sc diamond can be investigated within the FE model (Fig. 5b). A strong gradient from compressive to tensile stress is seen in the center of the numerically recreated NDP, in perfect agreement with the experimental data. Additionally, slight tensile stresses are found in the sc diamond (Fig. 5b). Furthermore, two major contributions to the stress field in ZrN can be found. First, there is the obvious contribution from the indentation, resulting in high compressive stresses in the center of the imprint, which diminish with increasing depth and become tensile within the Si substrate at a depth of 4-5 µm. This stress contribution perfectly represents the experimental data (from Figs. 4 and 5b). Second, there is a bending-like stress contribution in each ZrN sublayer adjacent to the imprint, where compressive and tensile stresses alternate very narrowly. This second contribution is induced by the relatively weak ZrCu interlayers, which allow a lateral reorganization (in *y*-direction) of the ZrN. As a result, the compressive stress in the center of the indent can be spread out into the adjacent regions, and this effectively reduces the *σ*_*yy*_ stress gradient over depth (*z*-direction). Please note that the individual stress components may exceed the yield stress of ~1.8 GPa of ZrCu^50,59^, since the hydrostatic component of the stress tensor limits plastic flow. Concerning this second contribution to the *σ*_*yy*_ stress component, the model gives an additional insight into the experimental data, which do not show tensile stresses. A likely explanation for this discrepancy can be found in the experimental limitations, such as the finite X-ray gauge volume, the roughness of the individual interfaces or slight misalignment against the *φ* and *ξ* rotational axes (see Fig. 1, Methods), all of which prevent the resolution of these ultra short-range stress concentrations in the experimental data. This is not only true for the *σ*_yy_ stress component, but for also for *σ*_*zz*_ and *σ*_*yz*_. Additionally, at higher lateral distances to the center of the indent, at the ZrN/Si interface increased compressive stress magnitudes are found in the model, in agreement with the experimental data (Fig. 5b).

Regarding the modelled *σ*_*zz*_ stress component presented in Fig. 5d, a maximum compressive stress of ~−12.5 GPa is found directly in the center at the interface between the ZrN and the NDP, which matches remarkably well to the experimental data (Fig. 4, Fig. 5d). Also, the sc diamond is under compressive stress, with magnitudes exceeding −7.5 GPa throughout the investigated volume (Fig. 5d). Slight deviations between the experiment and the FE model can be attributed to the boundary conditions of the modelled bilayer indenter tip. Furthermore, the outline of the stress profile fits perfectly with the experiment. Tensile stresses along the* z*-direction are only found in Si at the edges of the modelled area, which can be attributed to with the boundary conditions of the FE model.

The *σ*_*yz*_ stress component is depicted in Fig. 5f, showing an antisymmetric behavior of the shear stresses, as expected from theory and seen in the experimental data (Fig. 5f). However, the shear stress gradients in the lateral direction observed in the experiment (Fig. 4f) are higher compared to the model (Fig. 5f), where high shear stress localization is found outside the contact area. Finally, virtual vertical sections of experimental and numerical stress data are shown in Fig. 5h. Here again, the numerical data show excellent agreement with the experimental case studies, particularly in the NDP region in contact with the ML film. (Fig. 5h). In particular, the transition from compressive to tensile stress observed for *σ*_*yy*_ is represented accurately, while slight shifts towards more compressive and tensile stresses are found for *σ*_*zz*_ in the NDP and ZrN at larger distances from the contact, respectively. These slight differences may be attributed to the boundary conditions and fracture in the Si substrate for the NDP and the ZrN, respectively. Overall, the experimental data are in excellent agreement with the theoretical predictions and modelled data, allowing us now to consider the fundamental differences between the nanomechanical responses of the SL and ML thin films in contact with the NDP.

## Discussion

To analyze the multi-axial stress responses within the SL and ML thin films in contact with the NDP, this discussion is organized into two principal components. First, we examine the implications of converting the indentation tip into a nanomechanical probe, a feat unattainable in previous experimental approaches using sc diamond indenter tips^45–49^. Second, we assess the enhanced mechanical resilience and stability of the ZrN–ZrCu multilayer relative to the ZrN monolithic thin film under experimental conditions that approximate realistic loading environments.

To interpret the collected multi-axial stress data evaluated from dual-side analysis of the single-asperity contact (Figs. 4–5), the hardness of the cylindrical contact^60^ is considered in analogy to the Meyer hardness^61^, which is nowadays accepted as a hardness criterion in indentation. This can be done by reversing the argument of Tabor and later works^62,63^, where the yield stress ${\sigma }_{{{\rm{y}}}}$ is proportional to the indentation hardness ${H}_{i}$ by a factor of 2.8–3. Therefore, the hardness of the cylindrical contact ${H}_{c}$ should be ≈ 15.8 – 16.8 GPa, based on the measured nanoindentation hardness value of the SL film (Fig. 2a). Applying the Meyer hardness directly to the cylindrical contact, the geometrically derived ${H}_{c}$ value is in significantly better agreement with the experimental data ($\left|{\sigma }_{{{\rm{zz}}}}\right|$, ${\sigma }_{{{\rm{von\; Mises}}}}$ ≈ 13 GPa), while ${H}_{c}$ is closer to the analytical elastic contact solution (Fig. 5), where a contact pressure of 17 GPa was calculated. A full description of these derivations is presented in Suppl. Note 12. Theses obvious differences highlight that the constraint factors, which connect hardness and yield strength are material- and geometry-dependent^62^. Of course, both the analytical solution for the cylindrical^55^ contact and the elasto-plastic FE model^60^, were formulated under the assumption of infinite half-spaces. In turn, these models do not capture the full complexity of the present system, which includes interfaces between sc diamond and nc diamond in the NDP, interfaces between the SL and ML thin films and the Si substrates, as well as the cracks within both samples discovered after the synchrotron experiment (Fig. 2). While the NDP may not be described as a classically sharp nanoindenter tip owing to its relatively large size, it possesses a radius comparable to that of a single-asperity on a well-polished surface^64^. Thus, the NDP provides valuable insight into the deformation behavior of both nc diamond and the SL and ML thin films under realistic contact loading scenarios.

In the following, the role of interfaces within the tip-SL and tip-ML systems in governing the nanoscale mechanical response will be discussed. While ${\sigma }_{{{\rm{zz}}}}$ distributions in the nc diamond are in excellent agreement with the elastic predictions^55^ as shown in Fig. 5c, the experimental ${{{\rm{\sigma }}}}_{{{\rm{yy}}}}$ stress component in the NDP is systematically shifted towards higher tensile stress levels (Fig. 5a, g). However, the ${\sigma }_{{{\rm{yy}}}}$ stress distributions of the FE model are perfectly representing the experimental data (Fig. 5h). Since the diamond underwent purely elastic deformation in this study (Suppl. Notes 3 and 4), these shifts cannot be ascribed to plastic deformation within the NDP itself, but therefore must rather arise solely from various interfaces within the mechanical system. A first, simple analysis accounts for the strain compatibility at the sc/nc diamond interface (*i.e*. ${\varepsilon }_{{{\rm{yy}}}}^{{{\rm{sc}}}}={\varepsilon }_{{{\rm{yy}}}}^{{{\rm{nc}}}}$), while the force across the interface has to be in equilibrium (*i.e*. ${\sigma }_{{{\rm{zz}}}}^{{{\rm{sc}}}}={\sigma }_{{{\rm{zz}}}}^{{{\rm{nc}}}}$). Adding the elastic constants of sc diamond^65^ (E^sc^=1120 GPa, ν^sc^ = 0.07) and nc diamond^51,66–68^ (E^nc^=460 GPa, ν^nc^ = 0.15) into generalized Hooke’s law^54^, a stress of ${\sigma }_{{{\rm{yy}}}}^{{{\rm{nc}}}}=\frac{{E}^{{{\rm{nc}}}}}{{E}^{{{\rm{sc}}}}}{\sigma }_{{{\rm{yy}}}}^{{{\rm{sc}}}}-{\sigma }_{{{\rm{zz}}}}\left(\frac{{\nu }^{{{\rm{sc}}}}\cdot {E}^{{{\rm{nc}}}}}{{E}^{{{\rm{sc}}}}}+{\nu }^{{{\rm{nc}}}}\right)$ is introduced at the interface of the nc diamond. Assuming now that the $\frac{{E}^{{{\rm{nc}}}}}{{E}^{{{\rm{sc}}}}}{\sigma }_{{{\rm{yy}}}}^{{{\rm{sc}}}}$ contribution is negligible and taking ${\sigma }_{{{\rm{zz}}}}$ ≈ −6 GPa at the interface (Figs. 4–5), we obtain ${\sigma }_{{{\rm{yy}}}}^{{{\rm{nc}}}}$ ≈ 1 GPa. Additionally, according to FE-models where a rigid flat was pressed into a coated sphere^42,69^, the interface between the coating and the substrate in the sphere is directly connected to the buildup of tensile *σ*_*yy*_. Empirically, the maximum tensile stress *σ*_*yy*_, was observed precisely at the sphere–coating interface beneath the contact points, while the ratio of the maximum compressive to tensile *σ*_*yy*_ magnitudes was determined to be 5:1. Coincidentally, this ratio, which is derived from a complex relationship between the radius-to-thickness ratio and the elastic moduli and yield strengths of both thin film and substrate, also applies to the maximum tensile stress in the nc diamond in contact with both the SL (Figs. 4a, 5g) and ML (Figs. 4b, 5h) films. To be more precise, the experimental data show that the maximum tensile stress does not occur directly at the nc/sc diamond interface, as in the model^42^ and in our simple considerations, but rather within the nc diamond approximately 500 nm away from the interface (Figs. 4a, b, 5g, h). Additionally, The FE model presented in Fig. 5 is in excellent agreement with the experimental data, solidifying our interpretation of the experimental stress distributions. Following another consideration, when two cylinders of dissimilar materials are in contact, the applied normal pressure leads to an additional tangential traction acting along the contact surfaces^56^, which is limited by the normal pressure times the coefficient of friction of the paired materials^56^. Nanocrystalline diamond is well known for its low friction coefficient when paired with nearly any other material, where typical friction coefficients are below^70–72^, or in the order of ~0.1. Furthermore, since the *σ*_yy_ magnitudes in the NDP obtained by the FE element model are nearly independent of the friction coefficient at the NDP/ML interface (*cf*. Suppl. Note 7), it is implied that tangential traction is not responsible for the tensile *σ*_yy_ found in the NDP. Following these arguments, the analytical treatment by M’Ewen can be effectively used to easily assess the cylindrical contact of ceramic materials with limited plastic deformation, without the need for elaborate FE models.

**Fig. 6:**
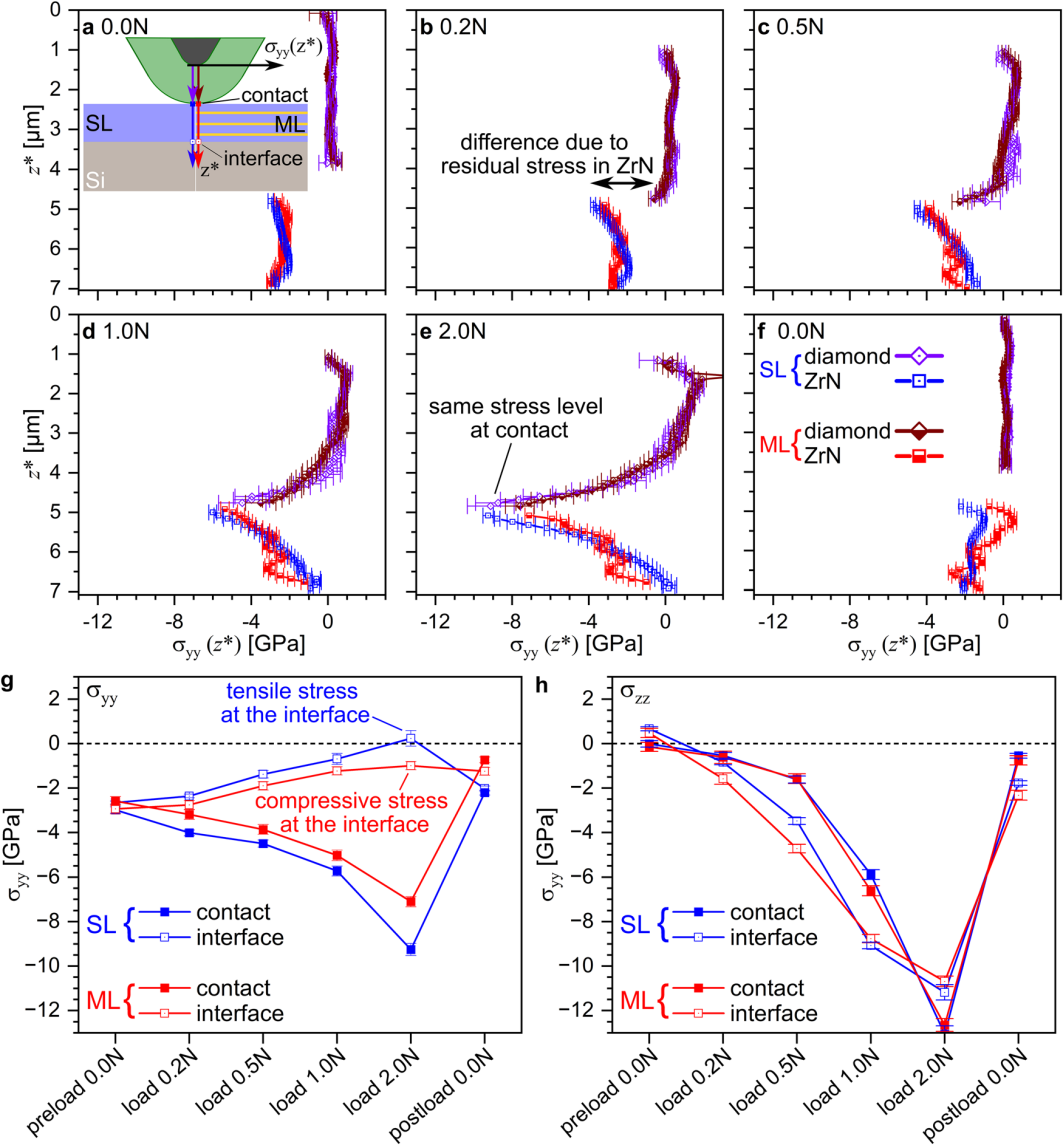
A detailed stress comparison of the SL and ML in contact with the NDP. A schematic of the individual analyses presented is shown in (**a**), representing the line intersections of the 2D stress fields presented in Fig. 4 in the center of the contact between the SL and ML films and the NDP. In (**a**), the *σ*_yy_ line profiles depicting the residual stress in the SL and ML as well as the NDP before the experiments. Note that the NDP is nearly stress-free before the experiment (**a**). The *σ*_yy_ line profiles across the indenter-sample interface are shown for the SL and the ML in contact with the NDP for the individual load steps of 0.2, 0.5, 1.0, and 2.0 N in (**b**–**e**), respectively, while the residual stress accumulation in the SL and ML films as well as the NDP after the experiment is presented in (**f**). The *σ*_yy_ and *σ*_zz_ stress values accumulated at the contact and the ZrN/Si interface are shown for the SL and the ML in (**g**, **h**), respectively, as shown in the schematic in (**a**).

For the second part of the discussion concerning the similarities and differences in the mechanical responses of the SL and the ML thin films, a direct comparison of several stress features throughout the indentation is presented in Fig. 6. As shown schematically in Fig. 6a, the vertical line profiles of *σ*_*yy*_ were extracted from the 2D stress data (Suppl Notes 3 and 4) at the *y*-position of the center of the contact between the NDP and the SL as well as the ML, and are presented together in Fig. 6a–f for all loading steps. Before loading, the residual stress of the NDP is centered at ~0 GPa for both experiments, while the average residual stress before loading of both thin films is ~−2.4 GPa (*cf*. Fig. 6a). During the initial stages of loading (Figs. 6b and 6c corresponding to loads of 0.2 and 0.5 N, respectively) the general stress distribution is similar for diamond in contact with the SL and ML thin films, while the thin films’ stress values themselves differ only by their initial residual stress levels (*cf*. Fig. 6a).

Generally, in addition to the residual stress in the SL and ML films, compressive and tensile stress are applied at the contact and the ZrN/Si interface throughout the loading, respectively. Additionally, oscillations of *σ*_yy_ are present in the ML thin film (Fig. 6a, in detail Fig. S12 in Suppl. Note 4), whose magnitudes increase with increasing load and highlight the plastic limits of the ZrCu interlayer. Beginning with the loading up to 1.0 N, the gap between the *σ*_yy_ in the diamond and the thin films reduces, which indicates irreversible deformation in the thin films close to the contact, after surpassing the yield stress of the thin films (Fig. 2a). While the onset of plastic deformation could be indirectly inferred from the von Mises stress surpassing the yield stress at a load of 1.0 N (Fig. S16, Fig. S10, Fig. 2a), only the NDP allows a direct comparison by assessing the stress magnitudes in both materials.

The stress gap across the interface vanishes at 2.0 N load (Fig. 6e), indicating high plastic deformation for both thin films close to the contact (in agreement with the regions of FWHM≫0.65 deg, discussed above). For the SL thin film indented by the NDP, a strong in-plane gradient of *σ*_*yy*_ is also present, ranging from −9.3 ± 0.3 at the contact with the probe to 0.2 ± 0.2 GPa at the ZrN/Si interface under the maximum applied load of 2.0 N (Figs. 4c, 6e, g). However, these stress levels have to be considered with respect to the residual stress in the SL ZrN, that already existed before the loading. This corresponds to a stress change of –6.3 ± 0.3 at the contact and +2.9 ± 0.3 GPa at the interface. Moreover, the tensile stress at the interface likely represents a lower-bound estimate, as a significant portion of the stress had already been relieved at the maximum load due to fracture of the Si and ZrN (Figs. 3a, 2c). On the other hand, the oscillations of *σ*_yy_ in the ML thin film become even more pronounced, introducing a stress variation of up to 2 GPa in the individual ZrN sublayers, while the observed stress magnitudes range from −7.1 ± 0.3 GPa to −1.0 ± 0.2 GPa. Here, for both experiments the crucial influence of the substrate material is revealed: While sc diamond (*E* ~ 1120 GPa^65^) is stiffer compared to nc diamond (E ~ 460 GPa^51^ by a factor of ~2.3, sc Si^73^ (E ~ 155 GPa) is more compliant than ZrN (E ~ 200 GPa, Fig. 2). Consequently, since Si is more compliant, a bending-like deformation is induced in the ZrN by the NDP, while the stiff diamond substrate in the probe itself leads to a higher localization of elastic deformation in the nc diamond (Fig. 4).

Finally, after the unloading, the diamond returns exactly to the same stress state present before the experiments, proving that no irreversible deformation was present within the nc diamond during both experiments (Fig. 6f). On the contrary, the *σ*_yy_ residual stress profiles in both the SL and ML thin films shift towards more tensile stress levels. However, the shift is less pronounced in the SL compared to the ML thin film, both at the ZrN/Si interface and the prior contact with the indenter (Fig. 6f). This indicates that the ability for inelastic deformation is comparatively limited in the SL thin film.

To further condense the comparison, the *σ*_yy_ and *σ*_zz_ stress values at the contact and the ZrN/Si interface are shown for the different loading states in the SL and the ML thin films in Figs. 6g, h, respectively, according to the schematic in Fig. 6a. While the *σ*_zz_ stress values are comparable throughout the experiments both at the contact with the NDP and the ZrN/Si interface (Fig. 6h), significant differences are found for the *σ*_yy_ stress component (Fig. 6g). During loading, both the accumulation of compressive and (more) tensile stress at the contact and the interface are less pronounced in the ML thin film in comparison with the SL thin film (Fig. 6g). This indicates that the ML thin film has a significantly reduced resistance against lateral deformation as a consequence of the ZrCu interlayers, which allows to accommodate the highest local loads without brittle failure across the ZrN/Si interface (Fig. 2).

**Fig. 7:**
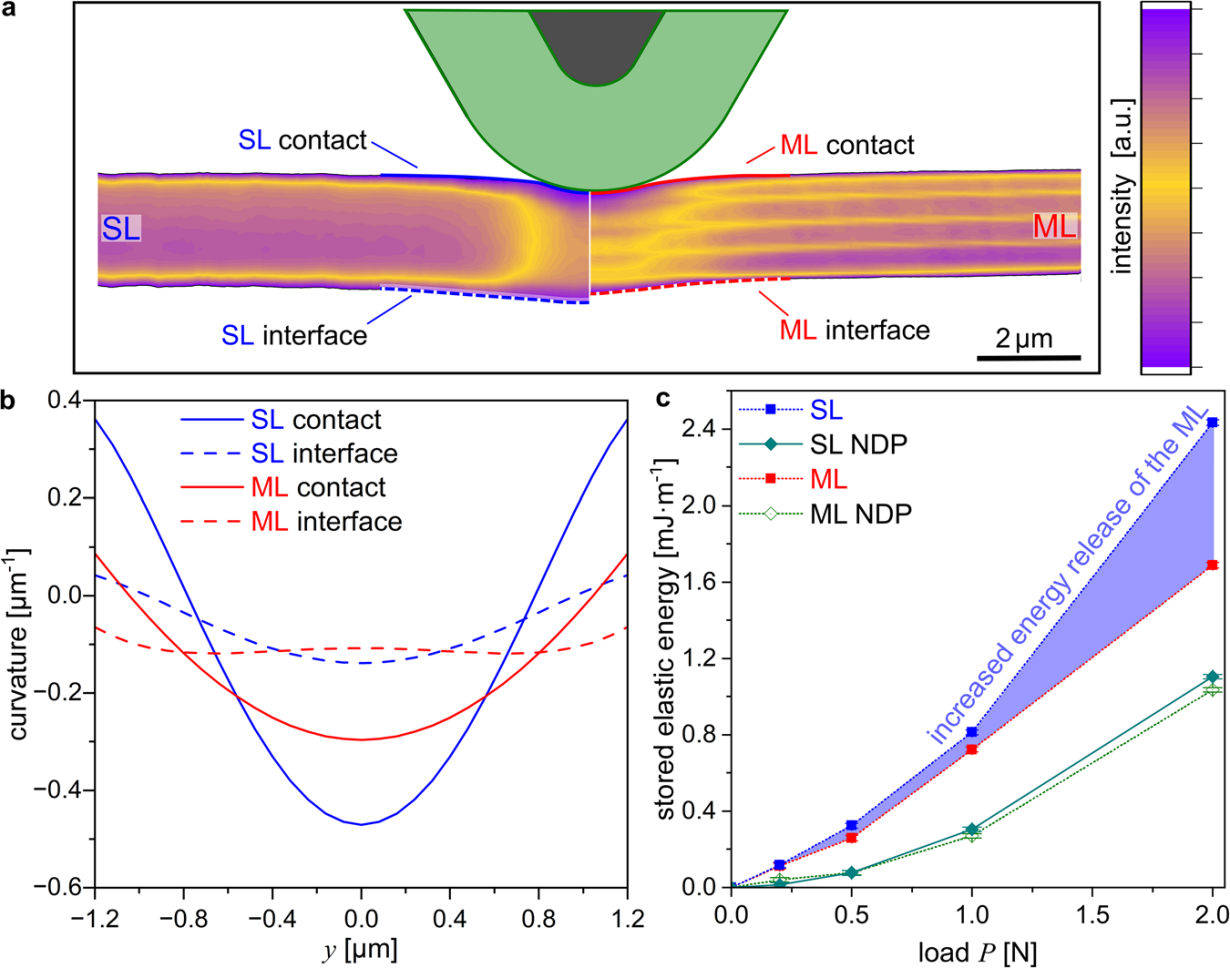
Geometrical response data and stored elastic energies within the SL and ML in contact with the NDP. In (**a**), the averaged intensities of the ZrN 111 DS ring retrieved from the SL and ML are presented together with a schematic of the NDP. The individual positions of curvature measurements for both films are also shown in (**a**), while the curvatures retrieved from the contact and the interface in the SL and ML films are presented in (**b**). The stored elastic energy per line element is shown for the NDP, the SL and the ML in (**c**), the difference represents the energy release of the ML film.

While a bending-like stress contribution was already indicated above by the elastic properties of the SL and ML films and the Si substrate, the magnitude of this bending contribution and its role can be estimated from the curvature (Fig. 7b), derived from the averaged diffraction intensity maps (Fig. 7a). To compare these, a hypothetical bending specimen should be considered, where the bending strain ${\varepsilon }_{{{\rm{yy}}}}=-{z}^{* }\cdot \frac{{\partial }^{2}w}{\partial {y}^{2}}$, with ${z}^{* }$ and $\frac{{\partial }^{2}w}{\partial {y}^{2}}$ being the distance from the neutral fiber and the curvature of this hypothetical bending specimen, respectively. Here, z* is positive in the upward direction. The curvature extracted from the intensity plots in Fig. 7a at the contact is significantly higher in the SL film compared to the ML film (Fig. 7b) and induces a compressive strain (z* > 0), consistent with the evaluated stress magnitudes (Figs. 4–6). If we assume that the neutral fiber in both films is centered, the negative curvature at the interface (Fig. 7b) introduces tensile strain. Consequently, the ratio between the maximum strains along the symmetry line is ${\varepsilon }_{{yy}}^{{{\rm{SL}}}}/{\varepsilon }_{{yy}}^{{{\rm{ML}}}}$ = 1.28 ± 0.08, *i.e*. a nearly 30% higher tensile contribution is added to the SL in comparison with the ML. Further simplifying the load state as simple bending (without support, thus neglecting the Si substrate), the same stress magnitudes with opposite signs should be induced on the top and bottom of the ZrN thin film at the contact. Hypothetically, adding the −6.3 GPa observed directly under the contact (Fig. 6e) as +6.3 GPa at the film-substrate interface would thus result in a total stress of 3.4 GPa after subtracting the −3.1 GPa residual stress (Fig. 6a).

This is arguably very close to the fracture stress evaluated by cantilever bending tests (Suppl. Note 2). Using the same argument for the ML film, a significantly lower bending stress of −4.5 GPa would be observed (Fig. 6), which would yield a total stress of 1.6 GPa at the film-substrate interface. However, in the experiment values much closer to 0 were observed in the SL film, while −1 GPa was evaluated from the ML film (Figs. 4–6), indicating that simple bending is not sufficient to describe the load state and/or that some degree of stress relief has taken place. In fact, both are true, since the Si substrate cannot be neglected as a support, and also cracking was observed as a stress relief mechanism (Figs. 2 and 3). Nevertheless, this simple argument is helpful to explain the build-up of tensile (bending-like) strain towards the substrate interface of the SL, which is precarious, since *σ*_yy_ is acting perpendicular to the columnar grain boundaries of low cohesive energy^30,31^. Thus, it is highly critical in terms of protective thin film failure. In contrast to this, in the topmost ZrN layer of the ML film, cracks were observed (Fig. 2d) in a region where exclusively compressive normal stresses are applied during loading and tensile residual stress is built up upon unloading (Sec. 2.2, Suppl. Note 4). Therefore, cracking in the topmost ZrN layer of the ML film may occur during tensile stress build-up upon unloading. Independent of the mechanical origin of the cracks in the film, their location within the films indicates the most critically stressed areas – the ZrN/Si interface and the surface in case of the SL and ML films, respectively.

The key distinction between the ML and SL films is evident here: in the ML film the *σ*_yy_ stress component beneath the indenter contact region remains compressive throughout film thickness (Fig. 4b), in contrast to the SL thin film (Fig. 4a). Crucially, outside the vertical extension of the contact area in the ML film, which is the zone of the highest *σ*_zz_ (Fig. 4d), the low magnitude of *σ*_yz_ (which is a representation of the rotation of the principal stress tensor) indicates that *σ*_yy_ is the lowest absolute principal stress. Since *σ*_zz_ is ≈ 0 outside the vertical extension of the contact (Fig. 4d), the stress response in the ZrN along the interlayer interfaces is thus limited to ${\sigma }_{{{\rm{yy}}}}$ ~−3 to −4 GPa, as can be seen from Fig. 4b. This indicates, that the ML thin film cannot accumulate stresses larger than ~3-4 GPa close to the ZrN/ZrCu interfaces, which is in excellent agreement with the ultimate tensile strength of ZrCu of 3.3 GPa found by cantilever bending tests^50,59^. Consequently, it is the yielding of ZrCu that reduces the transmission of compressive *σ*_yy_ stresses, instead promoting fracture along the columnar grain boundaries in the topmost layer (Fig. 2), thereby preventing the build-up of tensile stress at the ZrN/Si interface.

Finally, the elastic strain energy stored in the NDP, SL and ML films is considered in Fig. 7c. Since the elastic strain energy is inversely proportional to the Young’s modulus $E$ (nc diamond: *E* ≈ 460 GPa^51^, ZrN: *E* ≈ 200 GPa), the elastic strain energy in the NDP is ~2.3 times lower than in the ZrN (Fig. 7c). Given, that the stored elastic energy can be also expressed as $U=\int {Pdx}$, where *P* is the load and *x* is the displacement, similar strain energy densities can be estimated from the load-displacement data presented in Fig. 2b under the assumption of linear elasticity. That is actually the case for the NDP in contact with both the SL and ML films (*cf*. Fig. 7c). The comparably lower stress magnitudes in the NDP in contact with the ML film (Figs. 4–6) are made up by the comparably higher contact area again as can be seen from Fig. 3, cross-validating the experimental observations drawn from the thin films. On the contrary, a lower stored strain energy is present in the ML film after loading beyond 0.2 N. In total, the strain energy in the ML film at 2.0 N is only ~69% of the strain energy stored in the SL. Since plastic deformation is highly limited in the nanoceramic ZrN in both films and only possible in very small volumes at the highest multi-axial load, this strain energy difference is safely interpreted as the dissipation of strain energy through plastic deformation of the ZrCu interlayer. Consequently, the ~30% of dissipated plastic work within the ZrCu (Fig. 7c), can be regarded as a toughening mechanism, reducing the overall stress buildup and thereby enhancing the failure tolerance. Although the example of nacre has been employed extensively by materials scientists as an architectural design guideline, the basic principle remains valid: hard ceramics are cemented together with soft, deformable interlayers, allowing some degree of gliding and sliding while preserving overall cohesion^74–76^. Therefore, the results presented here show once more, even for protective thin films, that interfaces must be cohesive but interlayers deformable for improved failure tolerance - a property combination realized in the ML thin film. Furthermore, although the potential of the ML film was not captured by conventional laboratory thin-film testing, the energy dissipation during realistic deformation can be quantified using nanoscale X-ray diffraction-derived microstructure and stress data.

In summary, the approach presented here allowed us to quantify the mechanical and structural changes, including the multiaxial nanoscale dual-sided stress analysis in the single-asperity contact. Contrary to prior works, the nc diamond thin film on top of the sc diamond indenter tip enabled monitoring the mechanical response of both the indenter tip and the indented thin films within a single experiment with 80 nm resolution in bulk thin films. The influence of the various interfaces on the stress distribution was quantified and could be related to the elastic mismatch of the individual constituents. Additionally, it was shown experimentally how *tough interlayers function as stress dissipators* during multi-axial loading. Furthermore, this work demonstrates the mechanical and structural superiority of multi-layered soft–hard architectures, being able to dissipate 30% of the stored elastic energy compared to their monolithic counterparts, which might have been dismissed as inferior based solely on conventional laboratory mechanical characterization. The ML ZrN–ZrCu thin film effectively mitigates stress concentrations induced by the imprinting indenter through plastic deformation of the comparatively softer ZrCu interlayers, thereby preventing crack propagation across the ZrN/Si interface. These findings have implications not only for the design of protective thin films but also provide a mechanistic understanding of the protective function of hierarchical biological composites.

## Methods

### Thin film deposition

#### ZrN-ZrCu on Si

The monolithic ZrN (SL) and the multilayered ZrN-ZrCu (ML) thin films were deposited by unbalanced DC magnetron sputtering onto a Si(100) wafer held at a floating potential without any external heating. The substrates were rotated above the targets located at a target-to-substrate distance of 150 mm. The monolithic ZrN layers were deposited by magnetron sputtering of a Zr target (99.5% purity) in an Ar and N_2_ atmosphere at a pressure of 0.53 Pa. In the case of the ZrN-ZrCu ML, the same parameters were applied for depositing ZrN, while the ZrCu interlayers were deposited by non-reactive magnetron co-sputtering of Zr and Cu (99.99% purity) targets in pure Ar atmosphere at the same pressure. Here, the magnetron with the Zr target was operated in direct current regime, while the magnetron with the Cu target was operated in a high-power impulse regime. More technical details concerning the preparation of ZrCu and ZrN are given in refs. ^50,59,77^. and Suppl. Note 8.

#### Nanocrystalline diamond on single-crystalline diamond

The nanocrystalline (nc) diamond thin film was deposited on a wedge-shaped single crystalline (sc) diamond indenter tip with a tip radius of 2 µm, a length of 85 µm and wedge opening and side opening angles of 60 and 90 deg, respectively (Synton MDP, Nidau Switzerland) by modified HF-CVD^78^ and additionally onto a sc Si wafer with a diameter of 30 mm and a thickness of 530 µm. Ta filaments with a diameter of 3.6 mm were electrically heated to 2400 °C for thermal dissociation of the precursor gases hydrogen and methane. The Ta filament temperature was monitored by a Sensotherm Metis M316 pyrometer. For deposition of the nc diamond film on the indenter tip, the temperature of the sc diamond substrate of 800–850 °C was monitored by a thermocouple type K and a total gas pressure of 5 mbar was applied. The filament-to-substrate distance was maintained at 50 mm, while a CH_4_/H_2_ ratio of ~2% was used, yielding non-textured nanocrystalline diamond^51,79^. Deposition time was adjusted such that the final film thickness was ~3.8 µm. The indenter tip after deposition is shown in the Suppl. Note 10, Figures. S24 and S25.

### Sample preparation

#### FIB-preparation of the diamond tip

After the single-crystalline diamond was coated uniformly with nanocrystalline diamond, in order to obtain a uniform diffraction signal, the side faces perpendicular to the wedge (Fig. 1) were removed and polished by focused ion beam (FIB) milling in an Auriga system (Carl Zeiss AG, Oberkochen, Germany) applying a current of 5 nA at a voltage of 30 kV. The indenter tip after final preparation is shown in the Suppl. Material.

#### Preparation of the cross-sectional lamellae for the CSnanoXRD experiment

Cross-sectional lamellae used for the indentation experiment were prepared by consecutive mechanical polishing, femto-second laser ablation, and FIB milling in an Auriga laser system (Carl Zeiss AG, Oberkochen, Germany), applying final polishing currents for the cross-sections of 500 pA at an acceleration voltage of 30 kV.

### FIB-SEM analysis

FIB cross-sections of the pristine and indented films were prepared by FIB milling in an Auriga system (Carl Zeiss AG, Oberkochen, Germany). Prior to polishing a Pt protection layer was applied using the gas injection system of the workstation. Consecutively decreasing currents between 500 and 50 pA at an acceleration voltage of 30 kV were used for polishing the cross-section. Final removal of FIB-induced defects was performed by using a current of 50 pA at an acceleration voltage of 5 kV under an inclination of 15 deg. The same parameters were used for the preparations of the cross-sections of the spherical indents. The cross-sections were imaged in a LEO1525 scanning electron microscope (SEM) (Carl Zeiss AG, Oberkochen, Germany) using an accelerating voltage of 4 kV and an aperture of 20 µm.

### Micromechanical testing

Hardness and indentation modulus of the SL ZrN and ML ZrN-ZrCu thin films were determined from load-displacement curves measured using a Fischerscope H100 microindentation system equipped with a Vickers diamond tip at a constant load of 10 mN. Micromechanical cantilever bending tests were carried out on both materials using a FT-NMT04 indenter (Oxford Instruments PLc., Tubney Woods, Abingdon, UK) using a 20 mN wedge-shaped load cell and performed similar to the procedure detailed in refs. ^22,80–82^. and again summarized in Suppl. Note 9. Nanoindentation tests on the nanocrystalline diamond were performed using a G200 nanoindenter (KLA Corporation, Milpitas, California, USA) equipped with a continuous stiffness measurement (CSM) unit and different indenter tip geometries (Synton-MDP, Nidau, Switzerland). Diamond Berkovich indentation experiments were carried out in strain-rate controlled mode (0.05 s^−1^) to a maximum indentation depth of 500 nm. Hardness was evaluated according to the Oliver–Pharr method^37^ and averaged over the depth range of 420–480 nm. Spherical nanoindentation experiments were conducted using sphero-conical diamond indenter with 5 µm radius following the experimental procedure described by Leitner et al.^83,84^, with a preset strain rate of 0.001 s^−1^ and a maximum indentation depth of 750 nm.

### CSnanoXRD experiment and analysis

The CSnanoXRD experiments were performed at the nanofocus extension of the beamline ID13 of the European Synchrotron Radiation Facility (ESRF) in Grenoble, France^85,86^. A pair of multi-layer Laue lenses working with a vertical and horizontal focus size of 75 nm and a focal depth of 50 µm, were used along with the dedicated indentation setup^45^ available at the beamline. A full description of the indentation setup is provided in the Suppl. Note 11. An X-ray photon energy of 13.0 keV equivalent to a wavelength of 95.3725 pm was employed.

Prior to the experiment, the interface between the sc and nc diamonds, as well as the interface between the thin films and the Si substrates were individually aligned parallel to the incident X-ray beam by performing a set of absorption line scans along the *y* and *z*-axes at various sample orientations *φ* and *ξ* using a passivated, implanted, planar silicon diode to measure transmitted X-ray intensities. The optimal sample position and tilt were determined by maximizing the absorption contrast between the individual layers and is detailed in Suppl. Note 11.

Figure 1 shows a schematic of the setup with the orientation of the synchrotron thin film and the coated diamond tip relative to the beam and the detector, with the corresponding coordinate system. Maps of diffraction patterns were collected across the indenter sample interface in *y*- and *z*-directions before loading and at incremental loads of 0.2, 0.5, 1.0, and 2.0 N, as well as after unloading. Thereby, a total of 37,500 2D diffractograms were recorded for each load step on an Eiger X 4 M hybrid pixel detector from within a mapped region of 20 µm × 12 µm (*y* × *z*), corresponding to a scanning step size of 80 nm. The exact detector geometry with respect to the samples was calibrated using a NIST corundum powder^87^ yielding a sample-to-detector distances of 102.52 and 109.97 mm, for investigation of the SL and ML film, respectively. The 2D diffractograms were integrated using the pyFAI software package^88^.

The diffuse scattering at relatively small diffraction angles, i.e., small-angle X-ray scattering (SAXS), around the beam stop (Fig. 1) originates primarily from electron density variations, such as alternation of materials, presence of grain boundaries, interfaces, cracks, precipitates and pores with sizes of $\sim \lambda /\theta$ where $\lambda$ represents the X-ray wavelength and $\theta$ is the Bragg angle^52,89^. In the present case, the signal scattered onto the 2D detector at diffraction angles between ~0.05 and ~0.5 deg was integrated radially (*θ*) and azimuthally ($\delta$) in order to obtain qualitative information primarily on the occurrence of the cracks within the coated indenter and samples.

In order to evaluate ${{\rm{FWHM}}}(y,z,{\Delta \delta }_{g})$ and stress tensor distributions ${\sigma }_{{ij}}\left(y,z\right)$ (with *i*,*j* ϵ *x*,*y*,*z*) an integration of the diffraction patterns was performed over the azimuthal angle $\delta$ in ${\Delta \delta }_{g}$ segments (termed *cakes*) of 10 deg width. Thus, 36 radial intensity distributions $I(\theta ,{\Delta \delta }_{g})$ were obtained for each diffractogram in each cake $g$. The diffraction angles of ZrN 111, 200, 220 and 311 DS rings as well as the diamond 111 DS ring ${\theta }^{m,{hkl}}\left({\Delta \delta }_{g}\right),{g}=[{\mathrm{1,36}}]$ and the ${{{\rm{FWHM}}}}^{m}(y,z,{\Delta \delta }_{g})$ were determined by peak fitting using a Pseudo-Voigt function for each exposure in every cake $g$, with *m* denoting the material and *hkl* the Miller indices of the respective DS ring.

Generally, the FWHM evaluated from DS rings is sensitive to the size of coherently diffracting domains, the presence of structural defects like dislocations, vacancies, and other types of micro- and nanoscopic crystal lattice distortions, which can be denoted as microstrains of 2^nd^ and 3^rd^ order, as well as gradients of strains of 1^st^ order within the X-ray gauge volume. Thus, the averaged ${{\rm{FWHM}}}(y,z)\,=\,\frac{1}{36}{\sum }_{g=1}^{36}{{\rm{FWHM}}}(y,z,{\Delta \delta }_{g})$ is a vital qualitative parameter to indicate defect accumulation in a deformed volume.

The intensities ${{\rm{I}}}(y,z,{\Delta \delta }_{g})$ of the ZrN 111 DS where azimuthally averaged, giving the ${{\rm{I}}}(y,z)\,=\,\frac{1}{36}{\sum }_{g=1}^{36}{{\rm{I}}}(y,z,{\Delta \delta }_{g})$ to assess the contour of the thin films during deformation. The resulting surface and interface contours where then fitted with a 6^th^ order polynomial, which was differentiated twice to obtain the surface and interface curvatures.

The quantification of stresses based on CSnanoXRD data was performed according to the methodologies presented in detail in earlier works^45,46,53,90,91^. Specifics and a recapitulation of the procedures are further given in Suppl. Note 11.

### Analytical and eigenstrain-based FE analysis to recreate the stress distribution in the NDP and the SL and-ML thin films

The contact problem given by the NDP and the SL thin film was interpreted by the elastic solution of the cylindrical contact given by Hertz^1,56^ using the exact solution for the 2D stress fields given by M’Ewen^55^. This approach is summarized in the Suppl. Note 12. The contact problem was further simulated using the finite element (FE) method for the ML film in contact with the NDP using the commercial software package Abaqus 2023 (Dassault Systèmes Simulia, Vélizy-Villacoublay, France). Unlike conventional process models, the simulation was designed to accommodate the eigenstrains^57,58,92^ inherited from the deposition of the SL and ML thin film layers. This involved defining the eigenstrain field assumed to vary only along the surface normal ($z$-axis) expressed as a high-order polynomial function as outlined in Suppl. Note 13. The NDP was simulated without considering residual stress in agreement with the experimental data (Suppl. Notes 3 and 4).

The indentation process was simulated by FE modelling of the contact between the indenter and the multilayer. All details of the simulation are summarized in Suppl. Note 13, while elastic constants are given in Table S1.

## Supplementary information

Transparent Peer Review file

Supplementary Material

## Data Availability

The raw data generated in this study have been deposited in the ESRF database under accession codes 10.15151/esrf-dc-2439292038 and 10.15151/ESRF-DC-2439293152. Processed research data for this work are available from the authors upon reasonable request.

## Code availability

The codes that support the findings of this study are available from the corresponding authors upon reasonable request.
