# Supplementary material for: How interfaces limit nanoscale stress concentrations and prevent catastrophic failure in single-asperity contacts
Source: Commun Mater. 2026 Jun 30;7(1):194. doi: 10.1038/s43246-026-01239-1 (PMC13391362; doi:10.1038/s43246-026-01239-1)
Supplement: Supplementary file 2 — Supplementary Material [file 43246_2026_1239_MOESM2_ESM.pdf]

Supplementary material for

How Interfaces Limit Nanoscale Stress Concentrations and Prevent Catastrophic Failure in Single-Asperity Contacts

Michael Meindlhumer* *et al.*

*Corresponding author. Email: michael.meindlhumer@unileoben.ac.at

This PDF file includes:

Supplementary Note 1: SEM analysis of pristine and indented thin films 2
Supplementary Note 2: Micromechanical data obtained from the SL and ML thin films 4
Supplementary Note 3: Detailed CSnanoXRD analysis of the SL thin film 8
Microstructure and residual stress of the SL thin film and the nanomechanical diamond probe before loading 8
Microstructural changes and stress distributions of the SL thin film and the NDP in contact at 0.2 N load 12
Microstructural changes and stress distributions of the SL thin film and the NDP in contact at 0.5 N load 14
Microstructural changes and stress distributions of the SL thin film and the NDP in contact at 1.0 N load 16
Microstructural changes and residual stress changes of the SL thin film and the NDP after the experiment 18
Supplementary Note 4: Detailed stress analysis for ML thin film 20
Microstructure and residual stress of the ML thin film and the NDP before loading 20
Microstructural changes and stress distributions of the ML thin film and the NDP in contact at 0.2 N load 23
Microstructural changes and stress distributions of the ML thin film and the NDP in contact at 0.5 N load 25
Microstructural changes and stress distributions of the ML thin film and the NDP in contact at 1.0 N load 28
Microstructural changes and residual stress changes of the ML thin film and the NDP after the experiment 31
Supplementary Note 5: σ_{zz} vertical virtual sections revealing the stress transfer across the interface 33
Supplementary Note 6: Stress distributions obtained by applying the Hertzian Theory 35
Supplementary Note 7: Detailed Finite Element analysis 37
Supplementary Note 8: Deposition of the ZrN and ZrCu layers 42
Supplementary Note 9: Preparation and testing of the ZrN and ZrN-ZrCu microcantilevers 43
Supplementary Note 10: Preparation of the NDP 44
Supplementary Note 11: *In situ* CSnanoXRD setup, NDP and sample alignment and stress analysis method 46
Supplementary Note 12: Analytical elastic and hardness considerations of the cylinder contact 49
Supplementary Note 13: Eigenstrain-based FE analysis to recreate the stress distributions in the ML thin film 51
References 53

Supplementary Text
Figs. S1 to S26
Tables S1

Supplementary Note 1: SEM analysis of pristine and indented thin films

FIB-polished cross-sections of the pristine single-layer ZrN (further denoted as SL) and the multi-layered ZrN-ZrCu (further denoted as ML) thin films are presented in Fig. S1a and S1b, respectively. Here, the SL film exhibits typical columnar grain morphology with highly elongated grains reaching nearly throughout the entire film thickness (Fig. S1a), which may be an indication of underdense growth[1]. Additionally, this indication is fostered by the occurrence of pores at the grain boundaries, which is a typical feature of Mode I thin film growth[1,2].

Furthermore, also the ~500 nm thick ZrN layers of the ML film exhibit a columnar grain morphology, yet in this case the growth of the individual crystallites is interrupted by the amorphous ZrCu interlayers of ~20 nm thickness (Fig. S1b). The roughness of the interfaces increases with increasing film thickness, which is a consequence of the faceted growth of the ZrN crystallites. Additionally, some pores at columnar grain boundaries are also visible in Fig. S1b, yet in case of the ML film they are less prevalent compared to the SL film.

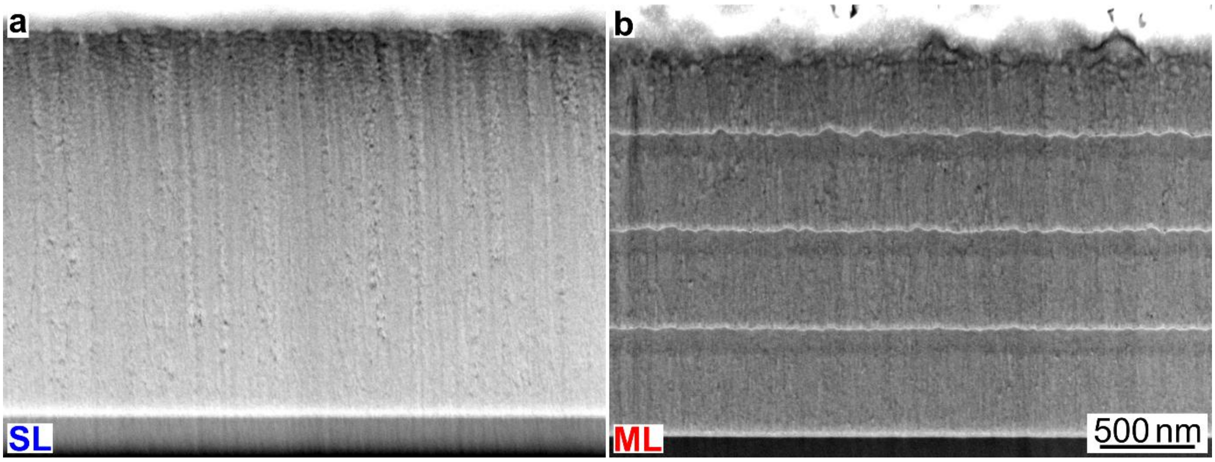

Figure S1. FIB-polished cross-sections of pristine thin films. In (a) the pristine monolithic ZrN thin film is presented, while in (b) the multilayered ZrN-ZrCu film is shown. The scale bar in (b) is applicable to (a) and (b).

After the *in situ* experiment, FIB-polished cross-sections of the deformed SL and ML thin film regions were prepared and low-resolution SEM images are presented in Fig. S2a and S2b, respectively. The FIB cross sections presented in Fig. S2 show vertical cracks in the Si substrate for both the SL and the ML thin film, in agreement with vertical high-intensity features found in the small-angle X-ray scattering microscopy (SAXSM) data presented in Figs. 4a and 5a for the SL and the ML thin film, respectively. The concerted presence of the cracks found in the FIB cross-sections with the high intensity line feature (Figs. 4a and 5a) supports the conclusion that crack formation and growth was observed directly in the SAXSM micrographs.

Despite the obvious agreement of failure in the substrate, the differences between the thin film failure modes observed for the SL and the ML films are striking. As can be seen from Fig. S2,

crack growth in the SL film starts at the interface between thin film and substrate, which is clearly devastating for the structural stability of the entire system (Fig. S2a). On the contrary, fracture in the ML film is only observed in the topmost ZrN and crack growth is stopped at the first ZrN/ZrCu interface.

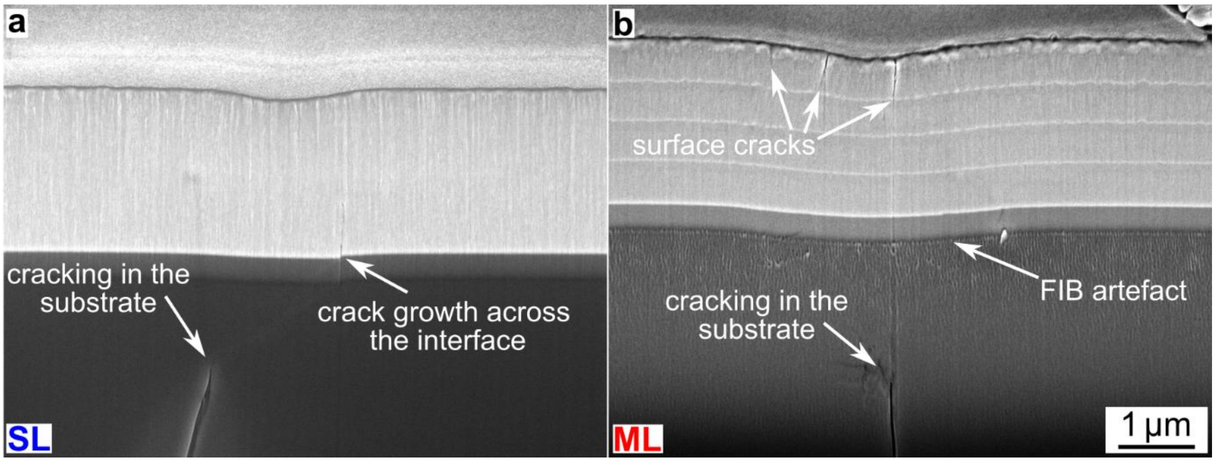

Figure S2. FIB-polished cross-sections of the indented thin films. In (a) the indented region of the monolithic ZrN thin film is presented, while in (b) the residual deformation in the multilayered ZrN-ZrCu film is shown. The scale bar in (b) is applicable to (a) and (b).

Supplementary Note 2: Micromechanical data obtained from the SL and ML thin films

Micromechanical cantilever bending experiments (*cf.* Methods, Suppl. Note 9) were carried out on both the SL and the ML thin films. The resulting load-deflection curves of the experiments are presented in Fig. S3 and reveal a linear-elastic deformation, i.e. no signs of plastic deformation were observed. While the curves were used to evaluate the Young's Modulus E and the fracture stress σ_F, the fracture toughness K_{IC} was evaluated from the notched cantilevers. Specifically, Young's moduli of, 195±6 and 215±18 GPa as well as fracture strengths of 3.9±0.1 and 3.9±0.6 GPa were evaluated from testing the unnotched cantilevers (Fig. S3a) milled from the SL and ML films, respectively. Additionally, fracture toughnesses were evaluated as 2.6±0.3 and 2.6±0.1 MPa·m$^{0.5}$, by testing the notched cantilevers prepared from the SL and ML films, respectively. The average σ_F and K_{IC} values are presented in Fig. S3a and b, respectively, as well. In total, the thin films have similar micromechanical properties.

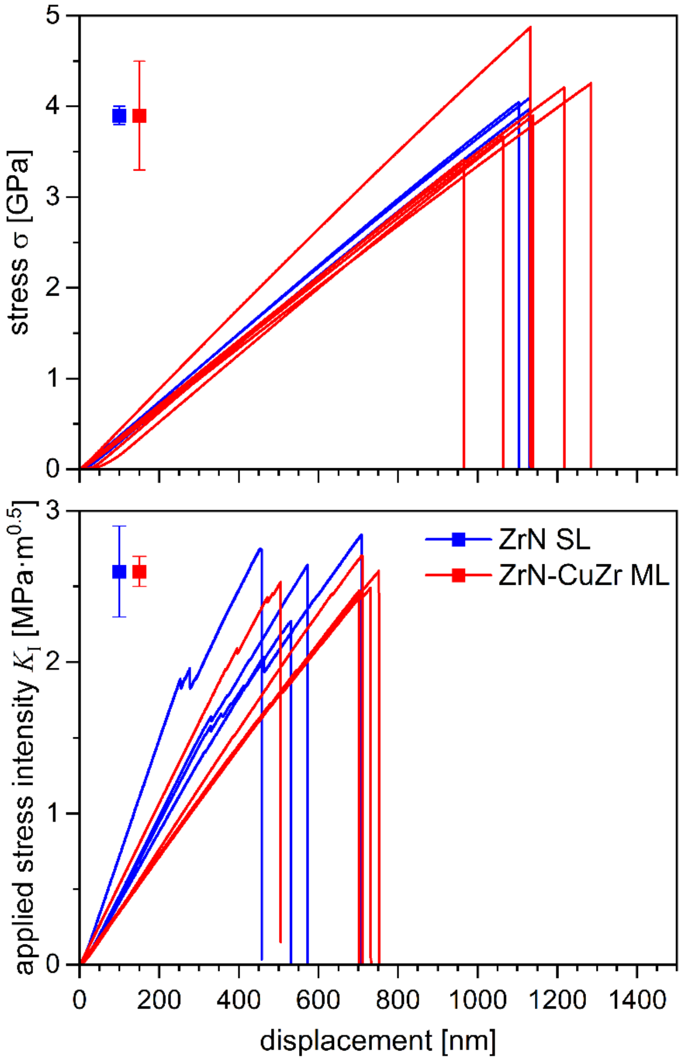

Figure S3. Load-displacement data obtained from micromechanical cantilever tests performed on the SL and ML thin films. Load-deflection curves recorded during the micromechanical tests on unnotched and notched cantilevers are presented in (a) and (b), respectively.

Ex situ fractography performed on the fracture surfaces of the tested cantilevers is presented in Fig. S4 and confirmed linear-elastic fracture along underdense column grain boundaries for both the SL and the ML thin films. Furthermore, in case of the multilayer film, it showed that the morphology is preserved across the ZrCu interlayer, i.e. the morphological appearance of the grains does not differ below and above a ZrCu interlayer. This similar morphological appearance of the SL and ML thin films concurs with the similar micromechanical properties obtained by the fracture tests themselves (Fig. S3).

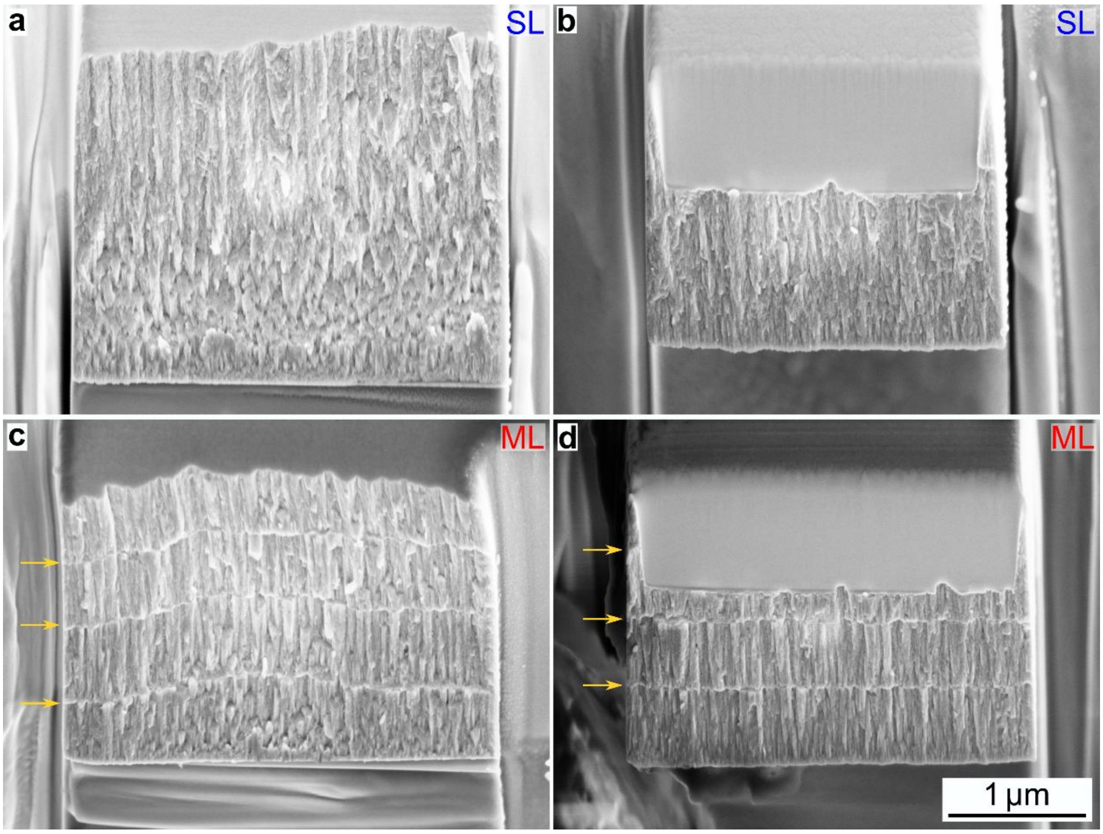

Figure S4. Fracture surfaces evaluated using SEM after the *in situ* micromechanical cantilever bending experiments. While representative fracture surfaces of unnotched and notched cantilevers of the SL thin film are shown in (a) and (b), respectively, fracture surfaces of unnotched and notched cantilevers of the ML thin film are shown in (c) and (d), respectively. The golden arrows in (c) and (d) indicate the ZrCu interlayers. The scale bar in (d) is applicable to all images.

Furthermore, spherical nanoindentation was performed with a sphero-conical indenter of 5 µm radius, to improve the statistical analysis of the microstructural response of the SL and ML thin films. Six indents were carried each out on both films, which yielded similar microstructural and mechanical responses. The results obtained by spherical nanoindentation using a tip with a radius similar to the nanomechanical diamond probe can be also interpreted as a single-asperity contact[3] and are presented in Fig. S5. First, the surface morphology of the indents performed on the SL and ML indents is presented in Fig. S5a and S5b, respectively, which is nearly identical for both thin films. Additionally, cross-sections were prepared from the indents on the SL and ML films by FIB milling and are presented in Fig. S5c and S5d, respectively. In the cross-sectional view, the difference in the mechanical response of the SL and ML films is highlighted by the different depth-dependent microstructural changes. In case of the SL film, the curvature imposed by the impinging indenter is nearly directly transferred to the substrate,

which results in fracture of the Si substrate and subsequently a step at the interface (Fig. S5c). This behavior is similar to the microstructural response of the SL found after the indentation with the NDP (Fig. 2d, Fig. S2a). On the other hand, the ZrCu interlayer is apparently more flexible, and the curvature of the interlayers is gradually decreasing towards the substrate, effectively reducing the deformation imposed on the substrate (Fig. S5d). Finally, the stress-strain curves evaluated from the spherical nanoindentation are presented for the SL and ML films in Fig. S5e and S5f, respectively. Highly different stress-strain curves evaluated from the spherical indents, where yield stresses of 9.8±0.7 and 5.4±0.4 GPa were evaluated from the SL and ML films, respectively. While the composite yield stress of the ML film is significantly lower, after the onset of plastic deformation, a linear hardening is observed (Fig. S5f) and the observed stress at maximum indent depth is only slightly smaller compared to the SL film (Fig. S5e).

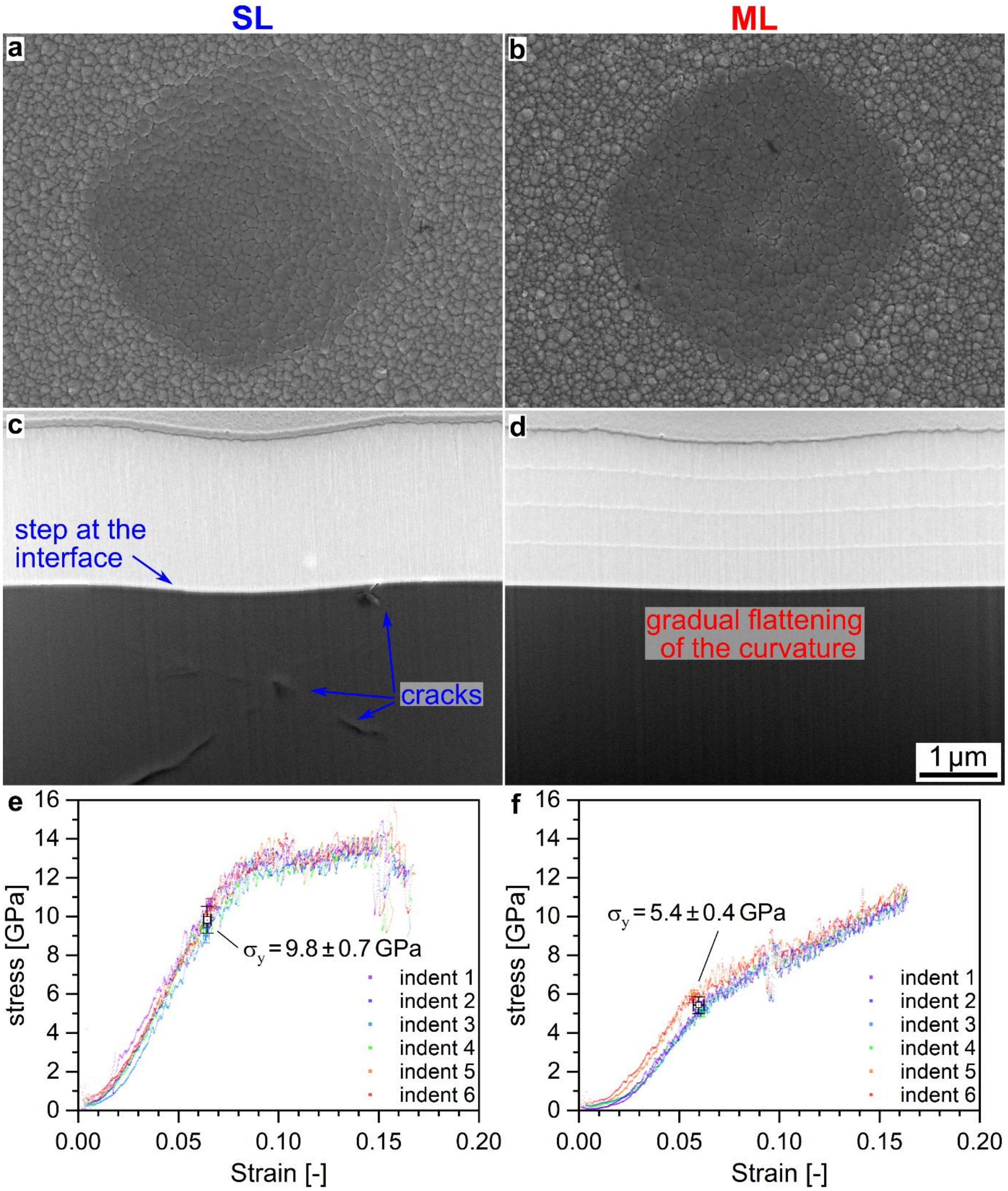

Figure S5. Data obtained from spherical nanoindentation on the SL and ML thin films. In (a) and (b), post indent SEM micrographs taken from the indents surface on the SL and ML films are shown, while in (c) and (d) FIB milled cross-section of the indents on the SL and ML films are presented, respectively. Stress-strain curves obtained from the spherical nanoindentation are presented in (e) and (f) for the SL and ML films, respectively. The scale bar in (d) equals 1 μm and is applicable to all images.

Supplementary Note 3: Detailed CSnanoXRD analysis of the SL thin film

Microstructure and residual stress of the SL thin film and the nanomechanical diamond probe before loading

The experimental microstructure and stress data of the SL film before contact with the nanomechanical diamond probe (NDP) are presented in Fig. S6. In the phase plot shown in Fig. S6a, where the intensities are integrated azimuthally (Fig. 1), only peaks corresponding to the cubic B1 ZrN phase can be detected. The azimuthal intensity distributions of the ZrN 111 Debye-Scherrer (DS) ring are presented in Fig. S6b and reveal a gradual change of the azimuthal intensity distribution along the 111 DS ring with increasing film thickness. At the beginning of the growth a competition between 200 and 111 oriented grains can be seen, visible as azimuthal maxima at ±55 as well as ±20 and ±90 deg, respectively. In later growth stages, only maxima corresponding to a 111 fiber texture are detected, but with an increasing symmetrical shift away from the ideal positions. This can be interpreted as a gradual transformation from 200 and 111 fiber textures towards a complex preferential orientation close to a 111 fibre texture, in agreement with literature[4–6].

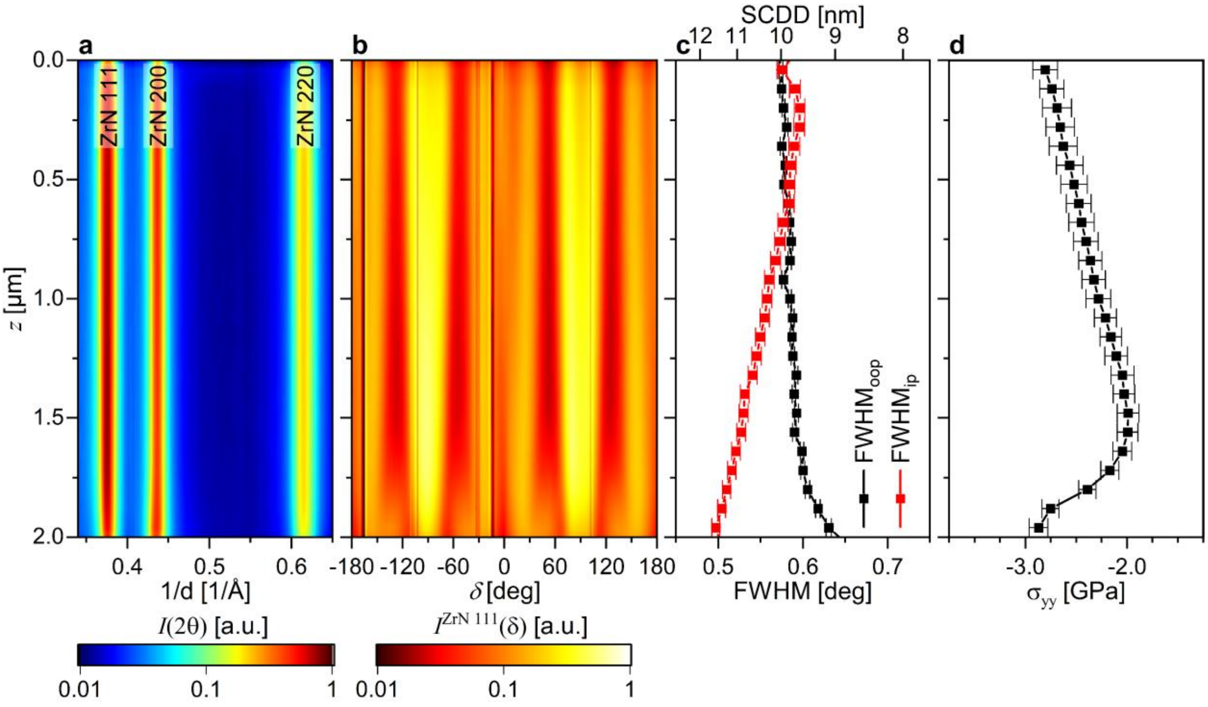

Figure S6. CSnanoXRD data retrieved from the SL thin film before the experiment. In (a) and (b) the phase plot depicting the ZrN 111, 200 and 220 reflections and the azimuthal distribution of the diffracted intensities of the ZrN 111 reflections are presented, respectively. The dark vertical intersections in (b) are due to the gaps between the individual segments of the Eiger 4 M detector. The cross-sectional FWHM variation in the out-of-plane (oop) and in-plane (ip) orientations are shown in (c), while the residual stress evaluated from the ZrN 111 DS ring is presented in (d).

The FWHM was evaluated along the films in-plane (*y*-direction in Fig. 1) and out-of-plane (*z*-direction in Fig. 1) directions to get qualitative insight into the morphology of the SL ZrN thin

film. The observed cross-sectional evolution is typical for thin films with a gradual development of microstructure[4–8], which shows mutual increase and decrease of size of coherently diffracting domains and FWHM in the out-of-plane orientation (Fig. S6c). On the other hand, the FWHM in the in-plane orientation gradually increases, which is likely related to the gradual cross-sectional change in texture and the associated changes in defect densities (compare Fig. S6b and S5c). Finally, the cross-sectional evolution of the in-plane residual stress is presented in Fig. S6d and shows a decreasing residual stress magnitude ranging from -2.87±0.09 GPa to -1.99±0.10 GPa at the Si/ZrN interface up to a film thickness of 0.5 µm, respectively. With further increasing film thickness, the residual stress magnitude gradually increases and reaches a value of -2.80±0.12 GPa at the film's surface, which is also attributed to the gradual cross-sectional development of morphology and texture across the film thickness (Fig. S6).

Microstructural changes in the thin films are investigated by small-angle X-ray scattering microscopy (SAXSM), which highlights the variation of electron density within the X-ray gauge volume, and allows to qualitatively identify the amount of pores, interfaces, cracks and different materials by the scattering contrast. In the SAXS micrographs of the SL thin film presented in Fig. S7a, a wedge-shaped structure of lower scattered intensity can be seen in the diamond indenter, while a uniform SAXS intensity is found outside the immediate tip region. This lower intensity represents the growth of micron-sized oriented diamond crystals and is directly related to the growth conditions of the CVD diamond layer (*cf.* Methods, Suppl. Note 10). Furthermore, a gradual decrease of the SAXS intensity can be seen in the ZrN layer (Fig. S7a), which can be interpreted as a decrease of grain boundaries and other defects in the thin film due to the competitive columnar grain growth[4–8].

In order to interpret the microstructural changes during indentation of the SL and ML thin films, the azimuthally averaged FWHM of the ZrN 111 and diamond 111 DS rings were collated in a 2D contour plot, which is presented in Fig. S7b. As can be seen from Fig. S7b, the FWHM of the nanocrystalline (nc) diamond is lower directly at the indenter tip, with an opening angle of 90 deg enclosing the area of decreased FWHM. This can be interpreted by the growth conditions of diamond during CVD, where the tip was facing the hot filaments of the deposition chamber during deposition (see also Suppl. Note 10). In the case of ZrN, the gradual increase of the FWHM concurs with the orientation-dependent analysis presented in Fig. S6, above and is a representation of the columnar thin film growth[4–8]. The average of the azimuthally averaged FWHM in the ZrN before the *in situ* experiment was 0.566±0.033 deg. Therefore, a boundary FWHM value of >0.65 deg should be valid to describe zones of highest deformation, since it is separated from the mean value by at least 2.5 times the standard deviation.

Before contact, the nc diamond forming the nanomechanical probe was essentially free of residual stress, as can be seen from the 2D contour plots of the σ_{yy} and σ_{zz} stress components presented in Fig. S7c and d, respectively. Residual stress of nc diamond deposited on Si with the exactly same deposition conditions was characterized by cross-sectional X-ray nanodiffraction and quantified as ~0.5 GPa[9]. Taking into account the thermal expansion coefficients of Si[10] and nc diamond[11], the thermal and intrinsic stress components were estimated as -0.1 to -0.35 GPa and +0.6-0.85 GPa, respectively. Taking into account the lower thermal expansion coefficient of single crystalline diamond[12–15], a thermal stress component of ~-0.9 GPa can be estimated for nc diamond deposited on sc diamond. Consequently, summing up the thermal and intrinsic stress components for the nc diamond grown on sc diamond, the experimentally observed absence of residual stress (Fig. S7c and d) is in agreement with the growth conditions present in the literature. In addition, the shear stress σ_{yz} showed a separation in positive and negative shear, which indicates that the principal stress tensor in the nanomechanical probe is oriented parallel to its surface normal. On the contrary, the SL thin film exhibits relatively large in-plane residual stress (Fig. S7c) with a distribution similar to the one outlined in Fig. S6d and an average value of -2.42±0.29 GPa. Furthermore, a slight gradient of the σ_{zz} stress component ranging from 0.66±0.09 GPa to 0±0.12 GPa at the Si/ZrN interface and the film surface, respectively. This is interpreted by the gradual development of d_0, which was not corrected in the evaluation (*cf.* Methods) However, the σ_{zz} magnitudes are low compared to the applied stresses and do not impair the conclusions drawn from this work. The σ_{yz} magnitudes evaluated from the ZrN thin film are close to 0 (Fig. S7e), which further indicates the presence of exclusively in-plane oriented residual stress.

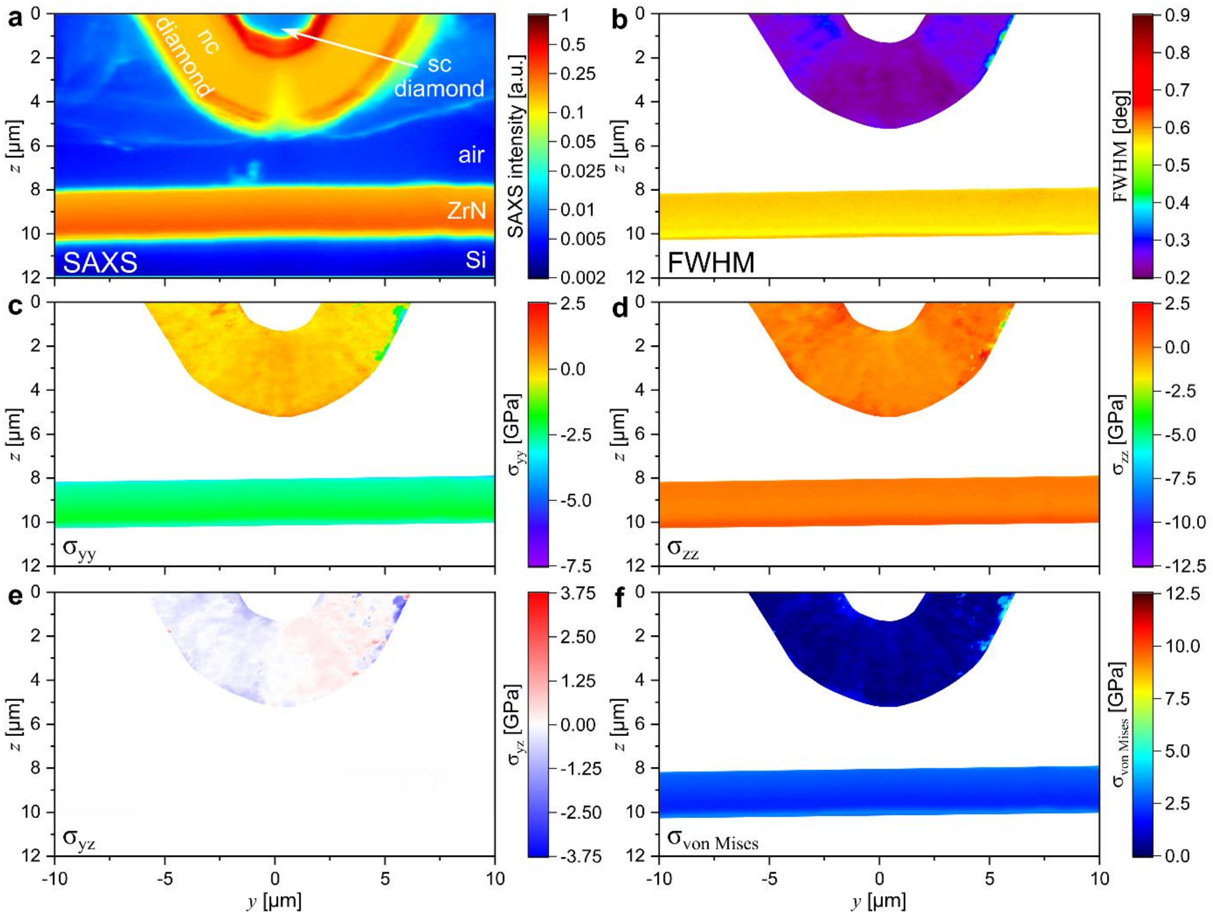

Figure S7. CSnanoXRD data obtained from the NDP and the SL film before loading. In (a) and (b) the SAXSM and the FWHM micrographs are shown, while in (c-e) the σ_{yy}, σ_{yz} and σ_{zz}, are presented, respectively. Finally, in (f) the $\sigma_{von\ Mises}$ calculated from the stress components is displayed.

Finally, the von Mises stress $\sigma_{von\ Mises}$ magnitudes are presented in Fig. S7f, which are close to 0 and centered around the residual stress values discussed above in the case of the nc diamond thin film and the SL ZrN thin film, respectively.

Microstructural changes and stress distributions of the SL thin film and the NDP in contact at 0.2 N load

The SAXS micrographs of the SL thin film in contact with the NDP at a load of 0.2 N is presented in Fig. S8a. Despite the NDP being in contact with the thin film, no changes of the SAXS intensities can be found in comparison to the state prior to loading (Fig. S7a).

The azimuthally averaged FWHM of the ZrN 111 and diamond 111 DS rings are shown in Fig. S8b. There, a slight increase of the FWHM in the SL film can be seen in a roughly circular region under the contact, while no apparent changes can be seen in the nc diamond (Fig. S8b). Since the applied stresses discussed below are not high enough to induce plastic deformation (a hardness of 21 GPa (Fig. 2) equals a yield stress of 7 GPa considering a Tabor[16,17] factor of 3), the increase of the FWHM can be related exclusively to gradients of stresses of 1st order in the X-ray gauge volume[18,19] and thus already indicate the zones of highest deformation.

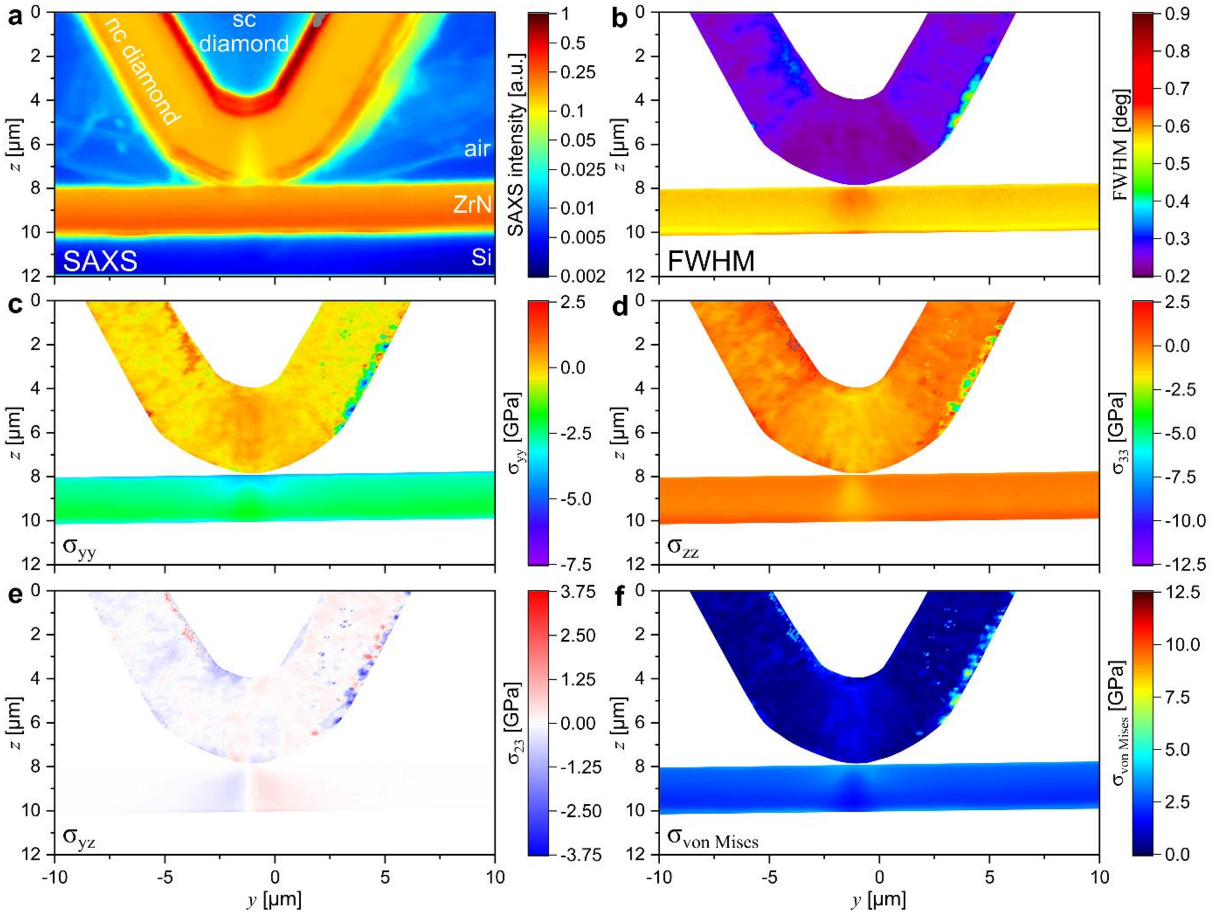

Figure S8. CSnanoXRD data obtained from the interaction of the NDP with the SL film at a load of 0.2 N. In (a) and (b) the SAXSM and the FWHM micrographs are shown, while in (c-e) the σ_{yy}, σ_{yz} and σ_{zz}, are presented, respectively. Finally, in (f) the $\sigma_{von\ Mises}$ calculated from the stress components is displayed.

At 0.2 N load, compressive σ_{yy} distributions are introduced in the SL ZrN thin film and the NDP close to the contact (Fig. S8c), which rise σ_{yy} to -3.77±0.13 GPa and -0.48±0.28 GPa, respectively. In case of ZrN, compressive stress is introduced in addition to the residual stress

of ~-3 GPa already present before, thus approximately similar stress magnitudes are added for both the diamond and the ZrN. Furthermore, at higher distances to the contact, the applied stress shifts to a tensile maximum of 0.55±0.12 GPa in the diamond, while compressive stress is slightly reduced to -1.87±0.11 GPa in the ZrN (Fig. S8c).

Concerning the normal stresses in *z*-direction, generally a compressive stress is introduced in both the SL and the NDP, with minima of -1.16±0.13 GPa and -1.25±0.28 GPa, respectively (Fig. S8d). At this load, a slight mismatch between the applied stress distributions in ZrN and the nc diamond can be found, which deviates from Hertz theory and suggests either non-perfect contact due to surface-asperities (Suppl. Note 10) or some plastic deformation very close to the contact not within the resolution of the X-ray experiment. The shear stresses introduced by the indentation are antisymmetric both in vertical and horizontal directions (Fig. S8e). In case of the NDP, the shear stress maxima and minima are slightly lower compared to the SL ZrN thin film, since they counteract the shear stress that are present due to the rotation of the principal stress tensor since before loading.

Finally, the von Mises stress $\sigma_{\mathrm{von\ Mises}}$ magnitudes are presented in Fig. S8f, which reach up to 1.69±0.25 GPa and 3.95±0.18 GPa in the NDP and the SL ZrN, respectively. Please note that the $\sigma_{\mathrm{von\ Mises}}$ distributions have a broadly elliptical shape in the nc diamond (Fig. S8f), while the in the SL ZrN the butterfly-like shape known from a previous experiment is present[20].

Microstructural changes and stress distributions of the SL thin film and the NDP in contact at 0.5 N load

The SAXS micrographs of the SL thin film in contact with the NDP at a load of 0.5 N is presented in Fig. S9a. Despite the now apparent changes in the curvature of the indented SL thin film (Fig. S9a), no significant changes of the SAXS intensities can be found in comparison to the state prior to loading (Fig. S7a).

The azimuthally averaged FWHM of the ZrN 111 and diamond 111 DS rings are shown in Fig. S9b. There, a significant increase of the FWHM in the SL ZrN film can be seen in an almost circular region under the contact with a maximum width of 1.85 µm and a depth of 1.44 µm, where the FWHM is raised above a threshold of 0.65 deg. At this stage of loading, where the maximum $\sigma_{\text{von Mises}}$ is still below the yield stress of ~7 GPa, this region most likely indicates the regions with the highest gradients of stresses of 1st order within the gauge volume[18,19]. Additionally, also in the nc diamond an increase of the FWHM can be seen in the zone closest to the contact, further confirming the high lateral and cross-sectional gradients of stress in the nanomechanical contact.

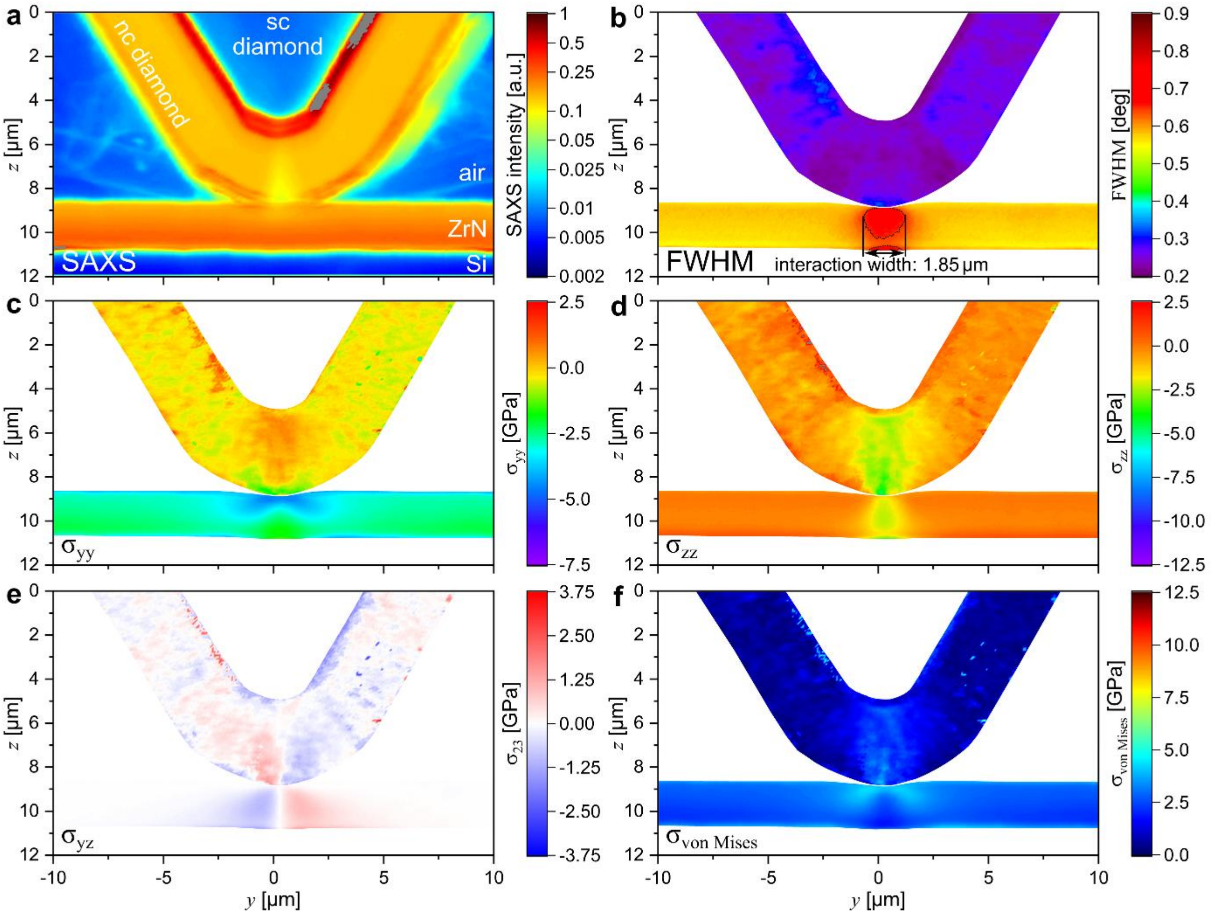

Figure S9. CSnanoXRD data obtained from the interaction of the NDP with the SL film at a load of 0.5 N. In (a) and (b) the SAXSM and the FWHM micrographs are shown, while in (c-e) the σ_{yy}, σ_{yz} and σ_{zz}, are presented, respectively. Finally, in (f) the $\sigma_{\text{von Mises}}$ calculated from the stress components is displayed.

At 0.5 N load, the compressive σ_{yy} distributions introduced in the SL thin film and the diamond close to the contact (Fig. S9c) rise to -4.49±0.16 GPa and -1.53±0.57 GPa, respectively. Even at a load of 0.5 N, the 3 GPa stress level offset resulting from the residual stress in ZrN is still present, indicating exclusively elastic deformation. Furthermore, the resulting tensile stresses at higher distances from the contact increase, reaching 0.72±0.17 GPa in the NDP, while compressive stress is further reduced to -1.38±0.17 GPa in the ZrN (Fig. S9c). Furthermore, additional stress minima are found at the Si/ZrN interface in extension of the butterfly-like compressive stress fields, which reach magnitudes of -3.64 GPa. This stress concentration is not present at 0.2N load (Fig. S8c) and indicates some additional effect of the planar Si/ZrN interface.

The normal stresses in *z*-direction also increase upon raising the load to 0.5 N, the compressive stress rises in both the SL and the nc diamond, with minima of -4.02±0.13 GPa and -3.48±0.15 GPa, respectively (Fig. S9d). Also at this load, a slight mismatch of the applied stress distributions in the SL thin film and the NDP can be found, which deviates from Hertz theory and further suggests a non-perfect contact due to surface-asperities (Suppl. Note 10) or some plastic deformation very close to the contact not within the resolution of the X-ray experiment.

The shear stresses introduced at 0.5 N are intensified compared to the first loading at 0.2 N (compare Fig. S9e and Fig. S9e). The antisymmetric nature both in vertical and horizontal directions is preserved at the higher load (Fig. S9e). Again, in case of the diamond, the shear stress maxima and minima are slightly lower compared to the SL ZrN thin film, for the same reason as before.

Finally, the von Mises stress $\sigma_{von\ Mises}$ magnitudes at 0.5 N load are presented in Fig. S9f, which reach up to 4.18±0.37 GPa and 4.29±0.28 GPa in the NDP and the SL ZrN, respectively. The $\sigma_{von\ Mises}$ distributions now have a noticeable elliptical shape in the nc diamond (Fig. S9f), while the in the SL ZrN the butterfly-like shape is intensified. Additionally, also the $\sigma_{von\ Mises}$ is increased at the Si/ZrN interface in the extension of the butterfly-like shape to ~3.96 GPa. This stress concentration agrees with the increased σ_{yy} presented in in Fig. S9c.

Microstructural changes and stress distributions of the SL thin film and the NDP in contact at 1.0 N load

The SAXS micrographs of the SL ZrN thin film in contact with the nanomechanical probe at a load of 1.0 N is presented in Fig. S10a. In addition to an increased curvature of the indented SL thin film with increasing the load to 1.0 N (Fig. S10a), the SAXS intensity in the indented area is decreased, which indicates a significant compaction of the columnar grained microstructure in comparison to the state prior to loading (Fig. S7a) and the onset of plastic deformation. These findings are confirmed by the FWHM microscopy and stress analysis discussed below.

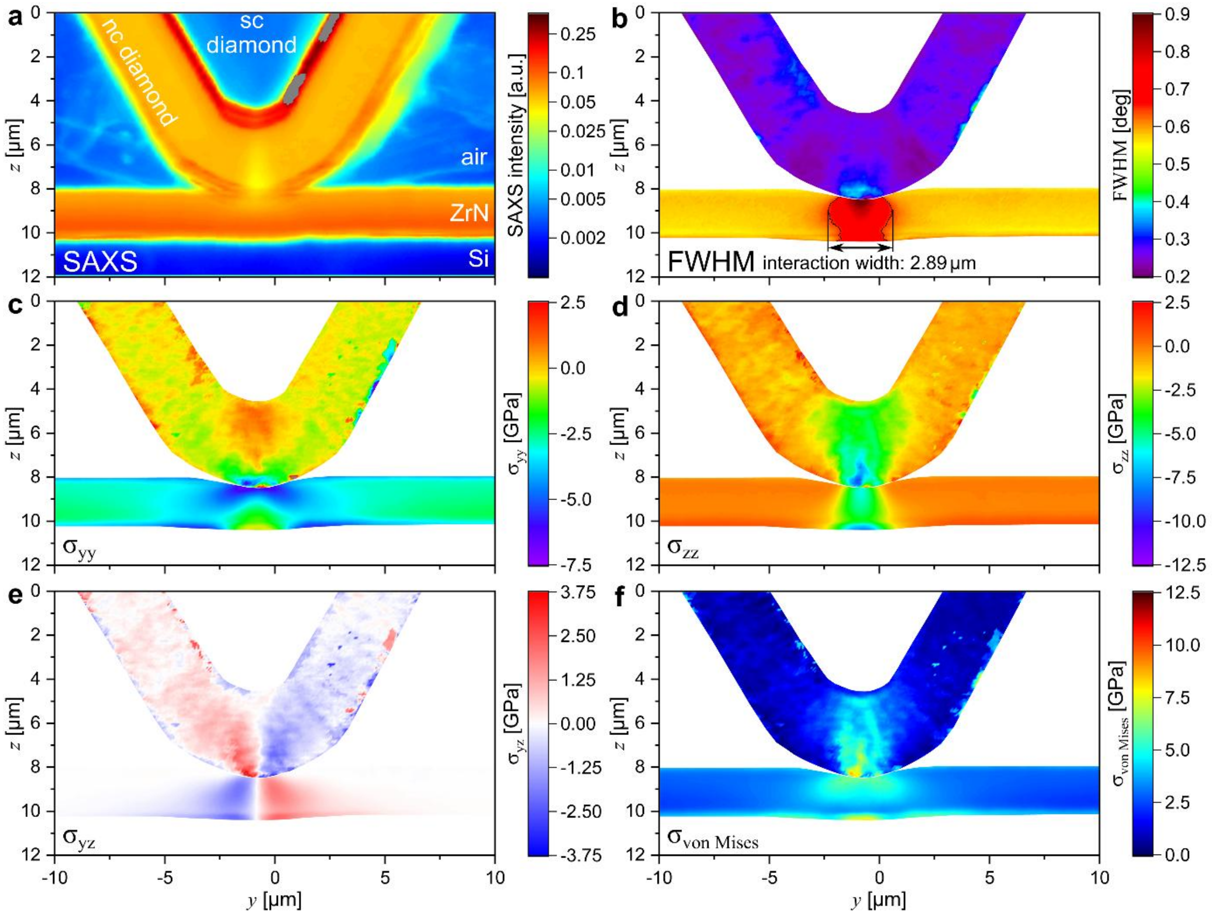

Figure S10. CSnanoXRD data obtained from the interaction of the NDP with the SL film at a load of 1.0 N. In (a) and (b) the SAXSM and the FWHM micrographs are shown, while in (c-e) the σ_{yy}, σ_{yz} and σ_{zz}, are presented, respectively. Finally, in (f) the $\sigma_{\text{von Mises}}$ calculated from the stress components is displayed.

The azimuthally averaged FWHM of the ZrN 111 and diamond 111 DS rings at a load of 1.0 N are shown in Fig. S10b. There, a significant increase of the FWHM in the SL film can be seen in an almost circular region under the contact with a maximum width of 2.89 µm and a depth of 1.91 µm, where the FWHM is raised above a threshold of 0.65 deg. Inside this zone, the FWHM further increases towards the contact and reaches levels close to 0.9 deg, an increase of more than 50% compared to the unloaded state (Fig. S7c). Since the zone of highest FWHM has a comparable outline as the decreased SAXS signal, plastic deformation, intragranular

fracture and grain boundary sliding are highly likely in this zone[18,19]. Again, also in the NDP an increase of the FWHM can be seen in the zone closest to the contact, further confirming the high lateral and cross-sectional gradients of stress close to the contact.

At 1.0 N load, the compressive σ_{yy} distributions directly beneath the contact (Fig. S10c) rise even further to -5.72±0.22 GPa and -4.47±1.24 GPa, respectively. Compared to the load of 0.5 N, the difference of σ_{yy} levels decreased to 1.25 GPa. The inability of the SL to maintain the stress difference of 3 GPa originating from the deposition process confirms that plastic deformation has taken place close to the contact. Furthermore, the resulting tensile stresses at higher distances from the contact increase, which reach 1.02±0.17 GPa in the NDP, while compressive stress is further reduced to -0.90±0.13 GPa in the ZrN (Fig. S10c). Furthermore, the additional stress minima found at the Si/ZrN interface in the extension of the butterfly-like compressive stresses are increased, which reach magnitudes of up to -5.44 GPa.

The normal stresses in *z*-direction also increase further upon raising the load to 1.0 N, the compressive stress rises in both the SL film and the NDP, with minima of -5.62±0.21 GPa and -5.34±1.03 GPa, respectively (Fig. S10d). At this load, the contact stresses are symmetric in the SL thin film and the NDP, indicating a well-established contact between the surfaces (and in turn, a decreased influence of any present surface asperities). Furthermore, σ_{yy} and σ_{zz} in the diamond close to the contact get closer in magnitude, in agreement with Hertzian theory[21,22]. The shear stresses introduced at 1.0 N are further intensified compared to the previous load steps (compare Figs. S8e, S9e and S10e). The antisymmetric nature both in vertical and horizontal directions is preserved at the higher load (Fig. S10e). Again, in case of the nanomechanical probe, the shear stress maxima and minima are slightly lower compared to the SL ZrN thin film, still for the same reasons as before.

Finally, the von Mises stress $\sigma_{von\ Mises}$ magnitudes at 1.0 N load are presented in Fig. S10f, which reach up to 8.89±1.07 GPa and 6.10±0.33 GPa in the NDP and the SL ZrN, respectively. Still, the $\sigma_{von\ Mises}$ distributions have a roughly elliptical shape in the nc diamond (Fig. S10f), while the in the SL ZrN the butterfly-like shape is further intensified. Additionally, the $\sigma_{von\ Mises}$ shows three maxima at the Si/ZrN interface, two of which are located in the extension of the butterfly-like shape, while the third is located directly opposite the contact with a maximum $\sigma_{von\ Mises}$ of 7.93 GPa. This stress concentration is in agreement with the σ_{yy} extrema at the ZrN/Si interface presented in Fig. S10c.

Microstructural changes and residual stress changes of the SL thin film and the NDP after the experiment

The SAXS micrographs of the SL thin film and the NDP after the experiment are presented in Fig. S11a. In addition to the residual curvature after the experiment (Fig. S11a), the SAXS intensity in the indented area has decreased, which indicates a significant compaction of the columnar-grained microstructure in comparison to the state prior to loading (Fig. S7a) and significant plastic deformation and compaction of the thin films' microstructure. These findings are also confirmed by the FWHM microscopy and stress analysis discussed below. Furthermore, the step in the interface (Fig. S11a, *cf*. Fig. 3) that appeared after loading to 2.0 N and the increased intensity between this step and the crack in the Si substrate are still visible, confirming the cracking across the Si/ZrN interface visible at the highest load (Fig. 3) and in the *ex situ* SEM (Figs. S2 and Fig. 2). On the other hand, the NDP appears unchanged compared to the initial state prior to the experiment (compare Figs. S7a and Figs. S11a).

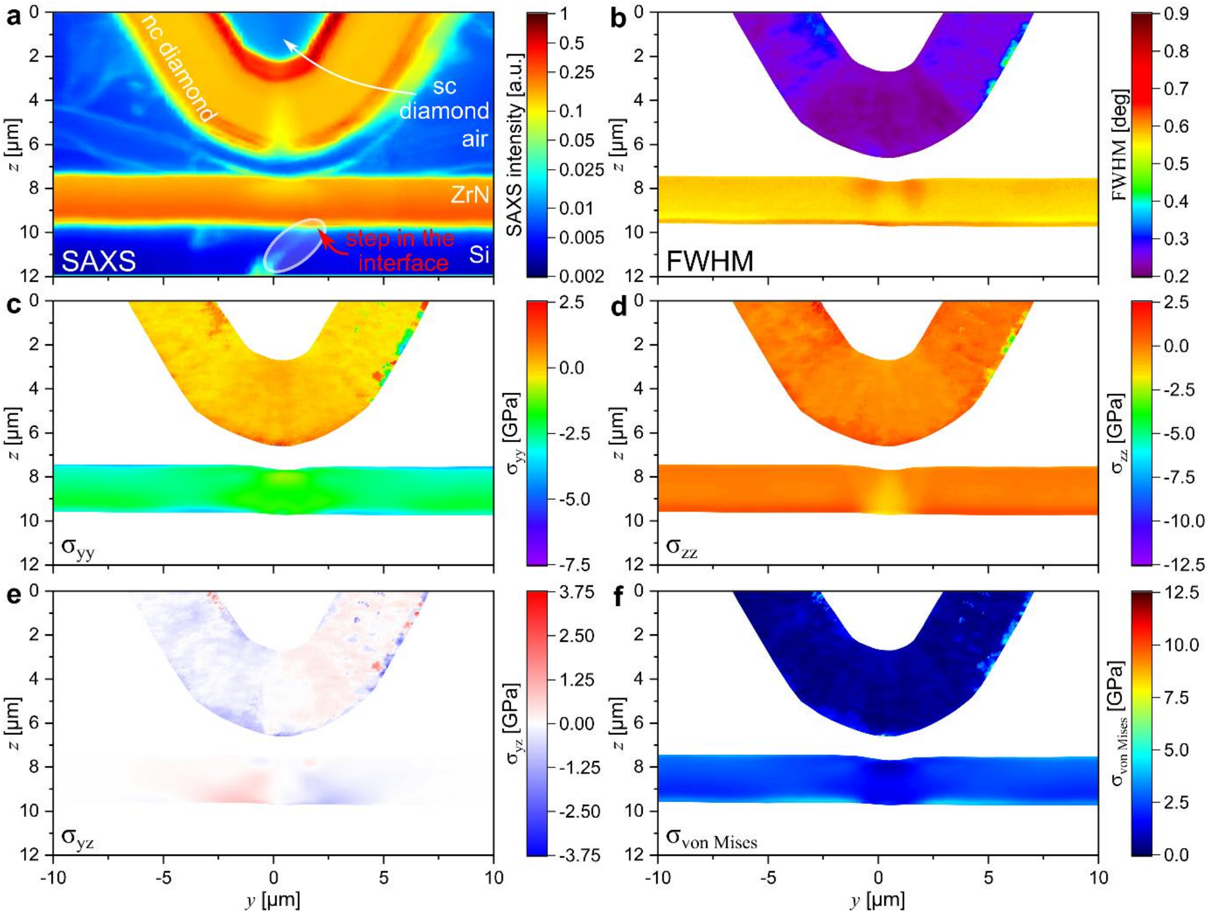

Figure S11. CSnanoXRD data obtained after the interaction of the NDP with the SL film without load. In (a) and (b) the SAXSM and the FWHM micrographs are shown, while in (c-e) the σ_{yy}, σ_{yz} and σ_{zz}, are presented, respectively. Finally, in (f) the $\sigma_{\mathrm{von\ Mises}}$ calculated from the stress components is displayed.

The azimuthally averaged FWHM of the ZrN 111 and diamond 111 DS rings after the experiment are shown in Fig. S11b. There, a significant increase of the FWHM in the SL ZrN

film can be seen in the typical butterfly-like outline where the σ_{yy} stress distributions had been most intense during loading (Fig. S7c, S8c, S9c, Fig. 3a). Since the zone of highest FWHM also has a similar outline compared to the decreased SAXS signal, the high multiaxial compressive stress during loading has likely enabled plastic deformation, intragranular fracture and grain boundary sliding in this zone[18,19]. Again, the NDP appears unchanged by the indentation experiment, the average FWHM of the nc diamond 111 DS ring appears unchanged (Fig. S11b) compared to the initial state (Fig. S7b) prior to the experiment.

The stress distributions presented in Fig. S11c-f show the changes in the SL ZrN thin film due to plastic deformation close to the contact and fracture close to the interfaces, as well as the elastic response of the surrounding material. Most notable, the $\sigma_{von\ Mises}$ is close to zero immediately adjacent to the contact region, which indicates a hydrostatic compressive stress state in this zone with no deviatoric contributions. Furthermore, the zones of increased von Mises stress close to the Si/ZrN interface remain, which indicates significant interface shear. Again, the stress distributions in the NDP remain unchanged by the experiment (compare Figs. S7c-f to Fig. S11c-f).

Supplementary Note 4: Detailed stress analysis for ML thin film

Microstructure and residual stress of the ML thin film and the NDP before loading

The experimental microstructure and stress data of the ML thin film before contact with the NDP are presented in Fig. S12. In the phase plot shown in Fig. S12a, where the intensities are integrated azimuthally over 360 deg (Fig. 1), exclusively peaks corresponding to the cubic B1 ZrN phase[23] can be detected. Additionally, a raised background can be seen at the positions of the amorphous (a-)ZrCu metallic glass interlayers. The azimuthal intensity distributions of the ZrN 111 Debye-Scherrer (DS) ring are presented in Fig. S12b and reveal a distinct change of the azimuthal orientation of the 111 DS ring between the 1st and 2nd ZrN sublayers. In the layer grown on Si, a 111 fibre texture is evidenced by azimuthal intensity maxima at ±90 deg. On the contrary, in the 2nd to 4th ZrN layer grown on the individual a-ZrCu interlayer, azimuthal intensity maxima are present at ±55 and ±125 deg, in agreement with a 200 fibre texture[4,8].

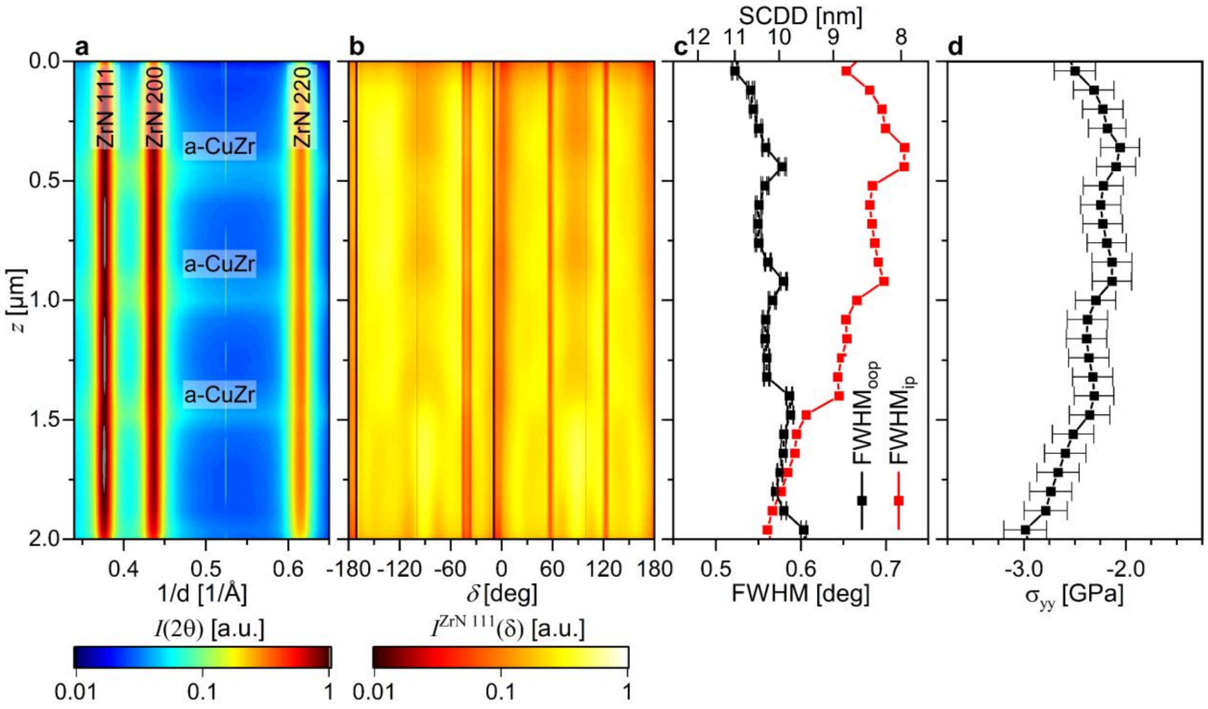

Figure S12. CSnanoXRD data retrieved from the ML thin film before the experiment. In (a) and (b) the phase plot depicting the ZrN 111, 200 and 220 reflections as well as an increased background at the positions of the a-ZrCu interlayers and the azimuthal distribution of the diffracted intensities of the ZrN 111 reflections are presented, respectively. The dark vertical intersections in (b) are due to the gaps between the individual segments of the Eiger 4 M detector. The cross-sectional FWHM variation is shown in (c), while the residual stress evaluated from the ZrN 111 DS ring is presented in (d).

The FWHM was evaluated along the in-plane (*y*-direction in Fig. 1) and out-of-plane (*z*-direction in Fig. 1) directions to get qualitative insight into the morphology of the ML ZrN-ZrCu thin film. The cross-sectional evolution is typical in the out-of-plane direction, since the out-of-plane FWHM is decreasing in every ZrN sublayer (Fig. S12c). On the other hand, the FWHM in the in-plane orientation gradually increases (Fig. S12c). However, this could be

related to the change of texture, since in this case a combination of 200 fibre texture and elongated grains is present, which limits the size of coherently diffracting domains (SCDD) along the ⟨111⟩ directions. Finally, the cross-sectional evolution of the in-plane residual stress is presented in Fig. S12d and shows a decreasing residual stress magnitude ranging from -2.99±0.219 GPa to -2.05±0.19 GPa at the Si/ZrN interface to the topmost ZrCu/ZrN interface. Above that, in the topmost ZrN sublayer, the residual stress magnitude gradually increases again and reaches a value of -2.50±0.20 GPa at the films surface. The gradual evolution of residual stress is in agreement with the FWHM and texture development of the film (Fig. S12).

The SAXS micrographs of the ML thin film and the nanomechanical probe are presented in Fig. S13a. Of course, the diamond probe has a similar appearance in the SAXS micrograph in Fig. S13a compared to the one in Fig. S7a, since it was only deformed elastically during both experiments (*cf.* Fig. 6 and Suppl. Note 5). Furthermore, the ZrCu interlayers are visible by an increase of the SAXS signal (Fig. S13a), which corresponds well with the increase of variations of electron density in the amorphous layer[24] and the renucleation of smaller columnar grains in the adjacent ZrN sublayer[4,8].

The FWHM micrograph for the state before loading of the ML thin films is presented in Fig. S13b, where the FWHM of the nc diamond is again lower directly at the indenter tip, with an opening angle of 90 deg enclosing the area of decreased FWHM (see also Suppl. Notes 3 and 10). In the case of ZrN, the periodic increase of the FWHM corresponds to the renucleation of columnar grains in the ZrN sublayers after deposition of the amorphous ZrCu interlayers[8]. The average of the azimuthally averaged FWHM in the ZrN-ZrCu thin film before the *in situ* experiment was 0.577±0.021 deg. Despite the average FWHM being slightly higher compared to the SL ZrN thin film (Suppl. Note 3), a boundary FWHM value of >0.65 deg should remain a valid indicator to describe zones of highest deformation, since it is separated from the mean value by at 3.5 times the standard deviation of the averaged FWHM.

Again, the nc diamond forming the NDP was essentially free of residual stress before contact, as can be seen from the 2D contour plots of the σ_{yy} and σ_{zz} stress components presented in Fig. S13c and d, respectively. In addition, the shear stress σ_{yz} showed a separation in positive and negative shear, which indicates that the principal stress tensor is oriented parallel to the surface normal of the NDP. Contrary to this, the ML thin film exhibits relatively large in-plane residual stress (Fig. S13c) with a distribution similar to the one outlined in Fig. S12d and an average value of -2.40±0.25 GPa. Furthermore, a slight gradient of the σ_{zz} stress component ranging from 0.47±0.25 GPa to 0.01±0.20 GPa at the Si/ZrN interface and the film surface,

respectively. This is interpreted as the gradual development of d_0, which was not corrected in the evaluation (*cf.* Methods) However, the σ_{zz} magnitudes are low compared to the applied stresses and do not impair the conclusions drawn in this work. The σ_{yz} magnitudes evaluated from the ZrN thin film are slightly negative close to 0 (Fig. S13e), which is a consequence of the slight misalignment of the film (Fig. S13). This further indicates an exclusive presence of in-plane residual stress.

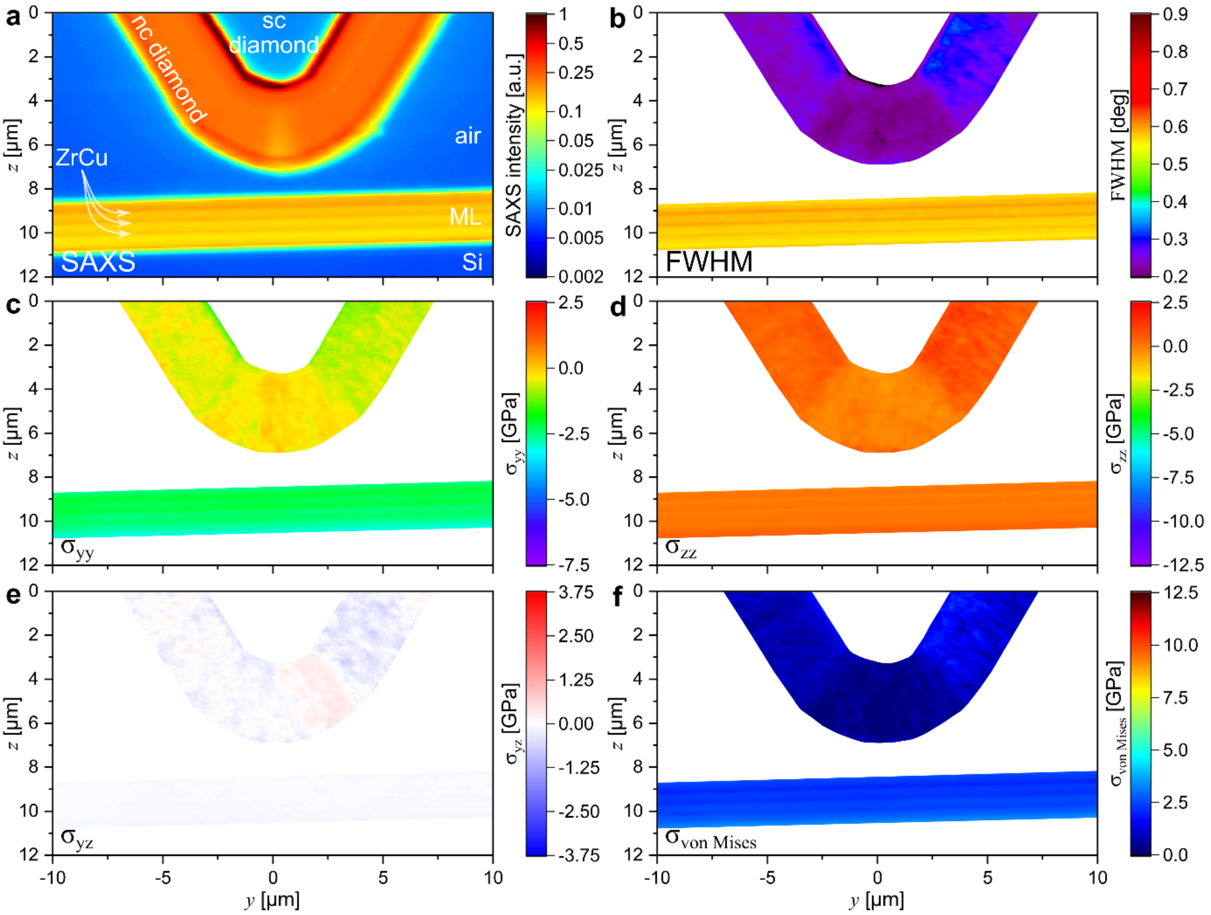

Figure S13. CSnanoXRD data obtained from the NDP and the ML film before loading. In (a) and (b) the SAXSM and the FWHM micrographs are shown, while in (c-e) the σ_{yy}, σ_{yz} and σ_{zz}, are presented, respectively. Finally, in (f) the $\sigma_{\text{von Mises}}$ calculated from the stress components is displayed.

Finally, the von Mises stress $\sigma_{\text{von Mises}}$ magnitudes are presented in Fig. S13f, which are close to 0 and centered around the residual stress values discussed above in the case of the nc diamond thin film and the ML thin film, respectively.

Microstructural changes and stress distributions of the ML thin film and the NDP in contact at 0.2 N load

The SAXS micrographs of the ML thin film in contact with the NDP at a load of 0.2 N are presented in Fig. S14a. Despite the NDP being in contact with the thin film, no changes of the SAXS intensities can be found in comparison to the state prior to loading (Fig. S13a). The horizontal streaks at the Si/ML interface are most probably related to some minor misalignment between the tip and the ML thin film, which results a slight, mechanically negligible, rotation which in turn triggers some X-ray reflectivity[25,26].

The azimuthally averaged FWHM of the ZrN 111 and diamond 111 DS rings are shown in Fig. S14b. There, a distinct increase of the FWHM in the ML film can be seen in a mushroom-shaped region under the contact with a maximum width of 0.95 μm of the 0.65 deg contour. This is in contrast to the behavior of the SL thin film at the same load (Fig. S8b). No apparent changes are visible in the nc diamond of the indenter tip (Fig. S14b). Since the applied stresses are not yet high enough to induce plastic deformation in the ZrN (a hardness of 21 GPa (Fig. 2) equals a yield stress of 7 GPa considering a Tabor factor of 3[16,17]), the increase of the FWHM can therefore be related exclusively to gradients of stresses of 1st order in the X-ray gauge volume[18,19] and thus already indicates the zones of highest deformation. However, the amorphous ZrCu layer is considerably weaker, with a yield stress of ~1.9 GPa[27]. Thus, plastic deformation in the ZrCu can already occur at this lowest load and trigger a corresponding response in the ZrN, which translates to the considerably higher FWHM found in ZrN in the ML thin film (Fig. S14b) compared to the SL ZrN thin film (Fig. S8b) at the same load.

At 0.2 N load, near equal compressive σ_{yy} distributions are introduced in the ML thin film and the NDP close to the contact (Fig. S14c, compare also with Fig. 6d in the manuscript), which rise σ_{yy} to -3.37±0.19 GPa and -0.82±0.31 GPa, respectively. In case of ZrN, the introduced compressive stress is in addition to the already present residual stress of ~ -2.70 GPa (Fig. S12). Furthermore, at higher distances to the contact, the applied stress shifts to a tensile maximum of 0.05±0.16 GPa in the NDP, while compressive stress at the ZrN/Si interface remains nearly unchanged at a level of -2.70±0.19 GPa (Fig. S14c, Fig. 6d).

Concerning the normal stresses in *z*-direction, generally a compressive stress is introduced in both the ML and the NDP, with values of -0.76±0.18 GPa and -0.78±0.28 GPa, respectively (Fig. S8d) directly at the contact. At this load, the maximum σ_{zz} of ~ -3.93±0.26 GPa in the ML thin film is found at a depth of ~0.5 μm. This distribution deviates from the Hertz theory[21,22] (see Suppl. Note 6 for details) and suggests some plastic deformation within the amorphous ZrCu and a corresponding elastic response in the ZrN. The shear stresses introduced by the indentation are antisymmetric both in vertical and horizontal directions (Fig. S14e). In case of

the diamond, the shear stress maxima and minima are slightly lower compared to the ML thin film, since they counteract the shear stress that are present due to the rotation of the principal stress tensor since before loading (Fig. S14e). Additionally, periodic modulations of the shear stress can already be seen in the stressed zones inside the ML thin film, which indicate lower shear stress close to the amorphous ZrCu interlayer (Fig. S14e).

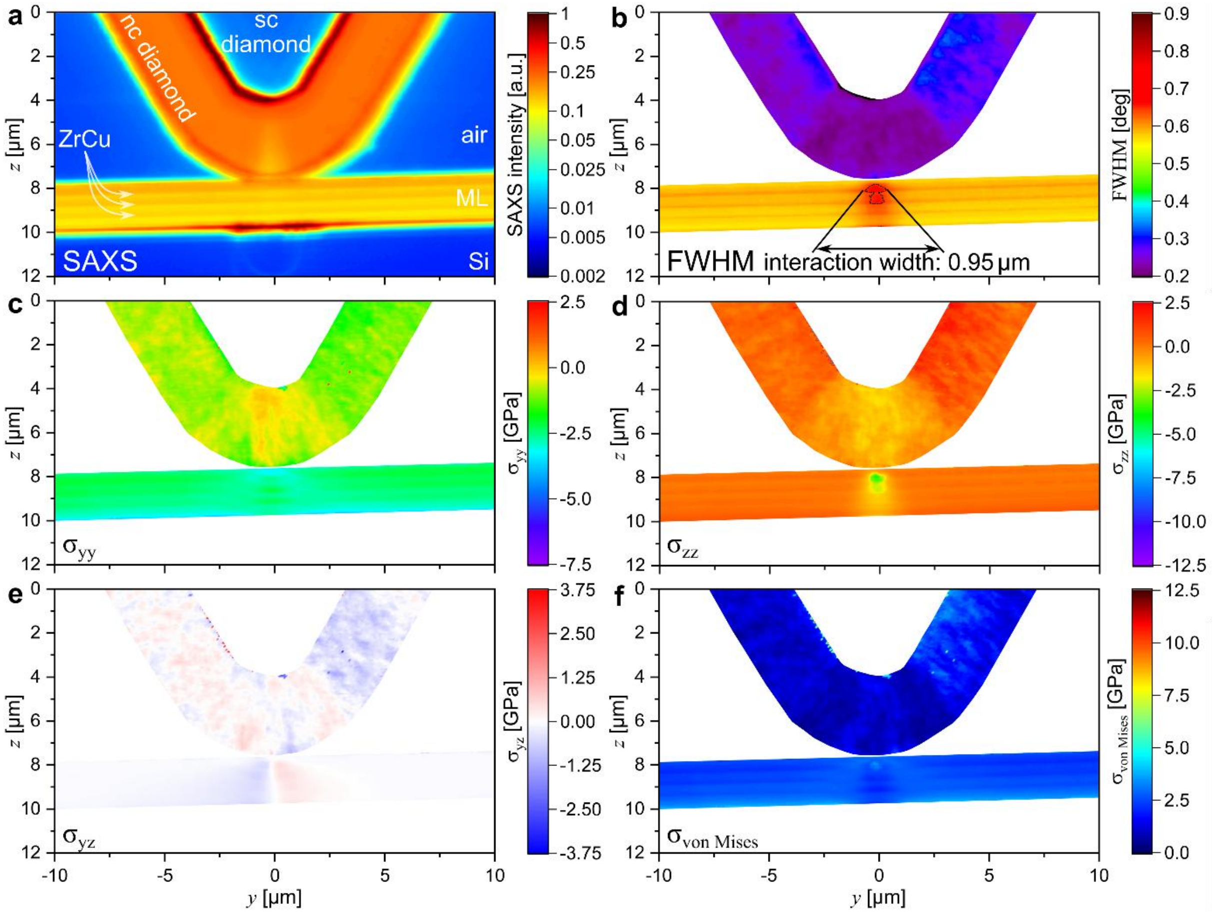

Figure S14. CSnanoXRD data obtained from the interaction of the NDP with the ML film at a load of 0.2 N. In (a) and (b) the SAXSM and the FWHM micrographs are shown, while in (c-e) the σ_{yy}, σ_{yz} and σ_{zz}, are presented, respectively. Finally, in (f) the $\sigma_{von\ Mises}$ calculated from the stress components is displayed.

Finally, the von Mises stress $\sigma_{von\ Mises}$ magnitudes are presented in Fig. S14f, which reach up to 1.86±0.24 GPa and 3.55±0.25 GPa in the NDP and the ML thin film, respectively. Please note that the $\sigma_{von\ Mises}$ distributions have no discernable shape in the nc diamond (Fig. S8f), while the in the SL ZrN the butterfly-like shape known from a previous experiment is present[20] next to a circular zone, probably introduced by the highest vertical stress component σ_{zz} (Fig S13d). The formation of two zones of elevated $\sigma_{von\ Mises}$ deviates from classical elastic models[21,22] (still valid for the SL thin film at the same load, Fig. S8f) and is a further indication of onset of plastic deformation in the amorphous ZrCu.

Microstructural changes and stress distributions of the ML thin film and the NDP in contact at 0.5 N load

The SAXS micrographs of the ML thin film in contact with the NDP at a load of 0.5 N are presented in Fig. S15a. Despite the now apparent changes in the curvature of the indented ML thin film (Fig. S15a), no significant changes of the SAXS intensities can be found in comparison to the state prior to loading (Fig. S13a). Again, the horizontal streaks at the Si/ML interface are most probably related to some minor misalignment between the tip and the ML thin film, which results a slight, mechanically negligible, rotation, which in turn triggers some X-ray reflectivity[25,26].

The azimuthally averaged FWHM of the ZrN 111 and diamond 111 DS rings are shown in Fig. S15b. There, a significant increase of the FWHM to values above 0.65 deg in the ML film can be seen in an almost rectangular region under the contact with a maximum width of 1.93 µm stretching over the entire thickness of the thin film. This is in sharp contrast to the deformation of the SL thin film (Fig. S9b), where such a zone of highest deformation was much less developed at a load of 0.5 N and not yet present at a load of 0.2 N.

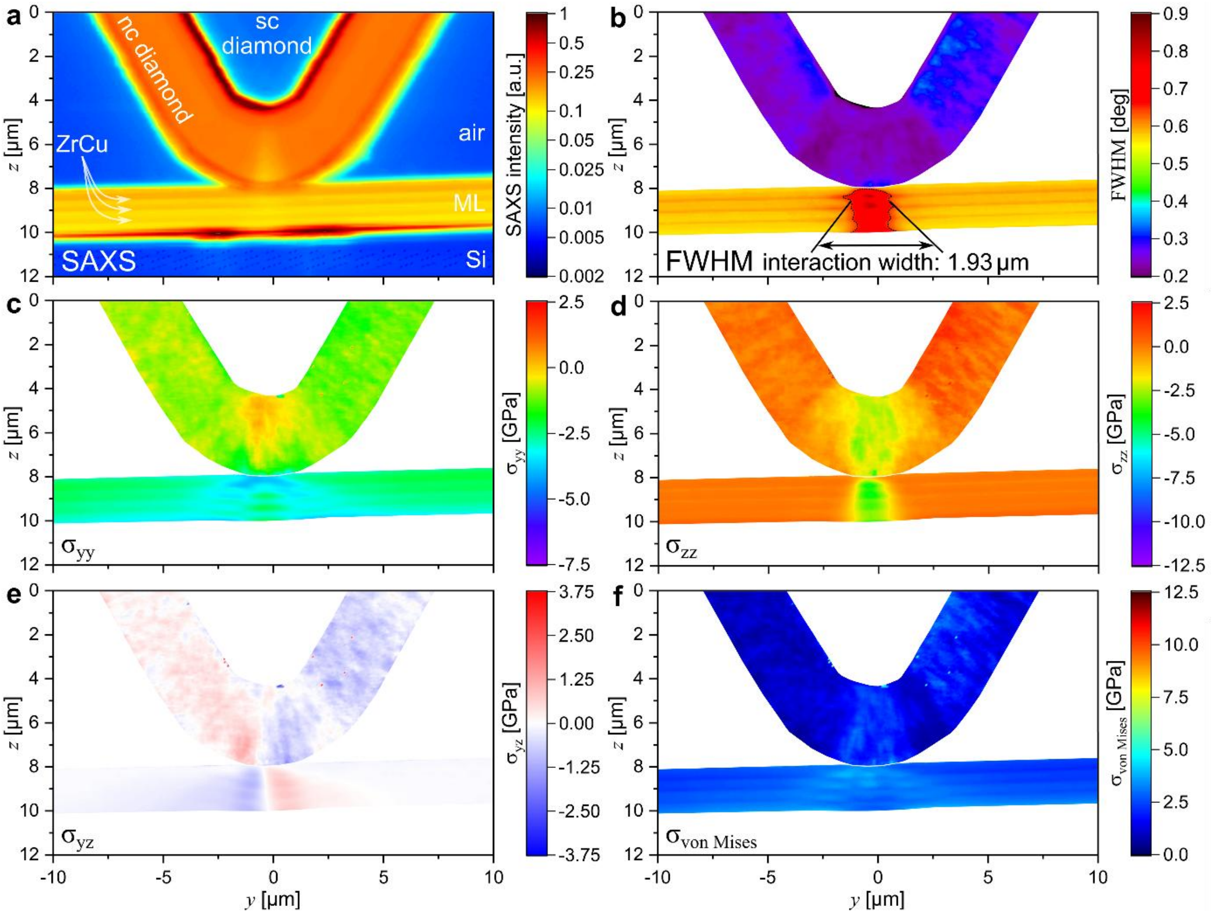

Figure S15. CSnanoXRD data obtained from the interaction of the NDP with the ML film at a load of 0.5 N. In (a) and (b) the SAXSM and the FWHM micrographs are shown, while in (c-e) the σ_{yy}, σ_{yz} and σ_{zz}, are presented, respectively. Finally, in (f) the $\sigma_{von\ Mises}$ calculated from the stress components is displayed.

Inside the zone of highest deformation, maximum FWHM values close to 0.9 deg are found in the two ZrN layers closest to the surface, with a distinct concentration of lower FWHM close to the ZrN/ZrCu interfaces (Fig. S15b). This can indicate that close to the ZrCu layers the gradients of stresses of 1st order are less developed and furthermore might indicate a shielding from the highest stress.

At 0.5 N load, the compressive σ_{yy} distributions introduced in the ML thin film and the NDP close to the contact (Fig. S15c) rise to -3.86±0.22 GPa and -2.27±0.41 GPa, respectively. This stress difference is significantly lower than the average residual stress of -2.42±0.29 GPa and signals plastic deformation in the ML thin film. Additionally, the resulting tensile stresses at higher distances from the contact increase, reaching 0.76±0.12 GPa in the NDP, while compressive stress is slightly reduced to -1.90±0.19 GPa in the ZrN (Fig. S15c). Furthermore, progressing outward from the zones of highest σ_{yy} magnitudes close to the contact, a periodic decrease and increase of σ_{yy} can be seen in Fig. S15c, which indicates that outside of the central zone with high vertical compression (Fig. S15d) the amorphous ZrCu in the ML film cannot accumulate further in-plane stress and this is in turn is detected in the adjacent ZrN areas accessible to the X-ray stress analysis. Furthermore, additional stress minima are found at the Si/ZrN interface in the extension of the butterfly-like compressive stress fields, reaching magnitudes of -3.65 GPa. This stress concentration is not present at 0.2N load (Fig. S14c) and indicates some additional effect of the planar Si/ZrN interface also found in the ZrN SL thin film at 0.5 N load (Fig. S9c). This may be attributed to a bending-like contribution to the stress state, since the Si substrate has a lower Young's modulus (E_{Si} = 155 GPa[28]) compared to the ML thin film (Fig. 2). Exemplarily, three-point bending of a clamped cantilever introduces large compressive stresses in the bottom half of the cantilever close to the clamping[20].

The normal stresses in *z*-direction also increase upon raising the load to 0.5 N, the compressive stress rises in both the ML thin film and the nc diamond, with stresses of -1.57±0.22 GPa and -1.65±0.44 GPa at the contact, respectively (Fig. S15d). At this load, maxima σ_{zz} of ~ -4.26±0.23 and -4.72±0,19 GPa in the ZrN-ZrCu thin film are found at a depth of ~0.3 μm and at the ZrN/Si interface. The appearance of a secondary maximum close to the interface indicates that the ZrN/Si interface acts as a stress concentrator during loading.

The shear stresses introduced at 0.5 N are intensified compared to the first loading at 0.2 N (compare Fig. S14e and Fig. S15e). In case of the NDP, the shear stress maxima and minima are slightly lower compared to the ML thin film, for the same reason as before (Fig. S15e). Additionally, periodic modulations of the shear stress can already be seen in the stressed zones inside the ML thin film, which indicate lower shear stress close to the amorphous ZrCu

interlayer (Fig. S15e). Since the shear stress indicates the rotation of the principal stress tensor, one can see from the reduced shear stress magnitudes (Fig. S15e) that the σ_{yy} is the minimum principal stress in these regions ($\sigma_{yy} < \sigma_{zz}$)[29]. Thus the critical shear stress (as a yield criterion[29]) applied to zones of reduced σ_{yy} and σ_{yz} magnitudes would yield $\sim\frac{1}{2}\sigma_{yy} \approx$ -1.6 GPa, which is in good agreement with the yield stress of 1.8 GPa determined from microcantilever bending experiments[27].

Finally, the von Mises stress $\sigma_{von\ Mises}$ magnitudes at 0.5 N load are presented in Fig. S15f, which reach up to 3.20±0.28 GPa and 3.80±0.29 GPa in the NDP and the SL ZrN, respectively. The $\sigma_{von\ Mises}$ distributions have a roughly elliptical shape in the nc diamond (Fig. S15f), similar as in the SL ZrN thin film (Fig. S9f). The butterfly-like shape known from a previous experiment is present[20] and is intensified in case of the ML ZrN-ZrCu thin film. Additionally, the previously visible circular zone beneath the contact, probably introduced by the highest vertical stress component (Fig. S15d) is still present in the center of the contact. Furthermore, the von Mises stress magnitudes are periodically reduced close to the amorphous ZrCu interlayer, further highlighting that its elastic limit (Fig. S15f) is crucially underpinning the changes of the local mechanical behavior of the ML thin film compared to the SL film.

In summary, while the NDP shows a similar behavior in contact with the SL and ML thin films (compare Fig. S15 and Fig. S9) at 0.5 N load, the stress response of the ML thin film clearly deviates significantly from the SL thin film. This indicates the strong influence of the amorphous ZrCu interlayers on the mechanical behavior of the ML thin film, effectively resulting in an earlier onset of localized plastic deformation and a redistribution of stress maxima within the thin film.

Microstructural changes and stress distributions of the ML thin film and the NDP in contact at 1.0 N load

The SAXS micrographs of the ML thin film in contact with the NDP at a load of 1.0 N are presented in Fig. S16a. Despite the further increase of the curvature of the indented MLthin film (Fig. S16a) and the still-present reflectivity phenomena discussed above, no significant changes of the SAXS intensities can be found in comparison to the state prior to loading (Fig. S13a).

The azimuthally averaged FWHM of the ZrN 111 and diamond 111 DS rings are shown in Fig. S16b. There, the almost rectangular zone where the FWHM is raised above a threshold of 0.65 deg, stretching over the entire film thickness with a maximum width of ~3.48 µm, can be seen in the ML thin film. Additionally, 4 elliptical regions with FWHM values close to and above 0.9 deg are visible under the center of the contact (Fig. S16b). The separation of these ellipses at the positions of the ZrCu interlayers indicates that close to the ZrCu layers the gradients of stresses of 1st order are less developed and furthermore indicate a shielding of the adjacent ZrN from the highest stress at these positions.

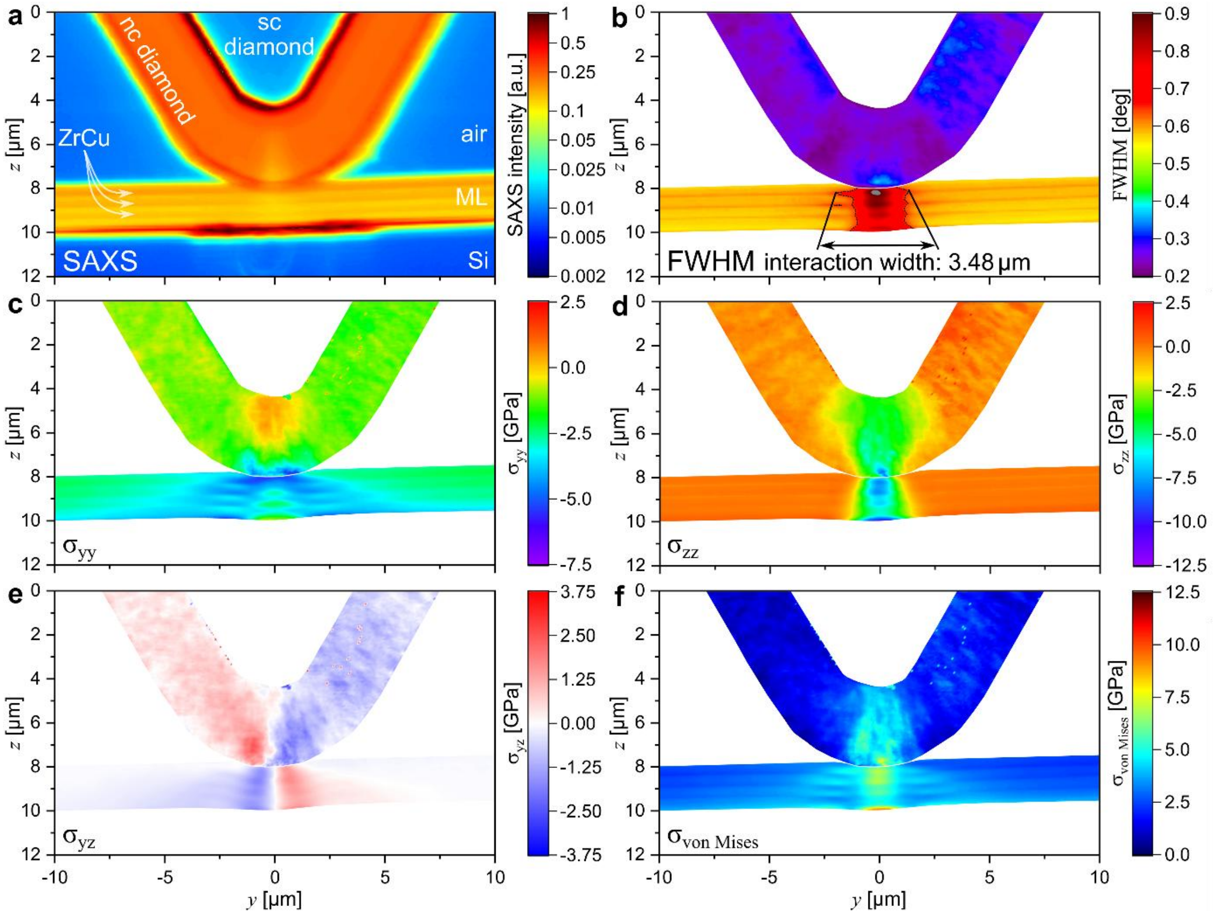

Figure S16. CSnanoXRD data obtained from the interaction of the NDP with the ML film at a load of 1.0 N. In (a) and (b) the SAXSM and the FWHM micrographs are shown, while in (c-e) the σ_{yy}, σ_{yz} and σ_{zz}, are presented, respectively. Finally, in (f) the $\sigma_{\mathrm{von\ Mises}}$ calculated from the stress components is displayed.

An elevated FWHM is also visible in the NDP close to the contact, indicating a further increase of the gradients of stresses of 1st order within the X-ray gauge volume in the nc diamond (Fig. S16b). Again, the size and shape of the high-deformation zone in the ML thin film is in sharp contrast to the deformation of the SL thin film (Fig. S10b), where the zone of highest deformation retains an elliptic shape and extends only to a width of 2.89 μm at a load of 1.0 N. The compressive σ_{yy} distributions introduced at a load of 1.0 N in the ML thin film and the NDP close to the contact (Fig. S16c) rise to -5.37±0.22 GPa and -3.50±0.79 GPa, respectively. This stress difference is further decreased compared to the one observed at 0.5 N load and thus signals plastic deformation in the ML ZrN-ZrCu thin film. Additionally, the resulting tensile stresses at higher distances from the contact increase, which reach 1.00±0.17 GPa in the NDP, while compressive stress is further reduced to -1.23±0.18 GPa in the ZrN (Fig. S16c). Additionally, the periodic increase and decrease of σ_{yy} along the extension of its highest magnitudes close to the contact is pronounced even further (Fig. S16c) in comparison with the load of 0.5 N. This confirms that outside of the zone of high vertical compression (Fig. S16d) the amorphous ZrCu in the ML film cannot accumulate further in-plane stress, which is then seen in the adjacent ZrN areas accessible to the X-ray stress analysis. Furthermore, additional stress minima are found at the Si/ZrN interface in the extension of the butterfly-shaped compressive stress distributions, reaching magnitudes of -5.46 GPa and indicating some additional effect of the planar Si/ZrN interface also found in the SL thin film at 1.0 N load (Fig. S10c).

The normal stresses in *z*-direction also increase upon raising the load to 1.0 N, the compressive stress rises in both the ML and the nc diamond, with stresses of -6.61±0.23 GPa and -5.36±0.90 GPa at the contact, respectively (Fig. S16d). At this load, maxima σ_{zz} of ~ -8.10±0.24 and -8.77±0.18 GPa in the ZrN-ZrCu thin film are found at a depth of ~0.15 μm and at the ZrN/Si interface. The increase of the secondary σ_{zz} maximum close to the interface further confirms that the ZrN/Si interface acts as a stress concentrator during loading with the NDP, while the shift of the primary maximum towards the contact indicates that the elastic limit of ZrN is also reached at 1.0 N load.

The shear stresses introduced at 1.0 N are intensified compared to the first and second load steps at 0.2 and 0.5 N, respectively (compare Figs. S14e, S15e and S16e). Additionally, periodic modulations of the shear stress can still be seen in the stressed zones, which indicate lower shear stress close to the amorphous ZrCu interlayer (Fig. S16e). Again, since the shear stress indicates the rotation of the principal stress tensor, one can see from the reduced shear stress magnitudes (Fig. 15e) that the σ_{yy} is the minimum principal stress in these regions (σ_{yy} <

σ_{zz})[29]. Thus the critical shear stress yield criterion[29] applied to zones of reduced σ_{yy} and σ_{yz} magnitudes would yield ~$\frac{1}{2}\sigma_{yy}$ ≈ -1.6-1.85 GPa, in very good agreement with the yield stress of 1.8 GPa determined from microcantilever bending experiments[27].

Finally, the von Mises stress $\sigma_{von\ Mises}$ magnitudes at 0.5 N load are presented in Fig. S16f, reaching up to 7.39±0.48 GPa and 9.59±0.29 GPa in the NDP and the SL thin film, respectively. While the highest $\sigma_{von\ Mises}$ in the nc diamond is found close to the contact, the maximum stress in the ML thin film is accumulated at the ZrN/Si interface (Fig. S16f). Again, the $\sigma_{von\ Mises}$ distributions have an elliptical shape in the nc diamond (Fig. S16f), similar as in the SL thin film (Fig. S10f). Contrary to the previous load steps, the butterfly-like shape known from a previous experiment[20] is not anymore the most prominent feature in Fig. S16f, but the highest stresses are accumulated in 3 ellipsoid zones beneath the contact and at the ZrN/Si interface. These are also the zones of highest σ_{zz}, showcasing that the deviatoric component of the stress tensor is governed by a single stress component inside the immediate contact zone. Furthermore, the von Mises stress magnitudes are periodically reduced close to the amorphous ZrCu interlayer inside and outside the contact zone, further highlighting that its elastic limit (Fig. S16f) crucially determines the changes of the local mechanical behavior of the thin film.

Again, while the NDP shows a similar behavior in contact with the SL and ML thin films (compare Fig. S14-16 and Fig. S8-10) at loads of 0.2, 0.5 1.0 N, the stress response of the ML thin film deviates increasingly from the SL thin film with increasing load. This indicates the crucial influence of the amorphous ZrCu interlayers on the mechanical behavior of the ML thin film, which leads to an earlier onset of localized plastic deformation and a redistribution of stress maxima within the thin film.

Microstructural changes and residual stress changes of the ML thin film and the NDP after the experiment

The SAXS micrographs of the ML thin film and the NDP after the experiment are presented in Fig. S17a. In addition to the residual curvature after the experiment (Fig. S17a), the SAXS intensity under the indented area has slightly decreased, which indicates a compaction of the columnar grained microstructure in comparison to the state prior to loading (Fig. S13a) and significant plastic deformation and compaction of the thin film's microstructure. Additionally, an increase in the scattered intensity can be seen at the edges of the former contact in agreement with the cracks found by SEM after the experiment (Suppl. Note 1, Fig. 2). These findings are confirmed by the FWHM microscopy and stress analysis discussed below. On the other hand, the NDP appears unchanged compared to the initial state prior to the experiment (compare Figs. S13a and Figs. S17a).

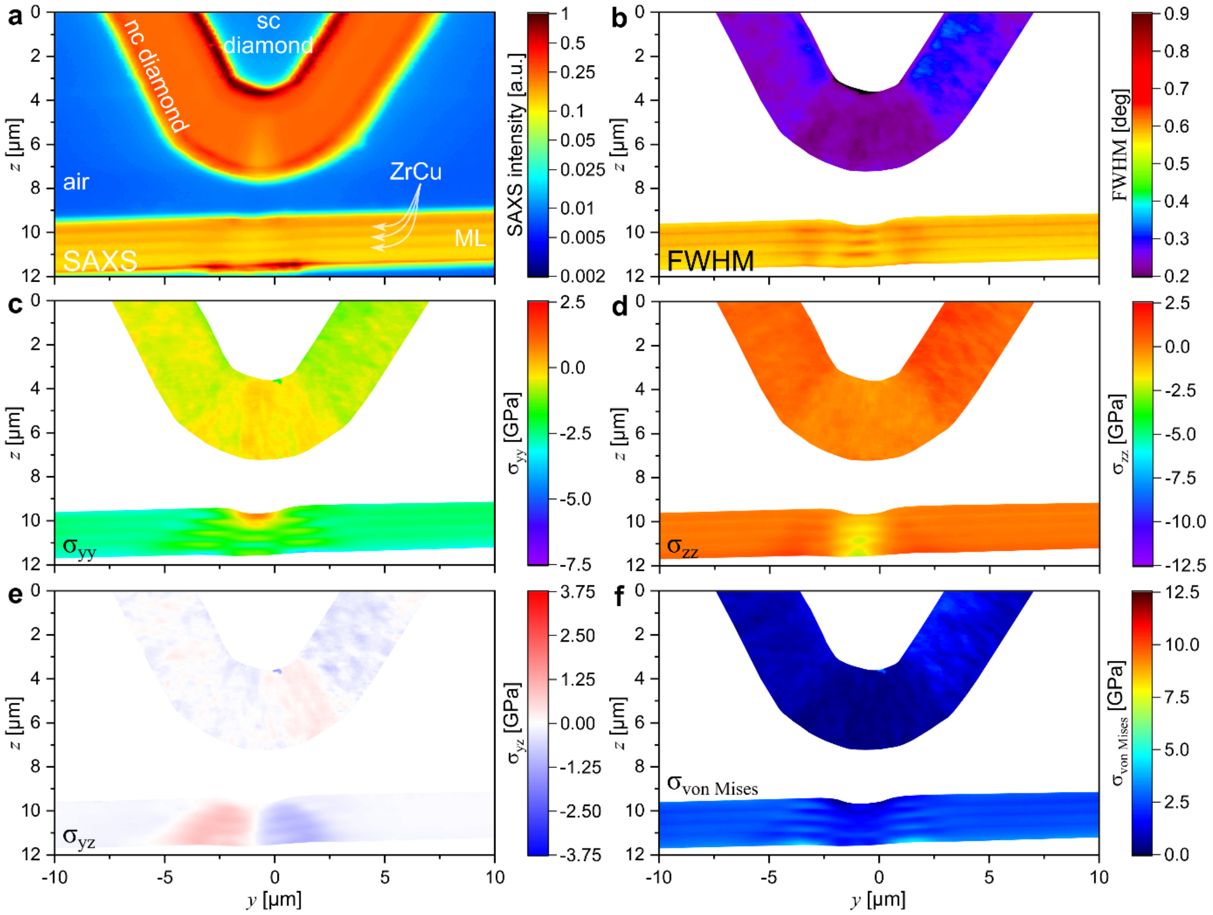

Figure S17. CSnanoXRD data obtained after the interaction of the NDP with the ML film without applied load. In (a) and (b) the SAXSM and the FWHM micrographs are shown, while in (c-e) the σ_{yy}, σ_{yz} and σ_{zz}, are presented, respectively. Finally, in (f) the $\sigma_{von\ Mises}$ calculated from the stress components is displayed.

The azimuthally averaged FWHM of the ZrN 111 and diamond 111 DS rings after the experiment are shown in Fig. S17b. There, a significant increase of the FWHM in the ML thin film can be seen outside the former contact zone, while along the projected surface normal of

the edges of the curved part the FWHM is reduced. Inside the former contact volume that was compressed the most during loading (Fig. 3), the cross-sectional periodic variation is increased compared to the initial state (Fig. S17b vs. Fig. S13b), which indicates narrow repeating ranges of compaction, intragranular fracture and grain boundary sliding in this zone[18,19]. Again, the NDP appears unchanged by the indentation experiment, the average FWHM of the nc diamond 111 DS ring appears unchanged (Fig. S17b) compared to the initial state (Fig. S13b) prior to the experiment.

The stress distributions presented in Fig. S17c-f represent the changes in the ML thin film due to plastic deformation close to the contact and fracture close to the interfaces, as well as the elastic response of the surrounding material. As can be seen from Fig. S17c-f, the residual stress levels in the ML thin film are significantly higher compared to data obtained from the SL thin film presented in Fig. S13c-f. Most notable, the $\sigma_{von\ Mises}$ is close to zero immediately surrounding the prior contact region, which indicates a hydrostatic compressive stress state in this zone with no deviatoric contributions. Furthermore, the zones of increased von Mises stress close to the ZrN/ZrCu and Si/ZrN interfaces remain, which indicates significant interface shear. Again, the stress distributions in the NDP remain unchanged by the experiment (compare Fig. S13c-f to Fig. S17c-f).

Supplementary Note 5: σ_{zz} vertical virtual sections revealing the stress transfer across the interface

The detailed stress analysis along vertical virtual sections of the SL and ML thin films are presented in Fig. S18. A schematic representing where the data were extracted from the 2D maps is presented in Fig. S18a. As can be seen prior to contact in both SL and ML, there is a slight apparent stress gradient (as discussed already in Suppl. Notes 3 and 4) present in Fig. S18b, which originates in variations of the unstrained lattice parameter (*cf.* Methods).

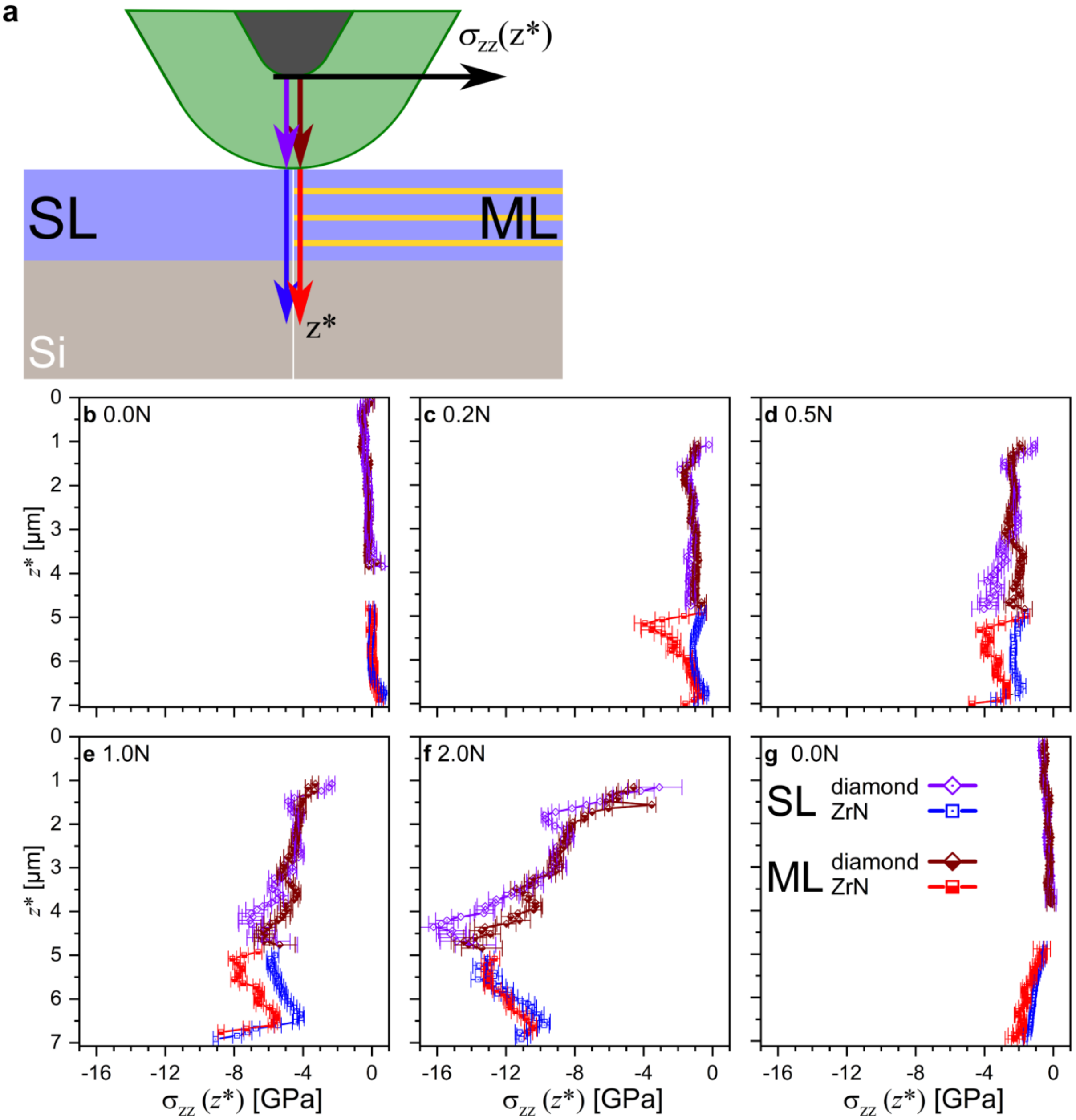

Figure S18. Detailed stress analysis along line intersections. In (a), a schematic is shown, depicting the σ_{zz} and the virtual line intersections as the horizontal and vertical axes, respectively. In (b-g), the σ_{zz} accumulation across the indenter-sample interface is shown for the SL and the ML thin films for the individual load steps.

During the initial loading states (0.2 and 0.5 N) the asperity contact dominates, where individual microstructural features at the surface are highly deformed[30–32], while the rest of the volume remains rather unaffected (Fig. S18c and S18d). At 1.0 and 2.0 N load, the contact is fully established, meaning that the stress values at the contact are similar in the NDP and the SL and ML thin films (Fig. S18e and S18f). Finally, after unloading, the initial state is restored in the NDP for both experiments, while a cross-sectional gradient of increasing compressive σ_{zz} is present in both films (Fig. S18g), which is more prominent in the case of the ML thin film.

Supplementary Note 6: Stress distributions obtained by applying the Hertzian Theory

The elastic stress distributions obtained by using the analytical solutions of the Hertz' contact[21] for two cylinders under load developed by M'Ewen[22] are summarized in Fig. S19. Using the Hertz formulation as described in Eq. S17, contact pressures of 5.4, 8.5, 12.8 and 17.0 GPa are obtained for the cylindrical contact at loads of 0.2, 0.5, 1.0 and 2.0 N, respectively, as presented here. These values are then also the maximum σ_{yy} and σ_{zz} stress values directly at the contact. Additionally, to obtain a better comparison with the experimental data obtained for the thin film, the average residual stress of -2.42 GPa was added to the analytical σ_{yy} stress distribution to better represent the stress state in ZrN (Fig. S19a-d). However, these analytical contact pressures are given for perfect surfaces in perfect contact[21,22,33]. As can be seen from the experimental data presented in Supp. Notes 3-5, the maximum stress values are never reached directly at the contact in the experiment, which is a consequence of the finite X-ray beam size, the surface roughness of the thin films and the NDP in contact (Suppl. Note 10) and possible plastic deformation in the asperities.

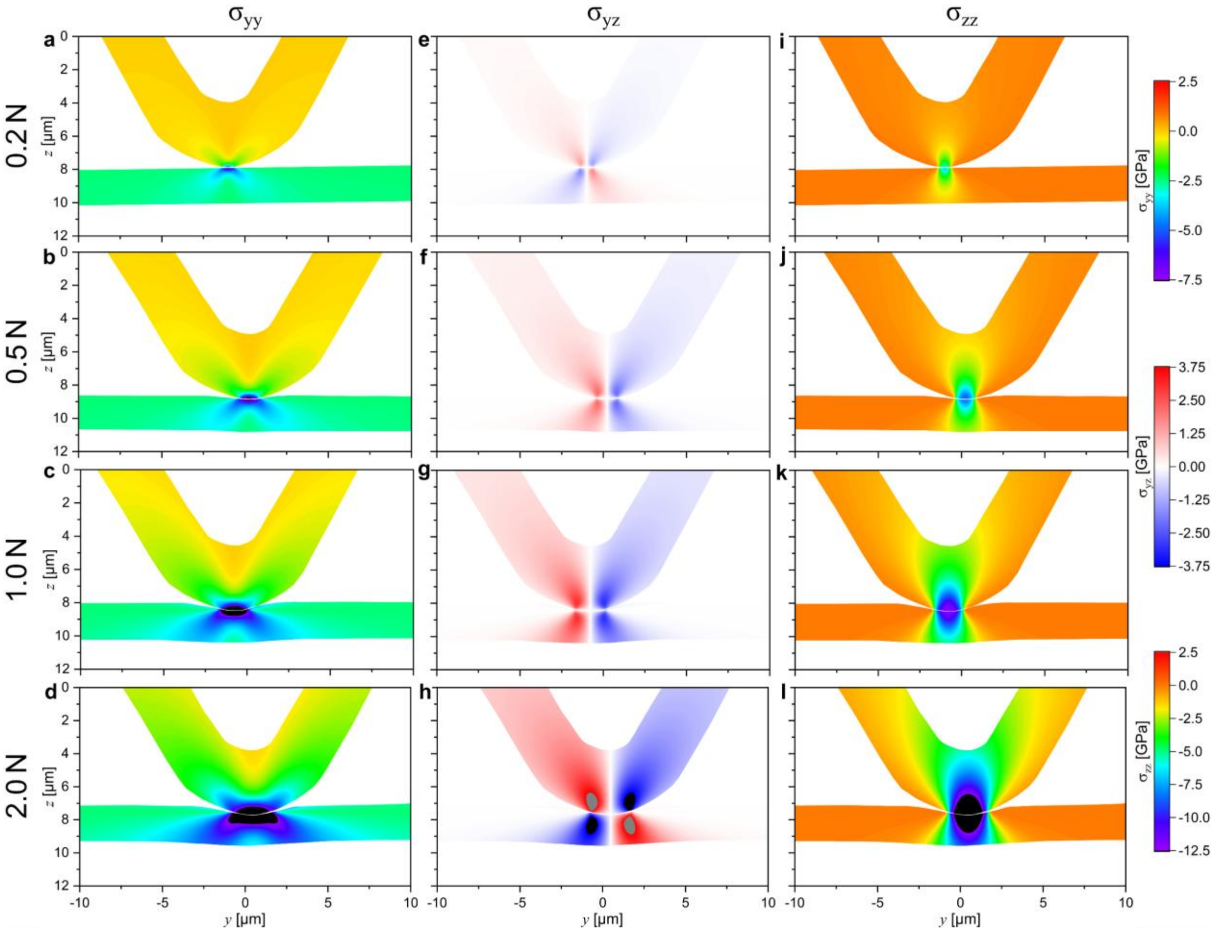

Figure S19. Elastic stress distributions obtained by the analytical solutions of M'Ewen[22] as detailed in the Methods section. In (a-d), the σ_{yy} stress distributions in the system are shown for loads of 0.2, 0.5

and 1.0 N, respectively, while in (e-h) and (i-l) the σ_{zz} and σ_{yz} stress magnitudes are shown at the same loads, respectively.

Nevertheless, outside the immediate contact zone, the analytical stress distributions in Fig. S19 give a surprisingly accurate representation of the experimental data (Suppl. Notes 3 and 4). Especially the shear stress component σ_{yz} presented by the analytical solution (Fig. S19e-h) is in very good agreement with the experimental data of the SL thin film in contact with NDP. Furthermore, from the deviations of the experimental and analytical stress distributions outside the contact zone crucial information about the influence of the various interfaces along the load train (the sc diamond/nc diamond interface and the ZrN/Si interface) and localized plastification or other stress-relief phenomena in the mechanical system can be drawn, which are not accounted for in the analytical considerations[21,22]. For instance, no tensile stresses can be found in the analytical solution of σ_{yy} along the vertical symmetry line (Fig. S19a-c), i.e. the stress only drops to 0 or to -2.42 GPa in case of the diamond and the ZrN, respectively. This contrasts the experimental data presented in Suppl. Note 3 and 4 and Fig. 5, where tensile stresses are found along the vertical intersection in both nc diamond and ZrN. While the outline of the stressed zone found in the experiments is very well represented for the NDP, the enhanced σ_{zz} at the ZrN/Si interface found for both the SL (Suppl. Note 3) and the ML thin films (Suppl. Note 4) is not present in the analytical data. Therefore, these discrepancies between analytical and experimental stress distributions can show the critical influence of interfaces on stress concentrations and failure of materials during the single-asperity contact.

Supplementary Note 7: Detailed Finite Element analysis

The finite element simulation of the indentation process facilitated the investigation of multi-axial stresses in the ML thin film layers subjected to indentation, since the more complex case with elastic-plastic ZrCu interlayers could not be covered by the simplified analytic solution. The contour plots in Fig. S20, S21 and S22 illustrate the 2D distributions of σ_{yy}, σ_{zz} and σ_{yz} components of applied and residual stresses at maximum indentation load (2.0 N) and after unloading (post indentation) for frictionless contact and friction coefficients of $\mu = 0.5$ and 1.0, respectively. They demonstrate good qualitative and quantitative agreement with experimental results given in Figs. 4 and 5. Additionally, simulation results reveal stress hotspots during loading and post-loading conditions, which are not found in the experimental data, possibly because these features could be too small in comparison to the X-ray gauge volume (*cf.* Methods, Suppl. Note 13).

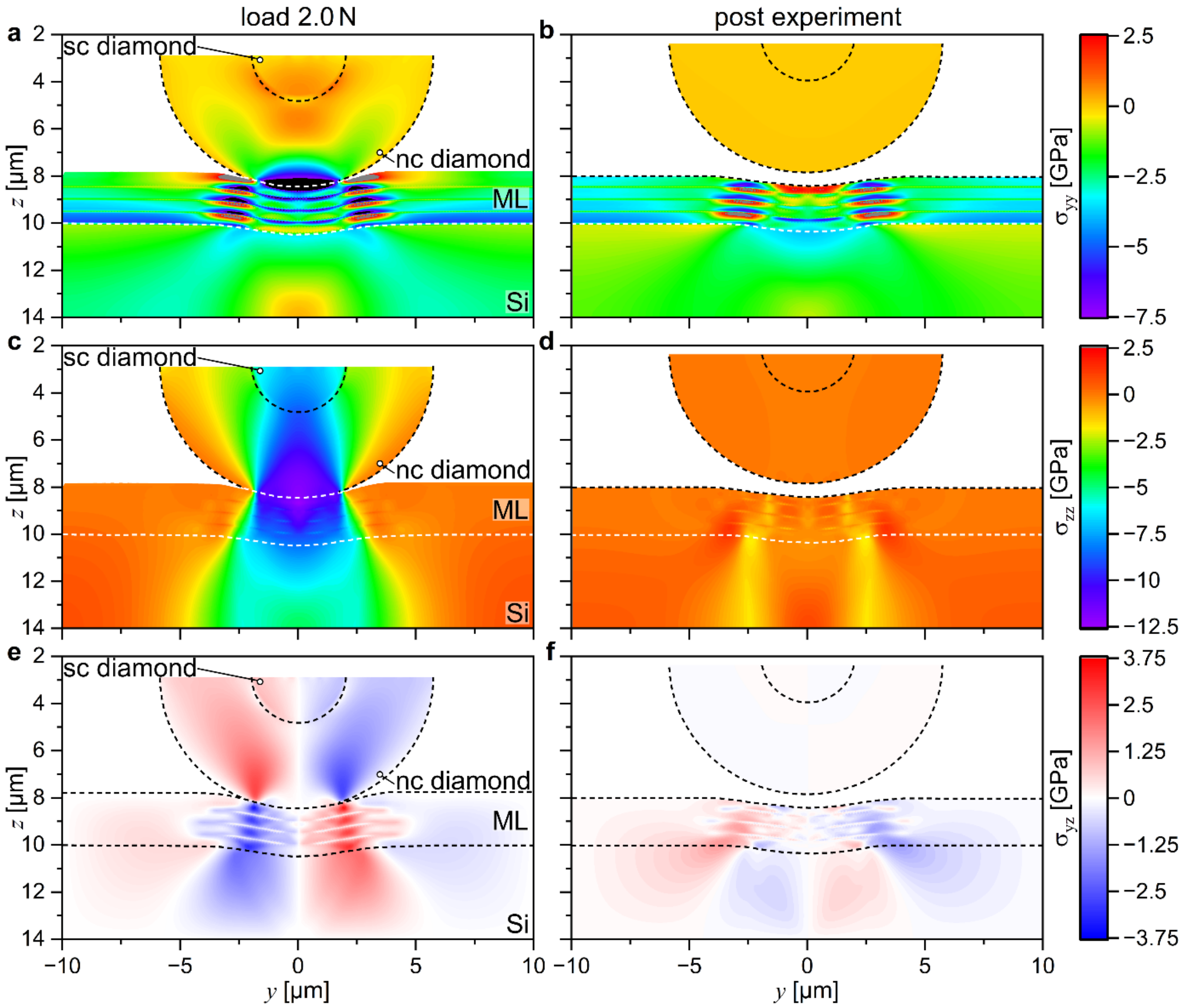

Figure S20. Distribution of σ_{yy}, σ_{zz} and σ_{yz} stress components of applied and residual stresses obtained from frictionless contact at 2.0 N load and after the experiment are presented in (a-c) and (d-f), respectively.

First of all, when comparing the results obtained for the different friction coefficients (Fig. S20-S22), we see that the increasing tangential traction induced by the friction at the interface between nc diamond and ZrN noticeably affects the resulting stress distributions only at the highest friction coefficient of $\mu = 1.0$. But, even at the highest friction coefficients, the stress distributions resulting from the tangential tractions remain much smaller than the normal contact stresses. This is in agreement with existing literature dealing with nanoindentation stress distributions, which found that the influence of the friction force is negligible for indentation considering reasonable friction coefficients below 0.3[34–36]. Additionally, comparable literature data suggests that the friction coefficient between two ceramics is typically in the order of 0.1 or lower[37,38]. Therefore, the following presentation of the results extracted from the FE model will be limited to the numerical data obtained under frictionless contact.

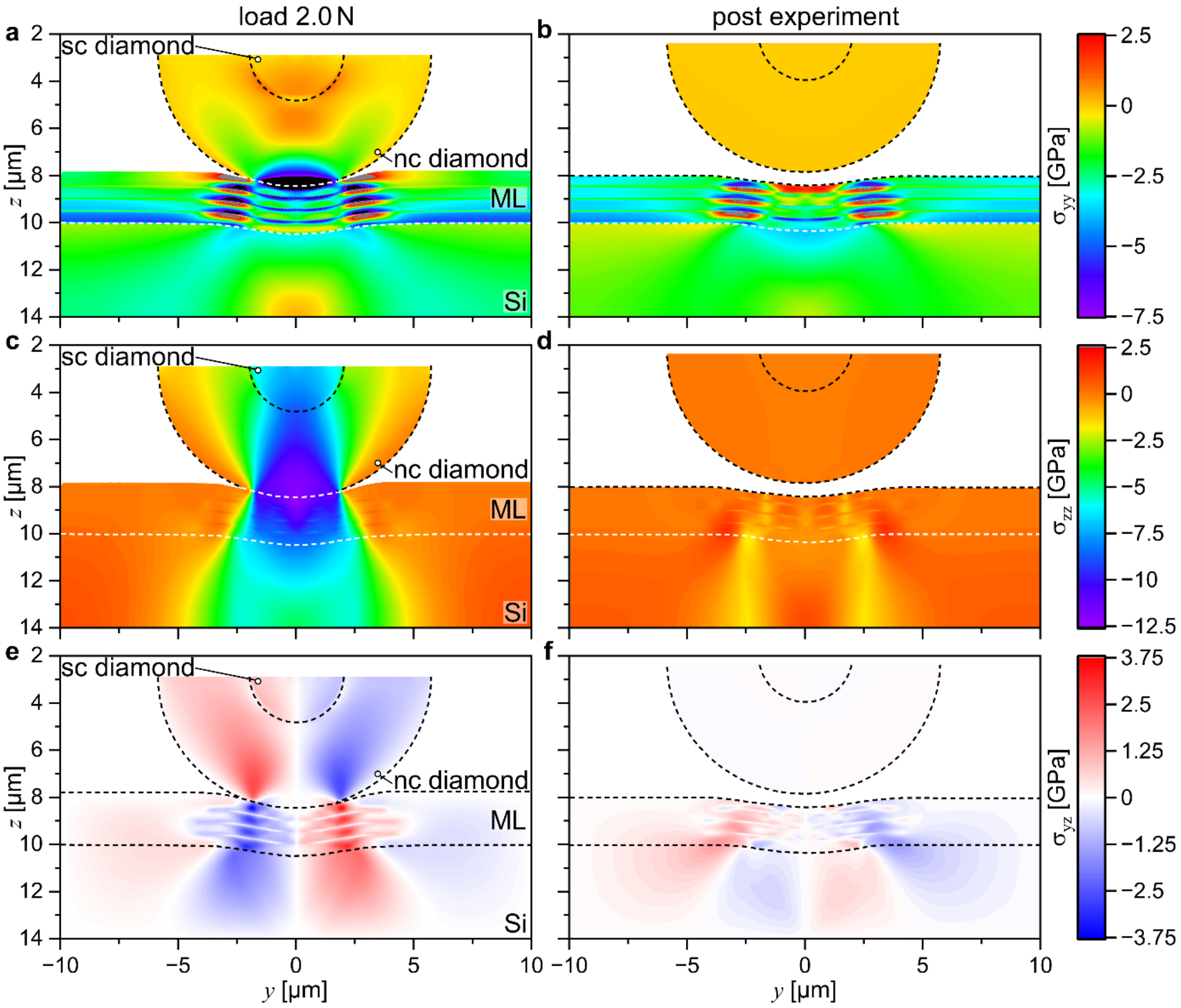

Figure S21. Distribution of σ_{yy}, σ_{zz} and σ_{yz} stress components of applied and residual stresses obtained from contact with a friction coefficient of $\mu = 0.5$ at 2.0 N load and after the experiment are presented in (a-c) and (d-f), respectively.

In case of the ML film, the results presented in Fig. S20 indicate that the distribution of indentation and residual stresses reflect the combined effects of contact compression and the

overall three-point bending of a double-clamped cantilever[20] govern the response of the sample. Of course, the bending-like response is limited by the continuous support given by the Si substrate and can be only used for comparison. Each layer of the ZrN multilayer exhibits bending character, since the presence of ZrCu interlayers represents a weakened transmission of tractions between these layers.

In case of the diamond tip (Fig. S20), an excellent agreement of the numerically obtained stress distributions with the experimental data (Fig. 4) and the analytical model (Fig. S19) is observed. Furthermore, the influence of the sc diamond substrate is highlighted, as the FE model is able to recreate the tensile σ_{yy} magnitudes observed close to the nc/sc diamond interface.

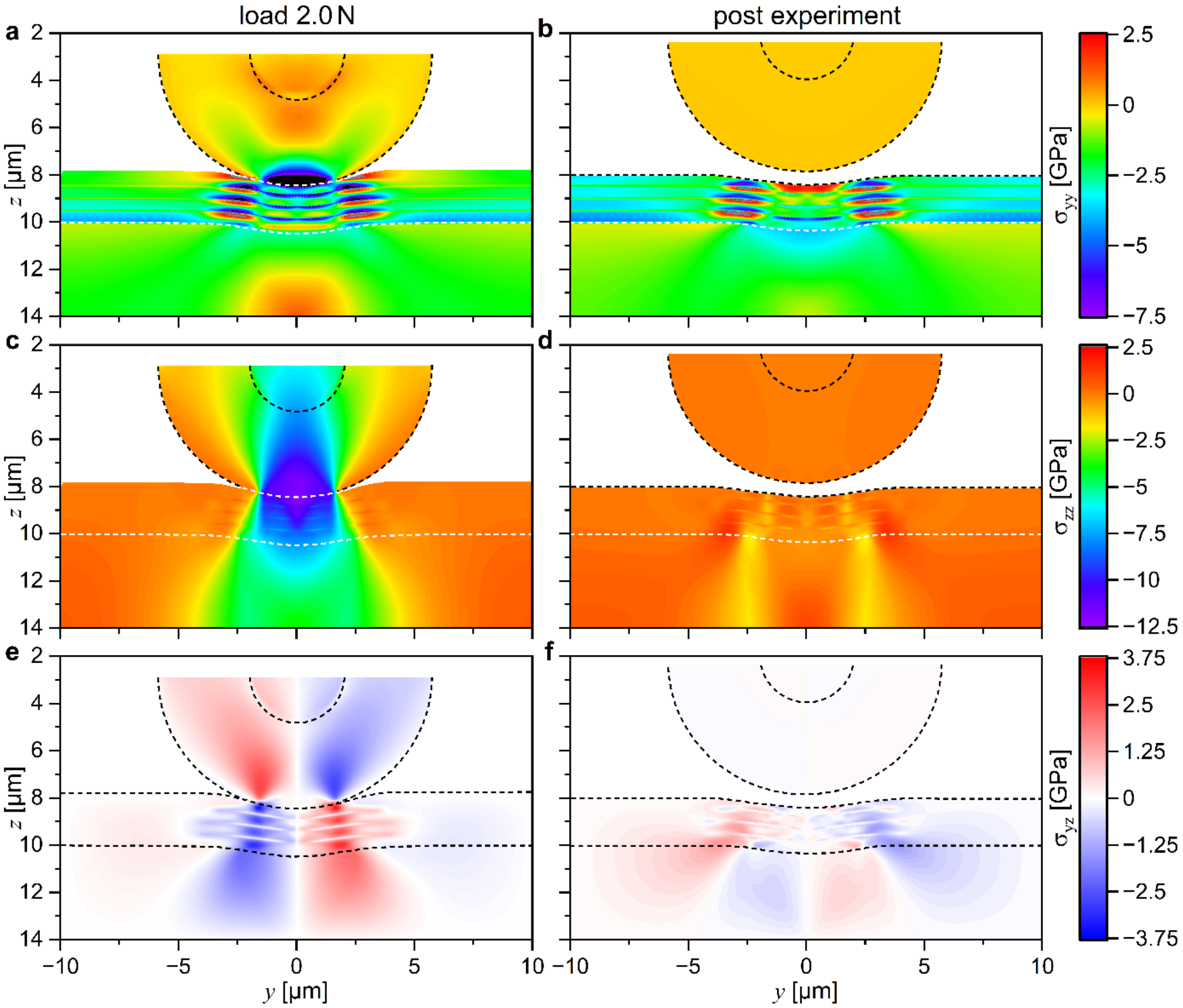

Figure S22. Distribution of σ_{yy}, σ_{zz} and σ_{yz} stress components of applied and residual stresses obtained from contact with a friction coefficient of $\mu = 1.0$ at 2.0 N load and after the experiment are presented in (a-c) and (d-f), respectively.

Furthermore, the experimental study did not allow the determination of stresses in the single crystal Si substrate and the sc diamond. Here, the simulation results reveal the stress distribution in the Si substrate layer and show that the σ_{yy}-component of stress attain high magnitudes in compression during knife edge indentation loading and exhibit a similar, but reduced

distribution in the post-loading state, with an additional stress concentration noted in the sublayer of ZrN lying closest to the Si substrate. Additionally, in the sc diamond minor tensile σ_{yy} stress magnitudes are observed during loading, while σ_{zz} is ~-5 to -7.5 GPa. The high magnitude of σ_{zz}-component compressive stresses during loading relaxes after unloading, but additional eigenstrains formed during indentation cause the formation of tensile residual stresses on both sides of the indentation. Finally, both the nc. and sc diamond appear stress free after unloading, confirming the absence of plasticity in the NDP during indentation.

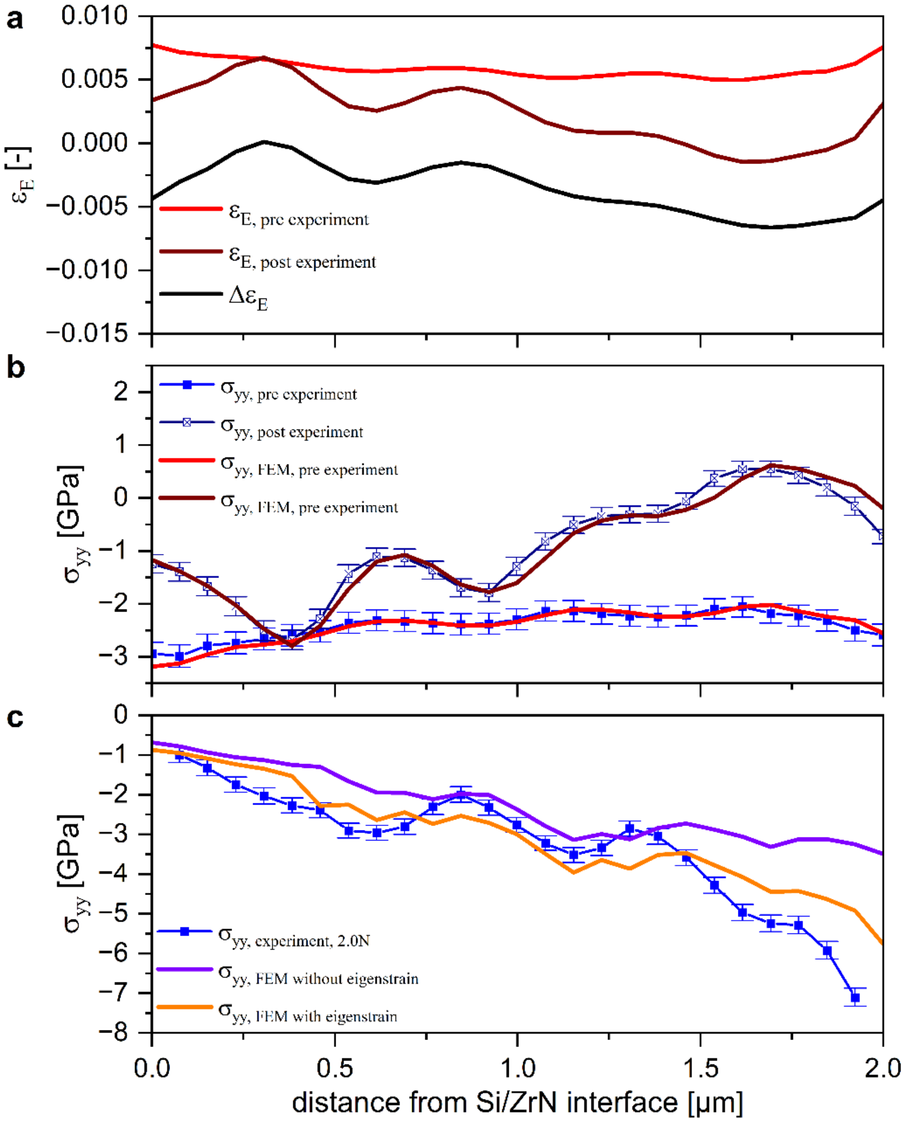

Figure S23. Line plots of eigenstrains before the experiment ($\varepsilon_{E,\text{pre experiment}}$, red), after the experiment ($\varepsilon_{E,\text{post experiment}}$, brown) and the change of the eigenstrain throughout the experiment ($\varepsilon_{E,\text{pre experiment}}$, red) are presented in (a), with respect to the normal distance from the Si/ZrN interface in the center of the contact (y = 0). The modelled and experimental residual stresses before and after the *in situ* CSnanoXRD experiment are shown in (b), while experimental as well as modelled stresses at 2.0 N load are presented in (c) with respect to the normal distance from the Si/ZrN interface in the center of the contact (y = 0).

Shear components of stresses are distributed on both sides of the indentation during both loading and post-loading conditions, with slight deviations in magnitude. These distributions align closely with experimental observations but cover a wider range, including the Si substrate layer. Similar to the hotspots observed for the σ_{yy}-component of stresses, simulation results enable the identification of variations in the shear component at each individual layer.

Eigenstrain reconstruction of residual stresses before and after indentation conditions provided information about the magnitude and distribution of eigenstrains that lead to the formation of residual stresses, as quantified by X-ray diffraction measurements (Fig. S12). It is important to note that these eigenstrains represent the permanent strains responsible for the formation of residual stresses and are illustrated in Fig. S20a. A linear fit of the difference between the two states corresponds to the additional plastic deformation due to indentation, which is similar to that observed in the case of three-point bending[39]. Residual stresses determined by the eigenstrain reconstructions of the two states show excellent agreement with X-ray diffraction residual stress measurements, as illustrated in Fig. S23b.

The influence of eigenstrains formed during the manufacture of biomimetic ML thin film layers on the formation of stresses due to indentation was investigated by performing process simulations with and without initial eigenstrains. The results presented in Fig. S20c demonstrate that initial eigenstrains have a significant impact on the stresses formed during indentation. However, process models in both cases show differences in the ZrN sublayer bound with the Si substrate. This layer deviates into tension, and the differences are attributed to plastic deformation in the Si layer that could not be completely incorporated into the model, as well as the further support located beneath the Si substrate. On the other hand, the process model successfully captured the deviation of the top layer from the overall state into more compression due to contact deformations created by the indenter tip.

Supplementary Note 8: Deposition of the ZrN and ZrCu layers

The ZrCu/ZrN multilayer was deposited by magnetron co-sputtering using an AJA International ATC 2200-V sputter system, which was pumped by a turbomolecular pump (1200 l/s) backed up with a multi-stage roots pump (27 m^3/hr). The base pressure before each deposition was lower than 5 × 10^{-5} Pa. Depositions of the ZrCu sublayers were carried out in argon at a pressure of 0.533 Pa (4 mTorr) using two independent unbalanced magnetrons equipped with circular, indirectly cooled targets (Zr and Cu, 50.8 mm in diameter, 6.35 mm in thickness). On the other hand, the ZrN sublayer was deposited in an argon and nitrogen gas mixture at the same total pressure, utilizing a single Zr target. The magnetron with the Zr target (99.5 % purity, Matsurf Technologies Inc.) was supplied by an Advanced Energy Pinnacle Plus+ 5/5 kW power source working in DC mode for both sublayers, while the magnetron with the Cu target (99.99% purity, Matsurf Technologies Inc.) was supplied by a Hüttinger Elektronik TruPlasma Highpulse 4002 high-power pulsed DC power source working in the high-power impulse regime. The average target power density in a pulse was fixed at 1000 W/cm^2, and the negative voltage pulse length was set to a constant value of 200 ms. The ZrCu/ZrN multilayer was deposited onto polished and ultrasonically pre-cleaned single-crystalline Si(100) substrates held at a floating potential without any external heating. The substrates were rotated above the targets at a speed of 40 rpm and located at a target-to-substrate distance of 150 mm.

Supplementary Note 9: Preparation and testing of the ZrN and ZrN-ZrCu microcantilevers

The cantilevers were processed with by focused ion beam (FIB) in a Zeiss LEO 1540 XB dual beam FIB workstation to final geometries of $L \times B \times t$ ~12×2×3 µm³ operating with milling currents ranging from 10 nA to 500 pA and a polishing current of 100 pA[40,41]. *In situ* bending tests on freestanding microcantilevers were conducted in a Zeiss LEO 1530 scanning electron microscope using a FemtoTools FT-NMT04 indentation system. A wedge indenter tip provided by FemtoTools was used to load the cantilevers until fracture in a displacement-controlled feedback loop with a loading speed of 20 nm/s. The measured load (P) versus deflection (w) data was corrected for the instrument compliance before evaluating the elastic modulus E as follows:

$$E = \frac{4}{B} \times \frac{dP}{dw} \times \left(\frac{L}{t}\right)^3. \tag{S1}$$

Here, $\frac{dP}{dw}$ describes the slope of the linear elastic loading curve, while B and t are the width and thickness of the loaded cantilever, respectively, and L is the distance between the base of the cantilever and the indenter tip. The maximum load P_{max} measured at fracture facilitated the evaluation of the fracture stress σ_{F} as per:

$$\sigma_{\mathrm{F}} = \frac{6P_{\mathrm{max}}L}{Bt^2}. \tag{S2}$$

During the cantilever preparation procedure a bridge notch with a depth of a ~600 nm was added to half of the cantilevers at approximately 2 µm from the base applying an ion current of 10 pA for 180 s[42,43]. In the framework of linear elastic fracture mechanics the fracture toughness K_{IC} was evaluated according to:

$$K_{\mathrm{IC}} = \sigma_{\mathrm{F}}\sqrt{\pi a} \times Y\left(\frac{a}{t}\right). \tag{S3}$$

The dimensionless geometry factor $Y\left(\frac{a}{t}\right)$ as derived in Ref.[44] is given as:

$$Y\left(\frac{a}{t}\right) = \sqrt{\frac{2B}{\pi a}\tan\left(\frac{\pi a}{2B}\right)}\,\frac{0.923+0.199\left(1-\sin\left(\frac{\pi a}{2B}\right)\right)^4}{\cos\left(\frac{\pi a}{2B}\right)}. \tag{S4}$$

Supplementary Note 10: Preparation of the NDP

A top view SEM image of the indenter tip is presented in Fig. S24a, showing the 60 and 90 deg opening angles perpendicular and parallel to the wedge, respectively. The total length of the wedge after deposition was around ~86 µm. A more detailed top view of the nanocrystalline diamond thin film is given in Fig. S24b, where the cube structures on the top surface can be interpreted as (100) facets of growing crystallites. Typically, {100} oriented diamond grows during the CVD process at elevated temperatures (compared to the growth of {111} oriented facets at lower temperatures[45,46]). Since the indenter tip was oriented towards the gas precursor flow and the heater filaments, it may have been exposed to a higher gas flow and higher temperature compared to the side faces. This effect is further confirmed by the averaged FWHM analysis of the *in situ* experiment (Suppl. Notes 3 and 4), where comparably lower FWHM was evaluated at the tip of the indenter.

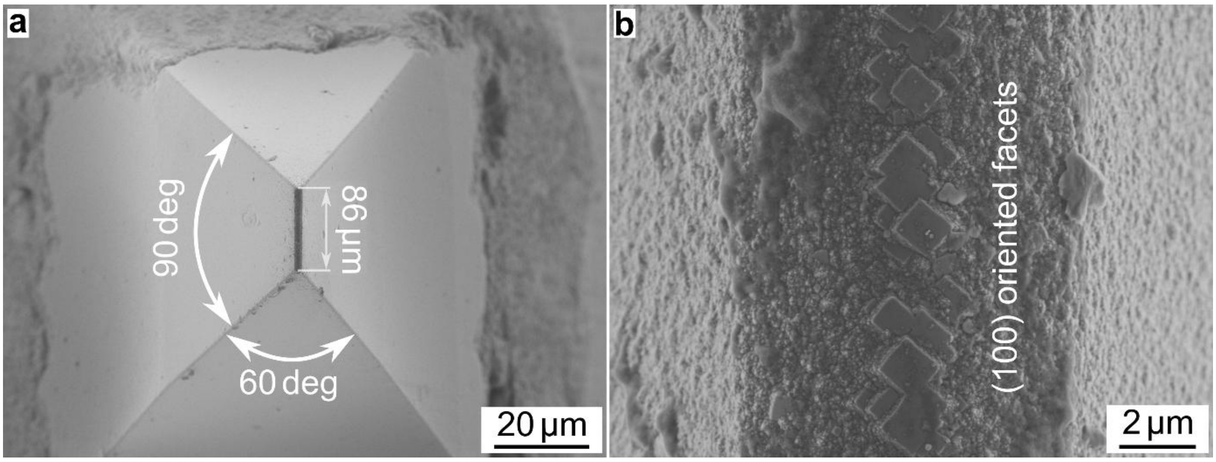

Figure S24. The indenter tip after deposition of NCD. In (a) the top view of the tip is presented, while in (b) the high resolution image shows (100) oriented cubes directly at the tip. The scale bars in (a) and (b) are equal to 100 µm and 2 µm, respectively.

A top view SEM image of the indenter tip after FIB processing is presented in Fig. S25a, the straight edges perpendicular to the wedge, respectively. The total length of the wedge after deposition was measured as 82.3 µm. A more detailed side view of the nanocrystalline diamond thin film is given in Fig. S25b, where it can be seen that the total film thickness was 3.8 µm and the radius of the tip was evaluated as 5.8 µm.

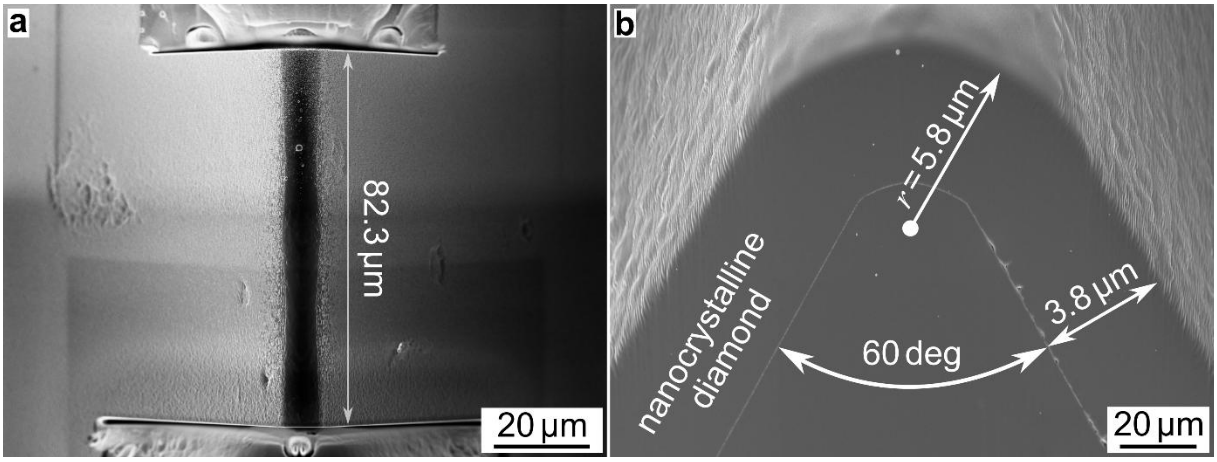

Figure S25. The indenter tip after final preparation. In (a) the top surface of the tip is shown, while in (b) the side view is presented.

Supplementary Note 11: *In situ* CSnanoXRD setup, NDP and sample alignment and stress analysis method

The custom indentation setup positioned at the nanofocus extension of the ID13 beamline at the ESRF is presented in Fig. S26. The indenter is equipped with two SLC1720 linear positioning axis (Smaract GmbH, Oldenburg Germany) as well as a CGO-60.5 goniometer stage (Smaract GmbH, Oldenburg Germany). This combination of positioners allows an independent sample alignment and positioning along the x, y, and φ axes. N-310 NEXACT OEM Miniature Linear Motor (PI (Physik Instrumente) L.P., Shrewsbury, Massachusetts, USA) was used for the vertical (z) movement of the indenter tip, which was monitored by a RLB Miniature Incremental Magnetic Encoder Module (RLS d.o.o., Ljubljana, Slovenia) with a displacement resolution of 250 nm. The force was monitored using a KD24 load cell (ME-Systeme GmbH, Germany with a nominal range of 2 N and an accuracy class of 0.1%).

The indenter tip was aligned parallel to the incident X-ray beam performing series of absorption scans along the y and z axes at various positions of the φ and ξ rotational axes of the hexapod positioner, respectively (Fig. S26). The optimal orientation of the tip was identified by maximizing the absorption contrast between the nc diamond and air as well as between the nc and sc diamond. The sample was oriented by additional absorption scan along the z-axis at various tilts along the inner ξ rotational axis of the indenter setup (Fig. S26). Again, the optimal orientation was determined by maximizing the absorption contrast between the thin film and the air.

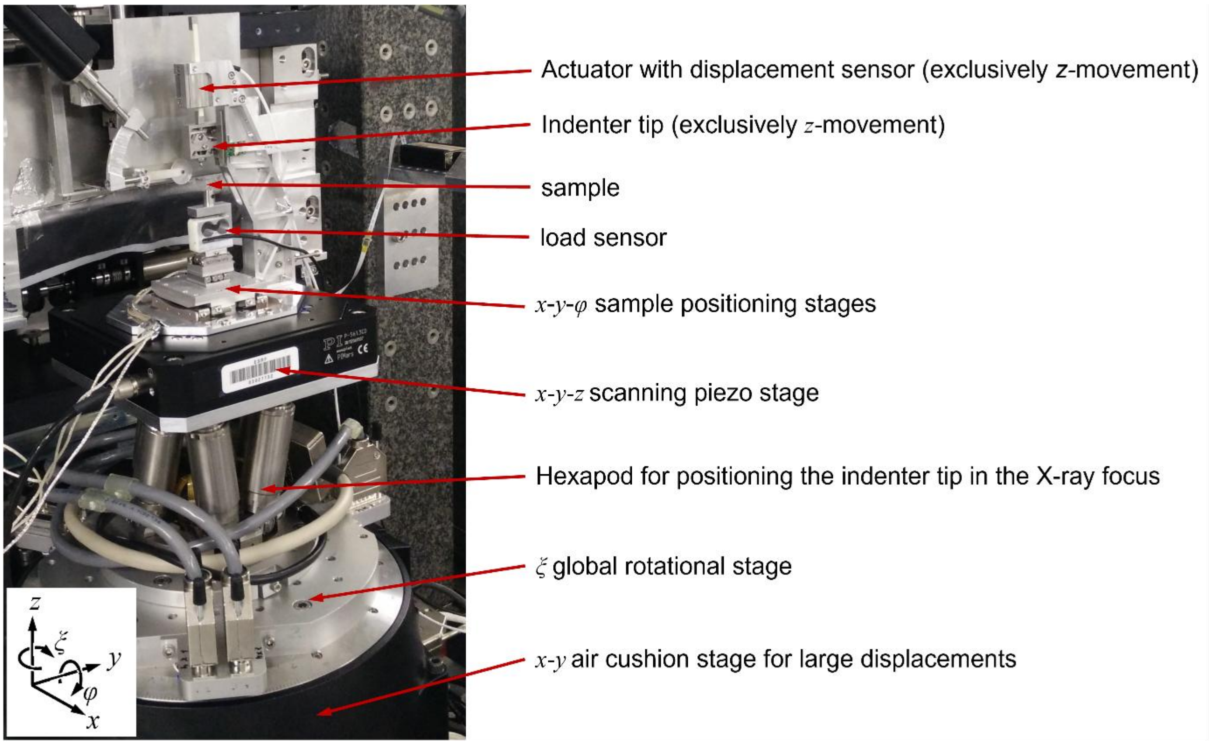

Figure S26. The indenter setup positioned at the nanofocus extension of the ID13 beamline of the ESRF.

For quantification of stress distributions from CSnanoXRD data, the measured orientation-dependent lattice strain $\varepsilon_{\delta\theta}^{m,hkl}(y,z)$ can be expressed for each thin film material *m* (ZrN, diamond) and each DS ring *hkl* as follows:

$$\varepsilon_{\delta\theta}^{m,hkl}(y,z) = \frac{d_{\delta\theta}^{m,hkl}(y,z) - d_0^{m,hkl}}{d_0^{m,hkl}} \tag{S5}$$

where θ is the diffraction angle, δ the azimuthal angle on the detector (see. Fig. 1), while $d_{\delta\theta}^{m,hkl}(y,z)$ is the measured lattice plane spacing and $d_0^{m,hkl} = a_{0,f}^m/\sqrt{(h^2+k^2+l^2)}$ is the strain-free lattice plane spacing for the two cubic materials (ZrN, diamond). By following the assumption that the out-of-plane stress component σ_{zz}^m is negligible before loading, the strain-free lattice parameters were determined as $a_{0,}^{\mathrm{diamond}}$ = 3.56976 nm and $a_{0,}^{\mathrm{ZrN}}$ = 4.60632 nm. These precise values generally encompass slight differences between bulk materials and thin films, as well as miniscule residual calibration errors. The orientation-dependent lattice strain $\varepsilon_{\delta\theta}^{m,hkl}(y,z)$ of the measured material is a function of unknown triaxial strain components $\varepsilon_{ij}^{m,hkl}(y,z)$, defined in the sample coordinate system (*i,j* ϵ *x,y,z*) as follows:

$$\varepsilon_{\delta\theta}^{m,hkl}(y,z) = \sin^2\theta\, \varepsilon_{xx}^{m,hkl}(y,z) + \cos^2\theta \sin^2\delta\, \varepsilon_{yy}^{m,hkl}(y,z) + \cos^2\theta\cos^2\delta\, \varepsilon_{zz}^{m,hkl}(y,z) - \sin 2\theta \cos\delta\, \varepsilon_{xz}^{m,hkl}(y,z) + \cos^2\theta \sin 2\delta\, \varepsilon_{yz}^{m,hkl}(y,z) - \sin 2\theta \sin\delta\, \varepsilon_{xy}^{m,hkl}(y,z) \tag{S6}$$

Applying the X-ray elastic constants $S_1^{m,hkl}$ and $\frac{1}{2}S_2^{m,hkl}$ allows to transform the strain components $\varepsilon_{ij}^{m,hkl}(y,z)$ to the components of the stress tensor $\sigma_{ij}^m(y,z)$ defined in the sample coordinate system (*i,j* ϵ *x,y,z*) as follows:

$$\varepsilon_{\delta\theta}^{m,hkl}(y,z) = S_1^{m,hkl}\left[\sigma_{xx}^m(y,z) + \sigma_{yy}^m(y,z) + \sigma_{zz}^m(y,z)\right] + \frac{1}{2}S_2^{m,hkl}\left[\sin^2\theta\, \sigma_{xx}^m(y,z) + \cos^2\theta\sin^2\delta\, \sigma_{yy}^m(y,z) + \cos^2\theta\cos^2\delta\, \sigma_{zz}^m(y,z) - \sin 2\theta\cos\delta\, \sigma_{xz}^m(y,z) + \cos^2\theta \sin 2\delta\, \sigma_{yz}^m(y,z) - \sin 2\theta \sin\delta\, \sigma_{xy}^m(y,z)\right] \tag{S7}$$

X-ray elastic constants were determined for the ZrN 111, 200, 220 and 311 DS rings as well as the diamond 111 DS ring using the Hill-type grain interaction model[47] and single-crystal elastic constants of C_{11}^{ZrN}=454 GPa, C_{12}^{ZrN}=121 GPa and C_{44}^{ZrN}=124 GPa as well as $C_{11}^{\mathrm{diamond}}$=1079 GPa, $C_{12}^{\mathrm{diamond}}$=124 GPa and $C_{44}^{\mathrm{diamond}}$=578 GPa obtained from literature for ZrN[23] and diamond[48], respectively.

Furthermore, it was supposed that the in-plane stress component $\sigma_{xx}^m(y,z)$ along the *x*-axis did not change significantly during the *in situ* experiment and therefore can be approximated as constant with values equal to the residual stress evaluated prior to loading. Moreover, it was supposed that $\sigma_{xz}^{m,}(y,z)$ and $\sigma_{xy}^m(y,z)$ can be neglected. This should be a valid simplification and follows from the reduced dimensionality of the sample and indenter probe geometries. The

unknown stress components ($\sigma_{yy}^m(y,z)$, $\sigma_{yz}^m(y,z)$, $\sigma_{zz}^m(y,z)$) in the nc diamond and ZrN were thus evaluated as follows:

$$\varepsilon_{\delta\theta}^{m,hkl}(y,z) = S_1^{m,hkl}\left[\sigma_{xx}^m + \sigma_{yy}^m(y,z) + \sigma_{zz}^m(y,z)\right] + \frac{1}{2}S_2^{m,hkl}\left[\sin^2\theta\,\sigma_{xx}^m + \cos^2\theta\sin^2\delta\,\sigma_{yy}^m(y,z) + \cos^2\theta\cos^2\delta\,\sigma_{zz}^m(y,z) + \cos^2\theta\sin 2\delta\,\sigma_{yz}^m(y,z)\right] \qquad \text{(S8)}$$

Subsequently, for every measured point, a system of up to 144 linear equations (corresponding to 36 δ orientations on the detector and 4 reflections in case of ZrN) based on Eq. 4 was established[49] and the three unknown stress components ($\sigma_{yy}^m(y,z)$, $\sigma_{yz}^m(y,z)$, $\sigma_{zz}^m(y,z)$) were refined for ZrN and diamond, respectively.

Finally, in order to get further information about the deformation processes, an equivalent stress according to the von Mises yield criterion, $\sigma_{\text{von Mises}}^m(y,z)$, was calculated from ($\sigma_{yy}^m(y,z)$, $\sigma_{yz}^m(y,z)$, $\sigma_{zz}^m(y,z)$) as follows:

$$\sigma_{\text{von Mises}}^m(y,z) = \sqrt{\left(\sigma_{yy}^m(y,z)\right)^2 - \sigma_{yy}^m(y,z)\cdot\sigma_{zz}^m(y,z) + \left(\sigma_{zz}^m(y,z)\right)^2 + 3\cdot\left(\sigma_{yz}^m(y,z)\right)^2} \qquad \text{(S9)}$$

Finally, in case of diamond, the stress magnitudes have to be scaled up due to the larger thickness of the indenter tip in comparison with the indented thin films (Fig. 1), which is done by the following equation: $\sigma_{ij}^{\text{diamond,true}}(y,z) = \frac{82.3\ \mu\text{m}}{56\ \mu\text{m}}\sigma_{ij}^{\text{diamond}}(y,z)$. This simple relation assumes that the parts of the wedged indenter tip extending past the thin film lamella thickness do not carry a load and therefore lower the measured stress values since they are volume-averaged within the X-ray gauge volume.

The stored elastic energy per line element (in beam direction) E_{el} stored during deformation in the SL and ML samples was calculated from the $\sigma_{\text{von Mises}}^{\text{ZrN}}(y,z)$ at each load P stress distributions in analogy to Lassnig *et al.*[50] as follows

$$E_{el}^P = \sum_i \frac{1-\nu_\text{i}{}^2}{2}\frac{\sigma_{\text{von Mises}}^\text{i}(y,z)^2\Delta z\cdot\Delta y}{E_\text{i}}, \qquad \text{(S10)}$$

where ν_i and E_i are the Young's modulus and Poisson's ratio of the investigated material, respectively, while Δy and Δz equal the step size of 80 nm used for the CSnanoXRD experiments. The elastic constants used in this study are summarized in Table S1, in Suppl. Note 13. In order to exclude the influence of residual stress present in ZrN before loading, the change of elastic energy is calculated by $\Delta E_{el}^P = E_{el}^P - E_{el}^{0\text{N}}$ for each load step. In case of the NDP, only the data up to a distance of 5 µm from the tip was included in the calculation to ensure a constant volume for the calculation of the strain energy.

Supplementary Note 12: Analytical elastic and hardness considerations of the cylinder contact

The contact problem given by the nc diamond coated indenter tip and the SL thin film can be interpreted by the elastic solution of the cylindrical contact given by Hertz[21,33]. For this solution, we have to consider the reduced modulus E^*, which is defined as follows

$$\frac{1}{E^*} = \frac{1-\nu_{\mathrm{ZrN}}^2}{E_{\mathrm{ZrN}}} + \frac{1-\nu_{\mathrm{nc\ diamond}}^2}{E_{\mathrm{nc\ diamond}}}, \tag{S11}$$

where $E_{\mathrm{ZrN}} = 200$ GPa and $E_{\mathrm{nc\ diamond}} = 460$ GPa are the Young's moduli of ZrN and diamond obtained from the microcantilever bending experiments[9], while $\nu_{\mathrm{ZrN}} = 0.25$ and $\nu_{\mathrm{nc\ diamond}} = 0.15$ were estimated from literature[51]. Additionally, the average radius of contact R has to be considered, which is defined as follows

$$\frac{1}{R} = \frac{1}{R_{\mathrm{ZrN}}} + \frac{1}{R_{\mathrm{nc\ diamond}}}, \tag{S12}$$

where R_{ZrN} is considered to be infinite and thus $R = R_{\mathrm{nc\ diamond}} = 5.8$ µm, as determined by the SEM analysis of the indenter tip. These two considerations allow to evaluate the elastic contact pressure p_0, which is defined as

$$p_0 = \sqrt{\frac{P \cdot E^*}{\pi \cdot l \cdot R}}, \tag{S13}$$

where P and l are the applied load and the length of contact, respectively. Finally, the half-length of the contact is defined as follows

$$a^2 = 4\,\frac{P \cdot R}{\pi \cdot E^* \cdot l}, \tag{S14}$$

Using this considerations by Hertz, Ewen M'Ewen[22] solved the 2D stress distributions of the line contact of two cylinders by introducing two generalized coordinates m and n, which are defined as

$$m^2 = \frac{1}{2}\left[\left(\sqrt{(a^2 - y^2 + z^2)^2 + 4y^2z^2}\right) + (a^2 - y^2 + z^2)\right] \tag{S15}$$

and

$$n^2 = \frac{1}{2}\left[\left(\sqrt{(a^2 - y^2 + z^2)^2 + 4y^2z^2}\right) - (a^2 - y^2 + z^2)\right], \tag{S16}$$

where $y = 0$ and $z = 0$ are defined as the point of contact, while m and n have the same signs as z and y, respectively. The 2D stress tensor can then be described as

$$\begin{pmatrix} \sigma_{\mathrm{yy}} \\ \sigma_{\mathrm{zz}} \\ \sigma_{\mathrm{yz}} \end{pmatrix} = -\frac{p_0}{a} \cdot \begin{Bmatrix} m\left(1 + \frac{z^2+n^2}{m^2+n^2}\right) - 2z \\ m\left(1 - \frac{z^2+n^2}{m^2+n^2}\right) \\ n\left(\frac{m^2-z^2}{m^2+n^2}\right) \end{Bmatrix}. \tag{S17}$$

The hardness of the cylindrical contact[52] is defined in analogy to the Meyer hardness[53] as follows:

$$H_c \approx 2.24 \cdot \sigma_y, \quad \text{(S18)}$$

where σ_y is the yield stress of the indented material. Reversing the argument of Tabor and later works[16,17], where the indentation hardness H_i of a material is related to the yield strength by a factor of 2.8-3, the hardness of the cylindrical contact can be defined as follows:

$$H_c \approx 0.75 \cdot H_i - 0.8 \cdot H_i. \quad \text{(S19)}$$

Furthermore, the analogy to the Meyer hardness[53] can be driven further, using the sample thickness (l = 56 µm) in beam direction, the applied load (P = 2.0 N) and the Hertz' contact width (2a = 2.66 µm) to derive the contact hardness as follows:

$$H_c = \frac{P}{l \cdot 2a}. \quad \text{(S20)}$$

Inserting the individual parameters, a contact hardness H_c of ~13.4 GPa is obtained from the experimental data.

Supplementary Note 13: Eigenstrain-based FE analysis to recreate the stress distributions in the ML thin film

The contact problem was simulated using the finite element (FE) method for the ML film. Unlike conventional process models, the simulation was designed to accommodate the eigenstrains[39,54,55] inherited from the deposition of thin film layers. This involved defining the eigenstrain field assumed to vary only along the surface normal (z-axis) expressed as a high-order polynomial function detailed in Eq. S18. The implementation uses the formulation derived from a previous study that introduced the voxel-based eigenstrain reconstruction method[55]. Parameters for the 14th-order polynomial function were determined through least squares fitting by matching the reconstructed residual stresses to the profile of the σ_{yy} stress component obtained from synchrotron X-ray diffraction analysis:

$$e^*(z) = p_0 + p_1 z^n + p_2 z^{n-1} + \ldots + p_n z^1 + p_{n+1} \quad \text{(S21)}$$

Only the σ_{yy} stress component of eigenstrain was imposed based on the following assumptions that equibiaxial eigenstrains form along the y- and z-axes during layer deposition, eigenstrains along the x-axis (beam direction) do not affect the residual stress and deformation behavior due to lack of constraint in that direction in the thin coupon studied and eigenstrains along the z-axis do not affect the residual stress state due to the proximity of the surface that causes relief.

The indentation process was simulated by FE modelling of the contact between the indenter and the multilayer. The coated layers (ZrN, CuZr, and the Si substrate) and the indenter (sc and nc diamond) were meshed using 3D, 8-node linear solid continuum elements (C3D8 and C3D8R) to accurately capture the stress tensor during deformation while satisfying computational efficiency. To achieve this, the indenter was represented as a cylindrical body with a radius of 5.8 µm, consisting of an inner layer of sc diamond with a radius of 2 µm and an outer layer formed from nc diamond with a thickness 3.8 µm corresponding to the physical dimensions of the knife edge. The domain encompassing the ZrCu-ZrN thin film layers and the Si substrate was modelled as a single part with distinct material properties. The dimensions of the ML film in the yz-plane were set at 20 by 6.06 µm. A fine mesh density with an element size of 125 nm was employed throughout the contact zone and critical deformation regions to ensure numerical accuracy and proper resolution of the stress gradients during indentation. Additionally, the cylindrical indenter tip and orthogonal body of the ML film were created with an thickness of 125 nm equal to the size of two cubic hexahedral elements. A symmetry condition defined at all surfaces of the hexagonal body except the indentation surface satisfied modelling the continuity of the model domain. The bottom face of the Si substrate was fully constrained in the vertical displacement direction ($u_2 = 0$). The outer faces along the y-axis were

constrained from moving in the *y*-direction ($u_1 = 0$), and the front and back faces along the *x*-axis were constrained in the *x*-direction ($u_3 = 0$) to simulate a continuous coating. A linear elastic-ideal plastic constitutive model (zero strain hardening) was utilized for the all materials, while material properties of this finite element model are given in Table S1.

Table S1. Material properties of the multilayer thin film layers

	Young's Modulus [GPa]	Poisson's Ratio [-]	Yield Strength [GPa]
ZrN[23]	380	0.26	7.0
ZrCu[27]	55	0.35	1.8
Si[28]	155.0	0.20	4.3
nc diamond[9,56]	460	0.15	22
sc diamond[57]	1120	0.07	-

Furthermore, the interaction between the indenter and the top ZrN coating surface was defined using a surface-to-surface contact formulation with finite sliding kinematics. To systematically investigate the effect of energy dissipation and shear stress transfer at the interface, the coefficient of friction (μ) was parametrically analyzed at values of 0.0 (frictionless), 0.5, and 1.0 utilizing an isotropic penalty friction formulation. The analysis was solved implicitly using the standard within the commercial Abaqus software. Nonlinear geometric effects were enabled to account for large deformations and contact updates during the deep indentation process. Standardized convergence criteria within the commercial Abaqus software were utilized for force and displacement residuals, with an initial increment size of 0.01 and a minimum allowable time increment of 10^{-8} to ensure strict numerical stability during the highly nonlinear contact phase.

References

1. John. A. Thornton. Structure-Zone Models of Thin Films. *Model. Opt. Thin Film.* **821**, 95–103 (1987).

2. Anders, A. A structure zone diagram including plasma-based deposition and ion etching. *Thin Solid Films* **518**, 4087–4090 (2010).

3. Pogačnik, A. & Kalin, M. How to determine the number of asperity peaks, their radii and their heights for engineering surfaces: A critical appraisal. *Wear* **300**, 143–154 (2013).

4. Daniel, R. *et al.* Multilayer design of sustainable multifunctional Zr – Cu – N coatings : A route for enhanced mechanical and antibacterial performance. *Mater. Des.* **254**, (2025).

5. Daniel, R. *et al.* Mono-textured nanocrystalline thin films with pronounced stress-gradients: On the role of grain boundaries in the stress evolution. *J. Appl. Phys.* **115**, (2014).

6. Daniel, R., Martinschitz, K. J., Keckes, J. & Mitterer, C. The origin of stresses in magnetron-sputtered thin films with zone T structures. *Acta Mater.* **58**, 2621–2633 (2010).

7. Keckes, J. *et al.* X-ray nanodiffraction reveals strain and microstructure evolution in nanocrystalline thin films. *Scr. Mater.* **67**, 748–751 (2012).

8. Keckes, J. *et al.* 30 nm X-ray focusing correlates oscillatory stress, texture and structural defect gradients across multilayered TiN-SiOx thin film. *Acta Mater.* **144**, 862–873 (2018).

9. Meindlhumer, M. *et al.* Micromechanical Properties of Micro- and Nanocrystalline CVD Diamond Thin Films with Gradient Microstructures and Stresses. *J. Vac. Sci. Technol. A* **42**, 023401 (2024).

10. Watanabe, H., Yamada, N. & Okaji, M. Linear thermal expansion coefficient of silicon from 293 to 1000 K. *Int. J. Thermophys.* **25**, 221–236 (2004).

11. Woehrl, N., Hirte, T., Posth, O. & Buck, V. Investigation of the coefficient of thermal expansion in nanocrystalline diamond films. *Diam. Relat. Mater.* **18**, 224–228 (2009).

12. Jacobson, P. & Stoupin, S. Thermal expansion coefficient of diamond in a wide temperature range. *Diam. Relat. Mater.* **97**, 107469 (2019).

13. Krishan, R. S. Thermal Expansion of Diamond. *Nature* **154**, 486–487 (1944).

14. Moelle, C. *et al.* Measurement and calculation of the thermal expansion coefficient of diamond. *Diam. Relat. Mater.* **6**, 839–842 (1997).

15. Thewlis, J. & Davey, A. R. XL. Thermal expansion of diamond. *Philos. Mag.* **1**, 409–414 (1956).

16. Tirupataiah, Y. & Sundararajan, G. On the constraint factor associated with the indentation of work-hardening materials with a spherical ball. *Metall. Trans. A* **22**, 2375–2384 (1991).

17. Tabor, D. *The hardness of metals*. (Oxford University Press, 1951).

18. Meindlhumer, M. *et al.* Nanoscale stress distributions and microstructural changes at scratch track cross-sections of a deformed brittle-ductile CrN-Cr bilayer. *Mater. Des.* **195**, 109023 (2020).

19. Meindlhumer, M. *et al.* Resolving the Fundamentals of the J-integral Concept by Multi-Method in situ Nanoscale Stress-Strain Mapping. *Commun. Mater.* **6**, 15 (2025).

20. Meindlhumer, M. *et al.* Evolution of stress fields during crack growth and arrest in a brittle-ductile CrN-Cr clamped-cantilever analysed by X-ray nanodiffraction and modelling. *Mater. Des.* **198**, 109365 (2021).

21. Hertz, H. Über die Berührung fester elastischer Körper. *J. für die reine und Angew. Math.* **171**, 156–171 (1881).

22. M'Ewen, E. XLI. Stresses in elastic cylinders in contact along a generatrix (including the effect of tangential friction). *London, Edinburgh, Dublin Philos. Mag. J. Sci.* **40**, 454–459 (1949).

23. Christensen, A. N., Dietrich, O. W., Kress, W. & Teuchert, W. D. Phonon anomalies in transition-metal nitrides: ZrN. *Phys. Rev. B* **19**, 5699–5703 (1979).

24. Feigin, L. A. & Svergun, D. I. *Structure Analysis by Small-Angle X-Ray and Neutron Scattering*. (Springer New York, N, 1987). doi:https://doi.org/10.1007/978-1-4757-6624-0.

25. Kiessig, H. Untersuchungen zur Totalreflexion von Röntgenstrahlen. *Ann. Phys.* **402**, 715–768 (1931).

26. Holy, V., Kubena, J., Ohlidal, I., Lischka, K. & Plotz, W. X-ray reflection from rough layered systems. *Phys. Rev. B* **47**, 15896–15903 (1993).

27. Haviar, S. *et al.* Nanoindentation and microbending analyses of glassy and crystalline Zr(-Hf)-Cu thin-film alloys. *Surf. Coatings Technol.* **399**, 126139 (2020).

28. Hopcroft, M. a, Nix, W. D. & Kenny, T. W. What is the Young's Modulus of Silicon? *J. Microelectromechanical Syst.* **19**, 229–238 (2010).

29. S.Timoshenko. *Strength Of Materials part I and II*. (D. Van Nostrand Company, Inc., 1940).

30. Sharma, A. & Jackson, R. L. A Finite Element Study of an Elasto-Plastic Disk or Cylindrical Contact Against a Rigid Flat in Plane Stress with Bilinear Hardening. *Tribol. Lett.* **65**, 1–12 (2017).

31. Chang, W. R. An elastic-plastic contact model for a rough surface with an ion-plated soft metallic coating. *Wear* **212**, 229–237 (1997).

32. Demirkurt, B. *et al.* Resolving Multi-Asperity Contacts at the Nanoscale through Super-Resolution Fluorescence Imaging. *J. Phys. Chem. Lett.* **15**, 1936–1942 (2024).

33. Johnson, K. L. *Contact Mechanics*. (1985). doi:10.1017/CBO9781139171731.

34. Liu, Y. *et al.* Combined numerical simulation and nanoindentation for determining mechanical properties of single crystal copper at mesoscale. *J. Mech. Phys. Solids* **53**, 2718–2741 (2005).

35. Wang, Y., Raabe, D., Klüber, C. & Roters, F. Orientation dependence of nanoindentation pile-up patterns and of nanoindentation microtextures in copper single crystals. *Acta Mater.* **52**, 2229–2238 (2004).

36. Huang, X. & Pelegri, A. A. Finite element analysis on nanoindentation with friction contact at the film/substrate interface. *Compos. Sci. Technol.* **67**, 1311–1319 (2007).

37. Kılınç, B., Kocaman, E., Şen, Ş. & Şen, U. Dry Sliding Wear Behavior of Cr-Al-N Diffusion Coatings against Silicon Nitride. *J. Mater. Eng. Perform.* **30**, 2113–2123 (2021).

38. Qi, W. *et al.* Tribolayer-dependent origin of ultralow friction in nanocrystalline diamond films sliding against Si3N4 ball. *Surf. Interface Anal.* 1–14 (2021) doi:10.1002/sia.6994.

39. Korsunsky, A. M. Variational Eigenstrain Analysis of Synchrotron Diffraction Measurements of Residual Elastic Strain in a bent Titanium Alloy Bar. *J. Mech. Mater. Struct.* **1**, 259–277 (2006).

40. Brinckmann, S., Kirchlechner, C. & Dehm, G. Stress intensity factor dependence on anisotropy and geometry during micro-fracture experiments. *Scr. Mater.* **127**, 76–78 (2017).

41. Riedl, A. *et al.* A novel approach for determining fracture toughness of hard coatings on the micrometer scale. *Scr. Mater.* **67**, 708–711 (2012).

42. Best, J. P. *et al.* Small-scale fracture toughness of ceramic thin films: the effects of specimen geometry, ion beam notching and high temperature on chromium nitride toughness evaluation. *Philos. Mag.* **96**, 3552–3569 (2016).

43. Brinckmann, S., Matoy, K., Kirchlechner, C. & Dehm, G. On the influence of microcantilever pre-crack geometries on the apparent fracture toughness of brittle materials. *Acta Mater.* **136**, 281–287 (2017).

44. Tada, H., Paris, P. C. & Irwin, G. R. *The Stress Analysis of Cracks Handbook, Third Edition*. (ASME Press, 2000). doi:10.1115/1.801535.

45. Clausing, R. E., Heatherly, L. & More, K. L. Electron microscopy of the growth features and crystal structures of filament assisted CVD diamond films. *Surf. Coatings Technol.* **39**, 199210 (1989).

46. Clausing, R. E. *et al.* Textures and morphologies of chemical vapor deposited (CVD) diamond. *Diam. Relat. Mater.* **1**, 411–415 (1992).

47. Noyan, I. C.; Cohen, J. B. *Residual Stress: Measurements by Diffraction and Interpretation*. (Springer-Verlag, 1987).

48. McSkimin, H. J. & Andreatch, P. Elastic moduli of diamond as a function of pressure and temperature. *J. Appl. Phys.* **43**, 2944–2948 (1972).

49. Zeilinger, A. *et al.* In-situ Observation of Cross-Sectional Microstructural Changes and Stress Distributions in Fracturing TiN Thin Film during Nanoindentation. *Sci. Rep.* **6**, 22670 (2016).

50. Lassnig, A. *et al.* Nanoscale stress and microstructure gradients across a buckled Mo-Cu bilayer: Cu self-annealing triggered by interface delamination. *Acta Mater.* **283**, 120465 (2025).

51. Klein, C. A. & Cardinale, G. F. Young's modulus and Poisson's ratio of CVD diamond. *Diam. Relat. Mater.* **2**, 918–923 (1993).

52. Cinar, A. & Sinclair, G. B. Quasi-static normal indentation of an elasto-plastic half-space by a rigid circular cylinder of infinite length. *Int. J. Solids Struct.* **22**, 919–934 (1986).

53. Meyer, E. Untersuchungen über Härteprüfung und Härte. *Zeitschrit des Vereines Dtsch. Ingeneure* **52**, (1908).

54. Jun, T. S. & Korsunsky, A. M. Evaluation of residual stresses and strains using the Eigenstrain Reconstruction Method. *Int. J. Solids Struct.* **47**, 1678–1686 (2010).

55. Uzun, F. & Korsunsky, A. M. Voxel-Based Full-Field Eigenstrain Reconstruction of Residual Stresses. *Adv. Eng. Mater.* **25**, (2023).

56. Kachold, F. S., Lodes, M. A., Rosiwal, S. M. & Singer, R. F. Direct measurement of Young's modulus, fracture strength and fracture toughness of nanocrystalline diamond foil by means of tensile testing. *Acta Mater.* **61**, 7000–7008 (2013).

57. Hess, P. The mechanical properties of various chemical vapor deposition diamond structures compared to the ideal single crystal. *Journal of Applied Physics* vol. 111 (2012).
