# Supplementary material for: How interfaces limit nanoscale stress concentrations and prevent catastrophic failure in single-asperity contacts
Source: Commun Mater. 2026 Jun 30;7(1):194. doi: 10.1038/s43246-026-01239-1 (PMC13391362; doi:10.1038/s43246-026-01239-1)
Supplement: Supplementary file 1 — Transparent Peer Review file [file 43246_2026_1239_MOESM1_ESM.pdf]

nature portfolio

Peer Review File

How Interfaces Limit Nanoscale Stress Concentrations and Prevent Catastrophic Failure in Single-Asperity Contacts

Corresponding Author: Dr Michael Meindlhumer

This file contains all reviewer reports in order by version, followed by all author rebuttals in order by version.

Version 0:

Reviewer comments:

Reviewer #1

(Remarks to the Author)
In this manuscript, the authors report in-situ stress measurements using SAXS with 80 nm spatial resolution during indentation of a ZrN single layer (SL) and a ZrN-ZrCu multilayer (ML) film deposited on Si substrate with a nanomechanical diamond probe (NDP, single crystal diamond wedge indenter coated with nanocrystalline diamond film). A major difference from the authors' previous work is the use of NDP, which allows tracking of stresses (through changes in FWHM of the diffraction rings) in the indenter alongside the thin film. The key finding of the work is that the metallic glass ZrCu layers in the ML function as stress dissipators and reduce stress concentration, thereby preventing crack propagation across the ZrN-Si interface. The experiments are complemented with theoretical predictions and FEM simulations of stress evolution during indentation of the SL and ML films.

While the experimental methodology and results are interesting, several questions regarding the interpretation and conclusions need to be answered before the manuscript can be considered for publication.

1. How many SL and ML samples were tested under indentation (both in-situ and ex-situ)? Did cracks always originate at the surface (top layer) for the ML samples and at the Si/film interface for the SL samples?
2. Was the porosity distribution across the film thickness quantified for the SL and ML sample? In particular, was the porosity distribution in the ML samples uniform? Could the cracks have originated in the top layer of the ML sample because of increased porosity in that layer?
3. For the ML film, both sigma_yy and sigma_zz remain compressive across the entire film. So, what is responsible for the nucleation of cracks in this film.
4. It is mentioned that the ZrCu layer is ductile and helps with the redistribution of stresses and arrest of cracks in the top layer of the ML film. Notably, the ZrCu layer is only 20 nm thick, which is close to the critical thickness for formation of mature shear bands in metallic glasses. Did the authors study ML films where the ZrCu layer is significantly thicker (50 nm or higher)?
5. It is more accurate to say that the ZrCu layer is tough, rather than ductile. Ductile would imply ability to sustain significant tensile deformation, which is generally not true for metallic glasses.
6. In the supplementary note, it is mentioned that pores were more visible in the SL film compared to the ML film. On the other hand, the densification under the indenter load is more pronounced and extends across the entire thickness for the ML film. How can these two observations be reconciled?

Reviewer #2

(Remarks to the Author)
This manuscript presents an exceptionally novel and technically advanced study that combines state-of-the-art in situ synchrotron X-ray nanodiffraction with a custom nanoindentation approach to directly probe stress evolution at single-asperity contacts. The work demonstrates a very high level of experimental sophistication and provides rare insight into nanoscale stress distributions in multilayer thin films, which represents a significant advance in understanding mechanical behavior at buried interfaces. The combination of modern characterization tools with carefully designed multilayer architectures results in a dataset that is both rich and scientifically valuable. The study is particularly strong in demonstrating how interfaces influence stress localization and prevent catastrophic failure, which is of broad relevance to coatings, multilayers, and nanoscale tribology. Overall, the manuscript contains excellent science with clear potential impact and I recommend publication after the authors substantially improve the structure and clarity of the paper to better disseminate the important results.

1) The main concern is the overall structure and readability of the manuscript, which is currently very long and dense with information. Although only two samples are analyzed and compared, the amount of detail presented is extremely extensive and the key messages become difficult to follow. The Results section is divided into many subsections that partly overlap, and several parts contain substantial discussion rather than purely presenting results. The authors should consider either combining the Results and Discussion sections or clearly separating them so that the results are first presented systematically and the interpretation is provided later in a more focused discussion.
2) The logical flow of figures and their discussion needs improvement. Several figures are referenced out of sequence (for example, Fig. 6 is mentioned much earlier and explained later), which interrupts the narrative and makes it difficult for the reader to follow the progression of the results. Figures should be discussed in the order they appear.
3) Some figures could be streamlined or reorganized. In particular, Figures 4 and 5 could likely be combined, while the full image datasets may be moved to the Supplementary Information to improve the readability of the main text. Conversely, Figures 6 and 7 contain excellent results and could be used more effectively in the discussion to highlight the main scientific findings.
4) Conceptually, the manuscript appears to contain two strong but somewhat independent scientific contributions. One part focuses on the development and calibration of the nanoindentation tip as a nanomechanical probe combined with synchrotron nanodiffraction, while the other focuses on the mechanical response and stress evolution in the SL and ML films. The authors may consider either dividing the work into two separate manuscripts or restructuring the paper into clearly separated sections corresponding to these two themes.
5) In several places the manuscript mixes results with interpretation, and the large number of equations embedded within the text further reduces readability. While the theoretical treatment is valuable, it may be helpful to move some of the detailed derivations to the Supplementary Information and keep the main text focused on the essential physical interpretation.
6) Some specific technical points require clarification. For example, in lines 158–160 the authors claim that the growth of ZrN columnar grains is templated across the amorphous ZrCu interlayers, leading to continuously widening grains in the multilayer film. However, based on the presented images, it appears that primarily the surface roughness is replicated across the interlayers, rather than a clear crystallographic templating. The authors should clarify this interpretation and provide stronger evidence if crystallographic templating is indeed occurring.
7) In line 162, the term "in situ indentation" is used for the nanoindentation experiment; however, it would be useful for the authors to explain more clearly what is meant by this term in this context and how it differs from conventional nanoindentation experiments.
8) There are also some minor inconsistencies in figure descriptions, for example Fig. 3f is not mentioned in the figure caption, which should be corrected.
9) The conclusions section could be strengthened, as the key outcomes of the work are currently not summarized as clearly as they could be. Given the novelty and importance of the study, the authors should explicitly highlight the main scientific findings and their broader implications.

In summary, this manuscript contains excellent and highly novel science enabled by advanced experimental techniques, and it provides valuable insight into nanoscale stress evolution at interfaces. I strongly recommend publication; however, the authors should carefully restructure and streamline the manuscript to improve clarity, figure organization, and separation of results and discussion so that the significance of the work can be more effectively communicated.

Reviewer #3

(Remarks to the Author)
The manuscript "How Interfaces Limit Nanoscale Stress Concentrations and Prevent Catastrophic Failure in Single-Asperity Contacts" by Meindlhumer et al. discusses the creation of a nanomechanical diamond probe and its integration with in-situ synchrotron X-ray nanodiffraction to directly map multiaxial stress fields on both the indenter and thin-film sides of a single-asperity contact, demonstrating that ductile ZrCu interlayers in a ZrN–ZrCu multilayer architecture diffuse stresses, reduce the stored elastic energy by ≈30 % and suppress crack propagation relative to a monolithic ZrN film with the support of FEM simulations.
I recommend that the manuscript be resubmitted only after substantial (major)

revisions have been undertaken.

I recommend that the manuscript be resubmitted only after substantial (major) revisions have been undertaken.

1. Clarity and Writing Style
- The manuscript contains several long, information-dense sentences and typographical errors (e.g., "ceate" → "create"). Streamlining prose and a thorough proofreading pass are required to improve readability.
- Avoid redundancy; for instance, the paragraph describing micro-cantilever experiments can be condensed to a brief statement of the experimental outcome, referencing the four pertinent studies without extensive narrative.

2. Scope and Focus of the Introduction
- The introductory section spans numerous fields but does not clearly articulate the specific knowledge gap addressed by the present work. A
concise statement of the gap—namely, the lack of dual-sided stress analysis in single-asperity contacts—should be foregrounded.
- Lists of biomaterials with layered architectures and exhaustive reference compilations are unnecessary in this context. Restrict citations to the most directly relevant works to maintain focus.

3. Distinction from Prior Work
The manuscript currently does not differentiate this study from the authors' earlier investigations of synchrotron-based in-situ nanoindentation on thin and multilayer films. Explicitly emphasize that the novelty lies in simultaneous stress mapping on both indenter and film surfaces, enabling a comprehensive, dual-sided assessment.

4. Structural Organization and Signposting
The manuscript alternates between experimental results, modeling comparisons, and broader implications without clear transitions. Adopt a more hierarchical structure with explicit subsection headings to guide the reader through the logical progression of the work.

5. Methodological Scope
- The inclusion of cantilever-bending tests is not justified, given that comparable data exist in the literature. Provide a rationale for this
additional method or consider its removal to streamline the narrative.
- Figure 2 presently lacks scale bars on the individual panels; these should be incorporated directly into each image rather than solely in the caption.
- Font sizes and styles are inconsistent across figures (e.g., Fig. 3f).

6. Integration of Supplementary Material
Critical methodological details and key results are relegated to the supplementary information, forcing readers to toggle between documents.
Relocate essential experimental descriptions and primary data to the main text to ensure self-contained comprehension.

7. Numerical Modeling Strategy
Three analytical/numerical models (Hertzian, M'Ewen, and FE) are employed despite the FE model offering superior detail. Clarify the purpose of retaining the simpler models.

8. Role of Friction and Plasticity
- The coefficient of friction at the contact interface, a parameter that influences both stress distribution and energy dissipation, is neither measured nor discussed.
- Plastic deformation is mentioned only briefly. A more thorough characterization—e.g., post-mortem microscopy—would substantiate claims regarding energy dissipation mechanisms.

9. Symmetry-Based Error Evaluation
The inherent symmetry of the single-asperity contact permits an internal consistency check. Compare shear-stress fields on opposite sides of the interface to quantify experimental uncertainty and validate the analytical approach.

10. Finite-Element Implementation Details
- Specify the FE package employed, mesh density, convergence criteria, and boundary conditions. Detail the constitutive models
(elastic, plastic, hardening rules) and parameter values (e.g., yield stress, modulus) for both ZrN and ZrCu layers, as well as for the
nanocrystalline-diamond indenter. Some elastic parameters are in the supplementary materials.
- The current FE model treats the indenter as a rigid cylinder, neglecting possible plasticity in the nanocrystalline diamond coating. At a minimum, model the indenter as an elastic solid with a high yield strength to capture realistic stress transfer.

11. Experimental Configuration and Replicability
The custom indentation setup is described insufficiently for replication. Include schematics, dimensional drawings, load-cell specifications, and alignment procedures.

12. Figure Captions

The caption for Fig. 6 is overly brief and does not convey the experimental context or the key observations.

13. Probe Characterization
Mechanical properties of the nanocrystal line-diamond coating—elastic modulus, hardness, and any residual stresses—are not quantified, yet they critically influence stress transfer across the interface.

15. Citation Density
The introduction and discussion contain an extensive list of peripheral references that dilute the focus on the central contribution. Reduce citation overload by retaining only the most directly relevant studies.

16. Generalization to Biological Composites
Drawing parallels between the present findings and biological composites (nacre, bone) is conceptually appealing but speculative. Temper the
discussion of such analogies, and if possible, provide quantitative comparisons to substantiate the claim.

Version 1:

Reviewer comments:

Reviewer #1

(Remarks to the Author)
The authors have adequately addressed my questions from the first review.

Reviewer #3

(Remarks to the Author)
The authors are to be commended for engaging in constructive and insightful scientific discussions in response to the issues raised during the review process. However, given that all three reviewers identified concerns regarding the manuscript structure, and that this structure has remained largely unchanged, it is recommended to streamline the manuscript. Specifically, the focus should be narrowed to the central theme—namely, the indentation of multilayers—while peripheral content (such as beam-bending analyses, incomplete or partial numerical models, and comparisons to biological materials) should be removed. Such revisions would allow the manuscript to concentrate on its core contributions, while any omitted material could be more appropriately referenced through a thesis or related publications. The space gained through this reduction could then be used to transfer relevant content from the supplementary materials into the main text.

In addition, the presentation of figures requires improvement. The font sizes appear inconsistent and disproportionate; for example, the labels "SL" and "ML" at the top of figures are significantly larger than comparable labels within the layers, despite smaller fonts being sufficiently legible (as demonstrated in Figure 5). Furthermore, Figure 5 combines differing plotting styles (panels g and h), and the use of orange text on a green background reduces readability.

Finally, the treatment of plasticity warrants further clarification. Figure 5 indicates that the stresses in the ZrCu layers exceed the reported yield stress (1.8 GPa) in multiple instances. This suggests that plastic deformation should occur, with adjacent layers constraining this behavior and thereby generating shear stress waves within the plastic regime. Indeed, the finite element results in Figure 5 show pronounced shear stress waves in the plastic domain, exceeding those observed in the elastic regime. However, the experimental results do not reflect this distinction, instead showing similar shear stress wave behavior in both elastic and plastic domains. This discrepancy should be addressed and explained in greater detail.

Reviewer #4

(Remarks to the Author)

Open Access This Peer Review File is licensed under a Creative Commons Attribution 4.0 International License, which permits use, sharing, adaptation, distribution and reproduction in any medium or format, as long as you give appropriate credit to the original author(s) and the source, provide a link to the Creative Commons license, and indicate if changes were made.
In cases where reviewers are anonymous, credit should be given to 'Anonymous Referee' and the source.
The images or other third party material in this Peer Review File are included in the article's Creative Commons license, unless indicated otherwise in a credit line to the material. If material is not included in the article's Creative Commons license and your intended use is not permitted by statutory regulation or exceeds the permitted use, you will need to obtain permission directly from the copyright holder.
To view a copy of this license, visit https://creativecommons.org/licenses/by/4.0/

Answer to the Reviewer and List of Changes in the Revised Manuscript

This is the first revision of the manuscript entitled "How Interfaces Limit Nanoscale Stress Concentrations and Prevent Catastrophic Failure in Single-Asperity Contacts". The authors are very grateful to the three Reviewers for their valuable comments, suggestions, and critical remarks. They are highly appreciated because they give us an independent view on our work, point out errors and pitfalls, which we have not realized, and help us to formulate the manuscript in a more understandable way leading to an overall improved version of our manuscript. All Reviewers' comments and suggestions were carefully considered and adopted.

In the response to the reviewers below, we explain how we handled the Reviewers' comments point by point. For a better read- and traceability, our answers and explanations directly addressed to the reviewers are written in turquoise; the text copied in the manuscript – if still taken from the as-submitted version of the manuscript are in black; changes in the revised manuscript version are highlighted in yellow. Removed text is presented in the response as crossed text and is omitted from the revised manuscript.

Reviewer #1 (Remarks to the Author):

In this manuscript, the authors report in-situ stress measurements using SAXS with 80 nm spatial resolution during indentation of a ZrN single layer (SL) and a ZrN-ZrCu multilayer (ML) film deposited on Si substrate with a nanomechanical diamond probe (NDP, single crystal diamond wedge indenter coated with nanocrystalline diamond film). A major difference from the authors' previous work is the use of NDP, which allows tracking of stresses (through changes in FWHM of the diffraction rings) in the indenter alongside the thin film. The key finding of the work is that the metallic glass ZrCu layers in the ML function as stress dissipators and reduce stress concentration, thereby preventing crack propagation across the ZrN-Si interface. The experiments are complemented with theoretical predictions and FEM simulations of stress evolution during indentation of the SL and ML films.
While the experimental methodology and results are interesting, several questions regarding the interpretation and conclusions need to be answered before the manuscript can be considered for publication.
The authors thank Reviewer #1 for the positive judgement of our work. Below our response is outlined in detail.

1. How many SL and ML samples were tested under indentation (both in-situ and ex-situ)? Did cracks always originate at the surface (top layer) for the ML samples and at the Si/film interface for the SL samples?
The authors want to remind Reviewer #1 that the *in situ* synchrotron experiments performed within this study are ultra-high-fidelity experiments. 37.500 2D diffractograms are evaluated for each load step and transferred into colour-coded contour plots. Thus, a total of 450.000 2D diffractograms was evaluated for this study. No further samples were investigated *in situ*. Since the indenter system is permanently located at the ID13 beamline of the ESRF, additional *ex situ* analyses are not viable.
In order to prove the statistical relevance of our analyses, we added spherical nanoindentation to our research data. Six indents were carried out each on the SL and ML films and evaluated according to the methodology described by Leitner *et al*. (Refs. 84 and 85, https://doi.org/10.1002/adem.201600669, https://doi.org/10.1016/j.matdes.2018.03.003).

Additionally, cross-sections of the indents were prepared by focused ion beam milling and imaged by SEM. Keeping in mind that the fracture of individual features within the thin films itself is an inherently statistical process, no cracks were observed in neither the SL nor the ML thin film. However, fracture of the Si substrate was consistently observed as a consequence of the spherical nanoindentation, as well as the formation of steps at the ZrN/Si interface, which may be interpreted as precursors to fracture of the film itself. Additionally, a gradual flattening of the curvature across the ZrCu interlayers was seen in agreement with the data from the *in situ* experiments presented in Fig. 7. Thus, the following changes were made to the manuscript in Sec. 2.1, page 5, lines 145-156:

Results from laboratory micromechanical testing performed on the ZrN single-layer (further denoted as SL) and ZrN-ZrCu multi-layered (further denoted as ML) thin films are presented in Fig. 2a, showing the reduced modulus E_r and hardness H obtained from nanoindentation measurements using a Vickers indenter tip, as well as Young's modulus E, fracture stress σ_F, and fracture toughness K_{IC} measured via microcantilever bending. Additionally, the composite yield stress σ_y was evaluated by spherical nanoindentation (Fig. 2a). The results indicate that both films exhibit nearly identical mechanical properties, as a consequence of the averaging effect inherent to Vickers indentation or predominantly tensile loading conditions (Suppl. Note2, Fig. S3). On the other hand, spherical nanoindentation, which is intrinsically closer to the single-asperity contact, reveals highly different composite yield stresses (Fig. 2a). This is a first hint that the ML and SL films have significantly different mechanical response to realistic contact mechanical loading conditions.

Additionally, changes were made in Sec. 2.1, page 6, lines 177-182:

While individual fracture features, like those in Fig. 2c-f, are inherently statistical, postmortem SEM analysis of the indents produced by complementary spherical nanoindentation (Suppl. Note 2, Fig. S5) revealed a comparable overall failure behavior for both the SL and ML films. This observation confirms that the results obtained from the *in situ* X-ray nanodiffraction experiments are not incidental but reflect a physically meaningful and reproducible mechanical response.

Finally, changes were made in Sec. 3, page 22, lines 608-615:

In contrast to this, in the topmost ZrN layer of the ML film, cracks were observed (Fig. 2e) in a region, where exclusively compressive normal stresses are applied during loading and tensile residual stress is built up upon unloading (Sec. 2.2, Suppl. Note 4). Therefore, cracking in the topmost ZrN layer of the ML film may occur during tensile stress built-up upon unloading. Independent of the mechanical origin of the cracks in the film, their location within the films indicates the most critically stressed areas – the ZrN/Si interface and the surface in case of the SL and ML films, respectively.

Furthermore, the yield stress obtained by spherical nanoindentation was added to Fig. 2.

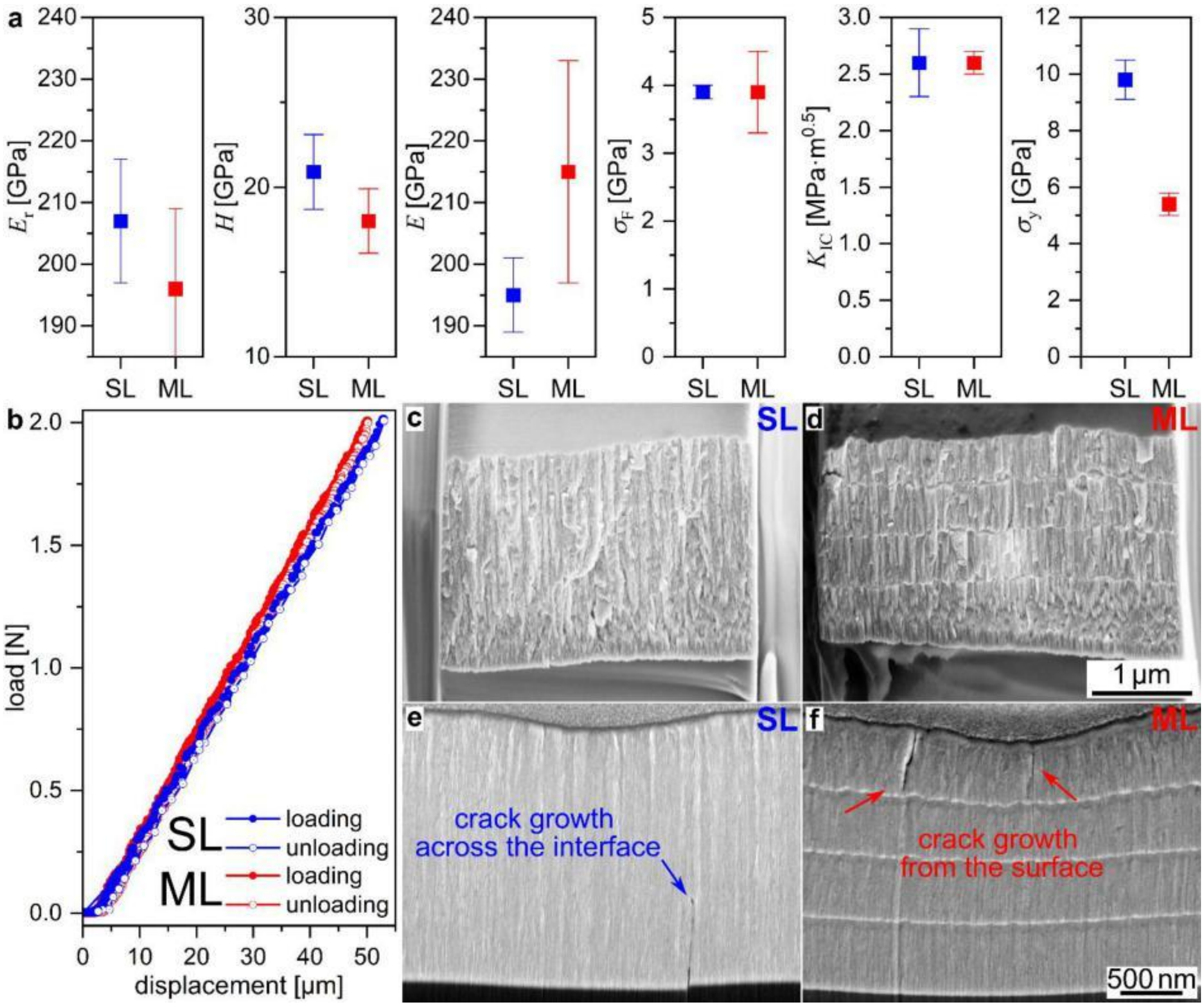

Figure 2. Results from micromechanical tests obtained from the monolithic (SL) and multilayered (ML) films. In (a), micromechanical properties of the films are presented, from left to right, the indentation modulus E_r, the nanoindentation hardness H, the elastic modulus E, fracture stress σ_F and fracture toughness K_{IC} obtained from the micromechanical bending tests, as well as the composite yield stress σ_y obtained by spherical nanoindentation. The load-displacement curves recorded from the *in situ* indentation experiments coupled with X-ray nanodiffraction (*cf.* Fig. 1) are presented in (b). In (c) and (d) fracture surfaces of unnotched microcantilevers are displayed, while in (e) and (f) the FIB-polished *post mortem* cross-sections from the center of the imprint are shown for SL and ML films, respectively. The scale bar in (d) is applicable to (c) and (d) while the scale bar in (f) is applicable to (e) and (f). Apparent deviations from a rectangular cross-section in (c) and (d) are due to the necessity to image these surfaces at an angle, thus they are insignificant.

The following changes were made to Sec. 4, Methods, FIB-SEM analysis on page 25, lines 732-733:

The same parameters were used for the preparations of the cross-sections of the spherical indents.

The following changes were made to Suppl. Note 2, pages 5-6 of the supplementary material:

Furthermore, spherical nanoindentation was performed with a sphero-conical indenter of 5 µm radius, to improve the statistical analysis of the microstructural response of the SL and ML thin films. Six indents were carried each out on both films, which yielded similar microstructural and mechanical responses. The results obtained by spherical nanoindentation using a tip with a radius similar to the nanomechanical diamond probe can be also interpreted as a single-asperity contact[75] and are presented in Fig. S5. First, the surface morphology of the indents performed on the SL and ML indents is presented in Fig. S5a and S5b, respectively, which is nearly identical for both thin films. Additionally, cross-sections were prepared from the indents on the SL and ML films by FIB milling and are presented in Fig. S5c and S5d, respectively. In the cross-sectional view, the difference in the mechanical response of the SL and ML films is highlighted by the different depth-dependent microstructural changes. In case of the SL film, the curvature imposed by the impinging indenter is nearly directly transferred to the substrate, which results in fracture of the Si substrate and subsequently a step at the interface (Fig. S5c). This behavior is similar to the microstructural response of the SL found after the indentation with the NDP (Fig. 2d, Fig. S2a). On the other hand, the ZrCu interlayer is apparently more flexible, and the curvature of the interlayers is gradually decreasing towards the substrate, effectively reducing the deformation imposed on the substrate (Fig. S5d). Finally, the stress-strain curves evaluated from the spherical nanoindentation are presented for the SL and ML films in Fig. S5e and S5f, respectively. Highly different stress-strain curves evaluated from the spherical indents, where yield stresses of 9.8±0.7 and 5.4±0.4 GPa were evaluated from the SL and ML films, respectively. While the composite yield stress of the ML film is significantly lower, after the onset of plastic deformation, a linear hardening is observed (Fig. S5f) and the observed stress at maximum indent depth is only slightly smaller compared to the SL film (Fig. S5e).

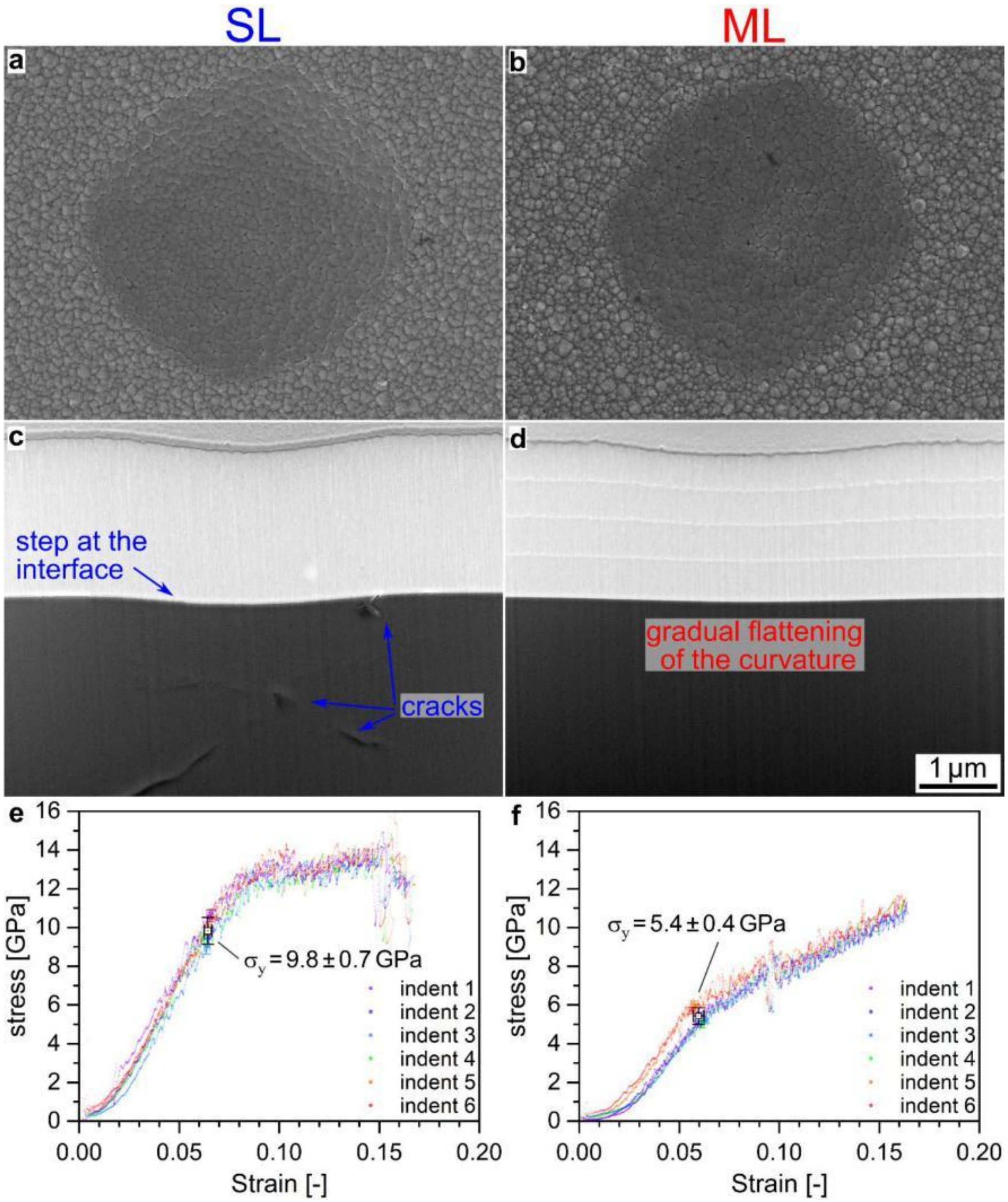

Figure S5. Data obtained from spherical nanoindentation on the SL and ML thin films. In (a) and (b), post indent SEM micrographs taken from the indents surface on the SL and ML films are shown, while in (c) and (d) FIB milled cross-section of the indents on the SL and ML films are presented, respectively. Stress-strain curves obtained from the spherical nanoindentation are presented in (e) and (f) for the SL and ML films, respectively. The scale bar in (d) equals 1 µm and is applicable to all images.

2. Was the porosity distribution across the film thickness quantified for the SL and ML sample? In particular, was the porosity distribution in the ML samples uniform? Could the cracks have originated in the top layer of the ML sample because of increased porosity in that layer?

Given that the main focus of our work lies in the characterization of stresses following the single-asperity contact and the different mechanical responses of the SL and ML films to it, we did not investigate the matter of porosity further. Generally, according to work dealing with structure zone diagrams and the fundamentals of formation of thin films, porosity increases with increasing film thickness during deposition in the Zone 1 regime (A. Anders,

https://doi.org/10.1016/j.tsf.2009.10.145, Barna and Adamik, https://doi.org/10.1016/S0040-6090(97)00503-8, Thornton, https://doi.org/10.1116/1.1312732). This is in good agreement with the electron microscopy images provided in Suppl. Note 1 (Fig. S1), where porosity was more clearly visible in the SL film and qualitatively increasing with film thickness. Additionally, regarding the question of Reviewer #1 targeting the fracture of the topmost ZrN layer in the ML film, it is unlikely that the cracks in the topmost ZrN layer in the ML film originate only from an increased porosity within that layer. We respectfully direct Reviewer #1 also to our response to the following question.

3. For the ML film, both sigma_yy and sigma_zz remain compressive across the entire film. So, what is responsible for the nucleation of cracks in this film.

We agree with Reviewer #1 that during loading, exclusively compressive normal stresses are applied close to the contact. However, cracks may also form under shear stresses, which are also present close to the contact. Additionally, there is the possibility of unloading cracks, since tensile horizontal stress (Fig. 6f) is present at the films surface after unloading. Due to the presence of comparably high tensile residual stress magnitudes after unloading, this is the most likely explanation and was adopted in the revised manuscript. Independent of the mechanical origin of the cracks, the location within the thin films coincide with the most critically stressed areas within the thin film. The following changes were made to the manuscript:

Sec. 2.1, page 6, lines 177-182:

While individual fracture features, like those in Fig. 2c-f, are inherently statistical, postmortem SEM analysis of the indents produced by complementary spherical nanoindentation (Suppl. Note 2, Fig. S5) revealed a comparable overall failure behavior for both the SL and ML films. This observation confirms that the results obtained from the *in situ* X-ray nanodiffraction experiments are not incidental but reflect a physically meaningful and reproducible mechanical response.

Additionally, changes were made in Sec. 3, page 22, lines 608-615:

In contrast to this, in the topmost ZrN layer of the ML film, cracks were observed (Fig. 2e), where exclusively compressive normal stresses are applied during loading and tensile residual stress is built up upon unloading (Sec, 2.2, Suppl. Note 4). Therefore, cracking in the topmost ZrN layer of the ML film may occur during tensile stress built-up upon unloading. Independent of the mechanical origin of the cracks in the film, their location within the films indicates the most critically stressed areas – the ZrN/Si interface and the surface in case of the SL and ML films, respectively.

4. It is mentioned that the ZrCu layer is ductile and helps with the redistribution of stresses and arrest of cracks in the top layer of the ML film. Notably, the ZrCu layer is only 20 nm thick, which is close to the critical thickness for formation of mature shear bands in metallic glasses. Did the authors study ML films where the ZrCu layer is significantly thicker (50 nm or higher)?

We agree with Reviewer #1 that additional microstructural features could lead to other interesting results. However, we want to point out again, that the *in situ* X-ray nanodiffraction experiments are of ultra-high fidelity and very time consuming in nature. In detail, a total of 225.000 2D diffractograms were recorded and evaluated per investigated sample, thus a total of 450.000 2D diffractograms were evaluated within this study. Therefore it is unfortunately not feasible that additional samples can be investigated ad-hoc by this *in situ* method. To still give some context, we respectfully direct Reviewer #1 to the work of Daniel *et al.* (Ref. 50, https://doi.org/10.1016/j.matdes.2025.114037), where several *other* multilayers composed of Zr(Cu)N and ZrCu were investigated using nanoindentation and micro-cantilever bending tests. The results of Daniel *et al*. show variations of hardness, elastic modulus, fracture stress and fracture toughness as a function of the number of interlayers and the interlayer thickness. Consequently, a reduction in hardness was accompanied by an increase of fracture toughness for all multilayered thin films investigated in that study. However, the ML film tested in this study was not included in the aforementioned publication. While practical reasons were relevant for the investigation of this specific film (*e.g.*: a 500 nm thick ZrN sublayer gives 6 unique voxels during scanning with 80 nm step size in cross-sectional direction), also the absence of any additional toughening mechanisms as shown in Fig. 2 was decisive and methodologically appealing to investigate the present ML film in this work.

5. It is more accurate to say that the ZrCu layer is tough, rather than ductile. Ductile would imply ability to sustain significant tensile deformation, which is generally not true for metallic glasses.

We thank Reviewer #1 for pointing out this vital distinction. We have removed "ductile" from the description of the ZrCu interlayers and replaced it with "tough" in agreement with Reviewer #1's suggestion at the following positions:

Page 4, lines 126-127:

The collected data provide a quantitative mechanistic explanation of how tough interlayers in multilayered architectures mitigate stress concentrations and fracture.

Page 23, lines 644-646:

Consequently, the ~30% of dissipated plastic work within the ZrCu (Fig. 7c), can be regarded as a toughening mechanism, reducing the overall stress buildup and thereby enhancing the failure tolerance.

Page 24, lines 671-672:

It was shown experimentally how *tough interlayers function as stress dissipators* during multi-axial loading.

6. In the supplementary note, it is mentioned that pores were more visible in the SL film compared to the ML film. On the other hand, the densification under the indenter load is more pronounced and extends across the entire thickness for the ML film. How can these two observations be reconciled?

We agree with Reviewer #1 that these two different observations are incoherent. However, upon the suggestion of Reviewer #1, we have investigated the SAXS intensities further and found

that the intensity changes differ significantly between the SL and ML thin film samples. While the area fraction, where intensity changes are observed are larger in the ML film, the absolute change of scattered intensity in the deformed volume is significantly higher in the SL film. The latter indicates that densification occurs to a higher degree – in agreement with the qualitatively higher number of pores detected by SEM. To reconcile our findings, the following changes have been made to the manuscript on page 7, lines 221-225:

On the contrary, the ML thin film shows a highly extended zone of slightly reduced SAXS intensity beneath the indenter tip (Fig. 4a), spanning over the entire film thickness, which indicates some densification due to the compressive stress state (Fig. 4). However, the markedly smaller intensity change indicates that this densification occurs to a significantly lesser extent compared to the SL film.

Reviewer #2 (Remarks to the Author):

This manuscript presents an exceptionally novel and technically advanced study that combines state-of-the-art in situ synchrotron X-ray nanodiffraction with a custom nanoindentation approach to directly probe stress evolution at single-asperity contacts. The work demonstrates a very high level of experimental sophistication and provides rare insight into nanoscale stress distributions in multilayer thin films, which represents a significant advance in understanding mechanical behavior at buried interfaces. The combination of modern characterization tools with carefully designed multilayer architectures results in a dataset that is both rich and scientifically valuable. The study is particularly strong in demonstrating how interfaces influence stress localization and prevent catastrophic failure, which is of broad relevance to coatings, multilayers, and nanoscale tribology. Overall, the manuscript contains excellent science with clear potential impact and I recommend publication after the authors substantially improve the structure and clarity of the paper to better disseminate the important results.

The authors very much appreciate the positive judgement of our work by Reviewer #2. Below our response is outlined in detail.

1) The main concern is the overall structure and readability of the manuscript, which is currently very long and dense with information. Although only two samples are analyzed and compared, the amount of detail presented is extremely extensive and the key messages become difficult to follow. The Results section is divided into many subsections that partly overlap, and several parts contain substantial discussion rather than purely presenting results. The authors should consider either combining the Results and Discussion sections or clearly separating them so that the results are first presented systematically and the interpretation is provided later in a more focused discussion.

According to the suggestion of Reviewer #2 we have restructured the Results and Discussion sections. First, we have combined the microstructural changes and the stress distributions evaluated from the *in situ* X-ray nanodiffraction data (Sec. 2.2 and Sec. 2.3 of the original submission), to reduce the number of subsections. Second, we have moved the section "*2.5 Implications of the different nanomechanical response of the SL and ML thin films"* and integrated it into the discussion. Thus, the progression of the subsections within the Results is now:

2.1 Laboratory micromechanical and electron microscopy analyses

2.2 In situ Synchrotron X-ray nanodiffraction Analysis of Microstructural Changes and Stress Distributions in the NDP, SL and ML Thin Films

2.3 Analytical and Numerical Recreation of the Experimentally Obtained Stresses in the NDP, SL and ML films

We strongly believe that the reorganization of the manuscript allows to follow the results and discussion sections in a more focused and structured way. The Sec. 2.5 of the original manuscript is now integrated into the discussion section of the revised manuscript from pages 18-20, lines 506-572 as follows:

For the second part of the discussion concerning the similarities and differences in the mechanical responses of the SL and the ML thin films, a direct comparison of several stress features throughout the indentation is presented in Fig. 6. As shown schematically in Fig. 6a, the vertical line profiles of σ_{yy} were extracted from the 2D stress data (Suppl Notes 3 and 4) at

the *y*-position of the center of the contact between the NDP and the SL as well as the ML, and are presented together in Fig. 6a-f for all loading steps. Before loading, the residual stress of the NDP is centered at ~0 GPa for both experiments, while the average residual stress before loading of both thin films is ~-2.4 GPa (*cf.* Fig. 6a). During the initial stages of loading (Figs. 6b and 6c corresponding to loads of 0.2 and 0.5 N, respectively) the general stress distribution is similar for diamond in contact with the SL and ML thin films, while the thin films' stress values themselves differ only by their initial residual stress levels (*cf.* Fig. 6a).

Generally, in addition to the residual stress in the SL and ML films, compressive and tensile stress are applied at the contact and the ZrN/Si interface throughout the loading, respectively. Additionally, oscillations of a_{yy} are present in the ML thin film (Fig. 6a, in detail Fig. S11 in Suppl. Note 4), whose magnitudes increase with increasing load and highlight the plastic limits of the ZrCu interlayer. Beginning with the loading up to 1.0 N, the gap between the a_{yy} in the diamond and the thin films reduces, which indicates irreversible deformation in the thin films close to the contact, after surpassing the yield stress of the thin films (Fig. 2a). While the onset of plastic deformation could be indirectly inferred from the von Mises stress surpassing the yield stress at a load of 1.0 N (Fig. S13, Fig. S9, Fig. 2a), only the NDP allows a direct comparison by assessing the stress magnitudes in both materials.

The stress gap across the interface vanishes at 2.0 N load (Fig. 6e), indicating high plastic deformation for both thin films close to the contact (in agreement with the regions of FWHM>>0.65 deg, discussed above). For the SL thin film indented by the NDP, a strong in-plane gradient of a_{yy} is also present, ranging from -9.3±0.3 at the contact with the probe to 0.2±0.2 GPa at the ZrN/Si interface under the maximum applied load of 2.0 N (Figs. 4c, 6e, 6g). However, these stress levels have to be considered with respect to the residual stress in the SL ZrN, that already existed before the loading. This corresponds to a stress change of – 6.3 ± 0.3 at the contact and +2.9 ± 0.3 GPa at the interface. Moreover, the tensile stress at the interface likely represents a lower-bound estimate, as a significant portion of the stress had already been relieved at the maximum load due to fracture of the Si and ZrN (Figs. 3a, 2e). On the other hand, the oscillations of a_{yy} in the ML thin film become even more pronounced, introducing a stress variation of up to 2 GPa in the individual ZrN sublayers, while the observed stress magnitudes range from -7.1 ± 0.3 GPa to -1.0 ± 0.2 GPa. Here, for both experiments the crucial influence of the substrate material is revealed: While sc diamond (*E*~1120 GPa[65]) is stiffer compared to nc diamond (E~460 GPa[51] by a factor of ~2.3, sc Si[73] (E~155GPa) is more compliant than ZrN (E~200 GPa, Fig. 2). Consequently, since Si is more compliant, a bending-

like deformation is induced in the ZrN by the NDP, while the stiff diamond substrate in the probe itself leads to a higher localization of elastic deformation in the nc diamond (Fig. 4).

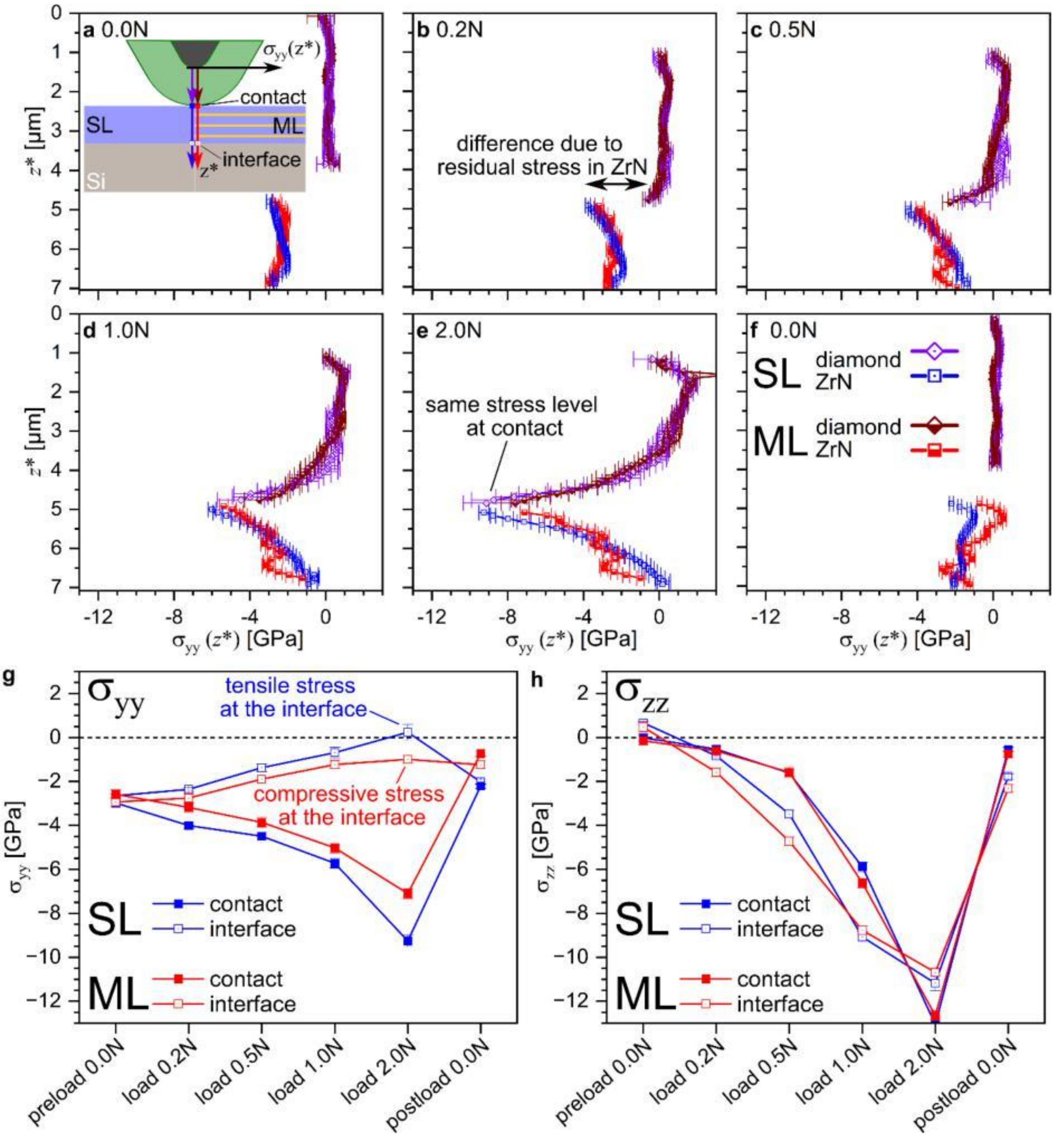

Figure 6. A detailed stress comparison of the SLand ML in contact with the NDP. A schematic of the individual analyses presented is shown in (a), representing the line intersections of the 2D stress fields presented in Fig. 4 in the center of the contact between the SL and ML films and the NDP. In (a), the σ_{yy} line profiles depicting the residual stress in the SL and ML as well as the NDP before the experiments. Note that the NDP is nearly stress-free before the experiment (a). The σ_{yy} line profiles across the indenter-sample interface are shown for the SL and the ML in contact with the NDP for the individual load steps of 0.2, 0.5, 1.0 and 2.0 N in (b), (c), (d) and (e), respectively, while the residual stress accumulation in the SL and ML films as well as the NDP after the experiment is presented in (f). The σ_{yy} and σ_{zz} stress values accumulated at the contact and the ZrN/Si interface are shown for the SL and the ML in (g) and (h), respectively, as shown in the schematic in (a).

Finally, after the unloading, the diamond returns exactly to the same stress state present before the experiments, proving that no irreversible deformation was present within the nc diamond

during both experiments (Fig. 6f). On the contrary, the σ_{yy} residual stress profiles in both the SL and ML thin films shift towards more tensile stress levels. However, the shift is less pronounced in the SL compared to the ML thin film, both at the ZrN/Si interface and the prior contact with the indenter (Fig. 6f). This indicates that the ability for inelastic deformation is comparatively limited in the SL thin film.

To further condense the comparison, the σ_{yy} and σ_{zz} stress values at the contact and the ZrN/Si interface are shown for the different loading states in the SL and the ML thin films in Fig. 6g and 6h, respectively, according to the schematic in Fig. 6a. While the σ_{zz} stress values are comparable throughout the experiments both at the contact with the NDP and the ZrN/Si interface (Fig. 6h), significant differences are found for the σ_{yy} stress component (Fig. 6g). During loading, both the accumulation of compressive and (more) tensile stress at the contact and the interface are less pronounced in the ML thin film in comparison with the SL thin film (Fig. 6g). This indicates that the ML thin film has a significantly reduced resistance against lateral deformation as a consequence of the ZrCu interlayers, which allows to accommodate the highest local loads without brittle failure across the ZrN/Si interface (Fig. 2).

2) The logical flow of figures and their discussion needs improvement. Several figures are referenced out of sequence (for example, Fig. 6 is mentioned much earlier and explained later), which interrupts the narrative and makes it difficult for the reader to follow the progression of the results. Figures should be discussed in the order they appear.

The authors are thankful to Reviewer #2 for pointing out the possible improvement regarding the logical flow of the figures. Any mention of figures out of order is removed from the revised manuscript.

3) Some figures could be streamlined or reorganized. In particular, Figures 4 and 5 could likely be combined, while the full image datasets may be moved to the Supplementary Information to improve the readability of the main text. Conversely, Figures 6 and 7 contain excellent results and could be used more effectively in the discussion to highlight the main scientific findings. We respectfully disagree with Reviewer #2 regarding streamlining the presentation of Figs. 4 and 5. The first reason for keeping both figures is, that Fig. 4 contains the experimental results of the NDP in contact with the SL and ML films. This allows the potential readers of the manuscript to immediately assess the stress distributions in the NDP, as well as the SL and ML films. Furthermore, Fig. 4 illustrates the inherent symmetry of the single-asperity contact, demonstrating the validity of the experimental approach. Secondly, especially after revising the FE model, which now includes the stress distributions obtained from the NDP, Fig. 5 is extremely rich with experimental, analytical and numerical data. Therefore, we genuinely believe that keeping Figs. 4 and 5 separate is in the best interest of the manuscript.

Additionally, according to the suggestion of Reviewer #2, we have strengthened the discussion by further focussing on Figures 6 and 7 during our discussion. As already mentioned above, we have removed Sec. 2.5 of the original manuscript from the results and integrated it into the discussion section on pages 18-20, lines 506-572 of the revised manuscript. Furthermore, we

have further integrated the two scientific parts of the manuscript, namely the introduction of the NDP and the nanomechanical response of the SL and ML films. Finally, we also considered the stored elastic energy in the NDP (Fig. 7c), which further connects the results obtained from the NDP and both the SL and ML thin films and strengthens our argumentation regarding energy dissipation in the ML film.

4) Conceptually, the manuscript appears to contain two strong but somewhat independent scientific contributions. One part focuses on the development and calibration of the nanoindentation tip as a nanomechanical probe combined with synchrotron nanodiffraction, while the other focuses on the mechanical response and stress evolution in the SL and ML films. The authors may consider either dividing the work into two separate manuscripts or restructuring the paper into clearly separated sections corresponding to these two themes.

We agree with Reviewer #2 that the manuscript contains two strong scientific contributions, but we would like to keep the two themes going along with each other, as their interpretation greatly benefits from the illustrative support received from the respective other. For instance, in many key aspects, the NDP, which is demonstratively only deformed elastically, acts as a calibrant for the interpretation of the results related to the different deformation behaviour of the SL and ML films. That only makes sense if they are presented together. However, in addition to the existing disclaimer at the beginning of the discussion, separating it in two parts, the second part is now further highlighted in Sec. 3, Discussion, page 18, lines 506-508:

For the second part of the discussion concerning the similarities and differences in the mechanical responses of the SL and the ML thin films, a direct comparison of several stress features throughout the indentation is presented in Fig. 6.

Furthermore, we specifically highlight the benefits of having the NDP for the interpretation of the stress results obtained from the SL and ML films in Sec. 3, Discussion, page 18, lines 523-526:

While the onset of plastic deformation could be indirectly inferred from the von Mises stress surpassing the yield stress at a load of 1.0 N (Fig. S13, Fig. S9, Fig. 2a), only the NDP allows a direct comparison by assessing the stress magnitudes in both materials.

Finally, to strengthen our arguments regarding the energy dissipation in the ML film, we have added the stored elastic energy of the NDP in contact with the SL and the ML thin film. Since the NDP is deformed exclusively elastically, the difference in stress magnitude at the contact has to be made up by the higher contact area to retain the force equilibrium. Therefore, the tip has to have the same stored elastic energy at the same applied force in contact with either the SL or the ML film. The, following change has been made in Sec. 3, Discussion, pages 22-23, lines 629-646:

Finally, the elastic strain energy stored in the NDP, SL and ML films is considered in Fig. 7c. Since the elastic strain energy is inversely proportional to the Young's modulus E (nc diamond: E≈460 GPa[60], ZrN: E≈200 GPa), the elastic strain energy in the NDP is ~2.3 times lower than in the ZrN (Fig. 7c). Given, that the stored elastic energy can be also expressed as $U = \int Pdx$, where P is the load and x is the displacement, similar strain energy densities can be estimated from the load-displacement data presented in Fig. 2d under the assumption of linear elasticity. That is actually the case for the NDP in contact with both the SL and ML films (*cf.* Fig. 7c).

The comparably lower stress magnitudes in the NDP in contact with the ML film (Figs. 4-6) are made up by the comparably higher contact area again as can be seen from Fig. 3, cross-validating the experimental observations drawn from the thin films. On the contrary, a lower stored strain energy is present in the ML film after loading beyond 0.2 N. In total, the strain energy in the ML film at 2.0 N is only ~69% of the strain energy stored in the SL. Since plastic deformation is highly limited in the nanoceramic ZrN in both films and only possible in very small volumes at the highest multi-axial load, this strain energy difference is safely interpreted as the dissipation of strain energy through plastic deformation of the ZrCu interlayer. Consequently, the ~30% of dissipated plastic work within the ZrCu (Fig. 7c), can be regarded as a toughening mechanism, reducing the overall stress buildup and thereby enhancing the failure tolerance.

Additionally, the following change has been made to the Suppl. Note 11, page 47:

The stored elastic energy per line element (in beam direction) E_{el} stored during deformation in the SL and ML samples was calculated from the $\sigma^{von\ Mises}_{ZrN}(y, z)$ at each load P stress distributions in analogy to Lassnig *et al.*[45] as follows

$$E_{el}^{P} = \sum_i \frac{1-\nu_i^2}{2E_i}\,\sigma^{von\ Mises}_{ZrN}(y, z)^2\,\Delta z \cdot \Delta y, \tag{S10}$$

where ν_i and E_i are the Young's modulus and Poisson's ratio of the investigated material, respectively, while Δy and Δz equal the step size of 80 nm used for the CSnanoXRD experiments. The elastic constants used in this study are summarized in Table S1, in Suppl. Note 13. In order to exclude the influence of residual stress present in ZrN before loading, the change of elastic energy is calculated by $\Delta E_{el}^{P} = E_{el}^{P} - E_{el}^{0N}$ for each load step. In case of the NDP, only the data up to a distance of 5 µm from the tip was included in the calculation to ensure a constant volume for the calculation of the strain energy.

Finally, the following changes have been made to Fig. 7c, introducing also the stored elastic energy of the NDP in contact with the SL and ML films:

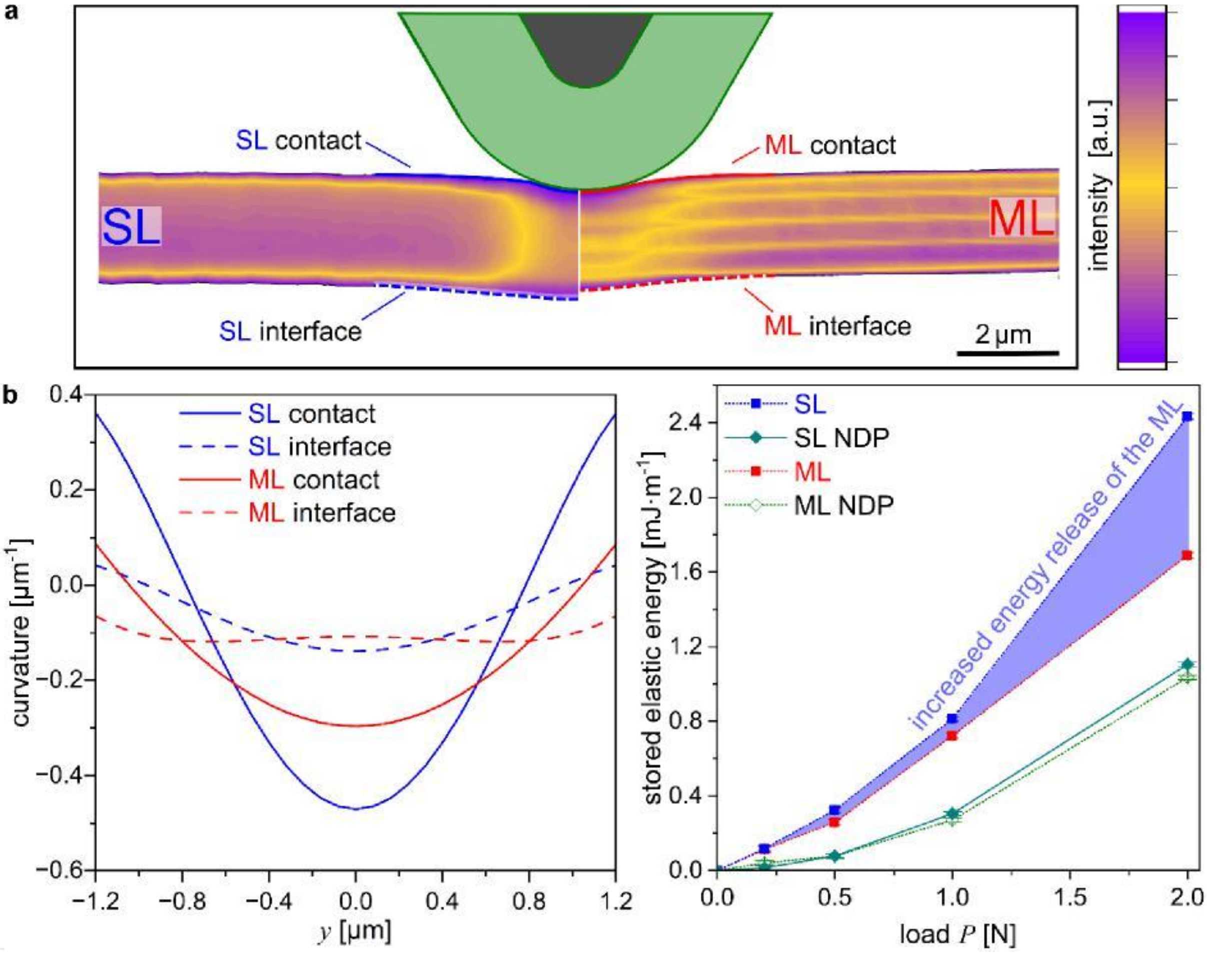

Figure 7. Geometrical response data and stored elastic energies within the SL and ML in contact with the NDP. In (a), the averaged intensities of the ZrN 111 DS ring retrieved from the SL and ML are presented together with a schematic of the NDP. The individual positions of curvature measurements for both films are also shown in (a), while the curvatures retrieved from the contact and the interface in the SL and ML films are presented in (b). The stored elastic energy per line element is shown for the NDP, the SL and the ML in (c), the difference represents the energy release of the ML film.

5) In several places the manuscript mixes results with interpretation, and the large number of equations embedded within the text further reduces readability. While the theoretical treatment is valuable, it may be helpful to move some of the detailed derivations to the Supplementary Information and keep the main text focused on the essential physical interpretation.

According to the suggestion of Reviewer #2, we have relegated the derivation of the contact hardness of the cylinder contact in the discussion to Suppl. Note 12. Thus, the number of equations embedded in the text is significantly reduced. That will help the reader to focus on the physical interpretation of the contact mechanical data. The following changes have been made: Sec. 3, Discussion, page 16, lines 446-456:

To interpret the collected multi-axial stress data evaluated from dual-side analysis of the single-asperity contact (Fig. 4-5), the hardness of the cylindrical contact[68] is considered , which is defined as $Hc \approx 2.24 \cdot \sigma_y$, in analogy to the Meyer hardness[69], which is nowadays accepted as a hardness criterion in indentation. This can be done by reversing the argument of Tabor and later works[70,71], where the yield stress σ_y is proportional to the indentation hardness Hi by a factor of 2.8-3. Therefore, the hardness of the cylindrical contact Hc should be $Hc \approx 0.75 \cdot Hi - 0.8 \cdot Hi$ ≈ 15.8 – 16.8 GPa, based on the measured nanoindentation hardness value of the SL film (Fig 2a). By using the applied load (P = 2.0 N), sample thickness (l = 56 µm) and the Hertz' contact width (2a = 2.66 µm), a $Hc = \frac{P}{l \cdot 2a}$ of ~13.4 GPa is obtained. Applying the Meyer

hardness directly to the cylindrical contact , the geometrically derived Hc value is in significantly better agreement with the experimental data ($|\sigma_{zz}|$, $\sigma_{von\ Mises} \approx$ 13 GPa), while Hc is closer to the analytical elastic contact solution (Fig. 5), where a contact pressure of 17 GPa was calculated. A full description of these derivations is presented in Suppl. Note 12.

The following text was added to Suppl. Note 12, page 49:

The hardness of the cylindrical contact[47] is defined in analogy to the Meyer hardness[48] as follows:

$$Hc \approx 2.24 \cdot \sigma_y, \quad \text{(S18)}$$

where σ_y is the yield stress of the indented material. Reversing the argument of Tabor and later works[16,17], where the indentation hardness Hi of a material is related to the yield strength by a factor of 2.8-3, the hardness of the cylindrical contact can be defined as follows:

$$Hc \approx 0.75 \cdot Hi - 0.8 \cdot Hi. \quad \text{(S19)}$$

Furthermore, the analogy to the Meyer hardness[48] can be driven further, using the sample thickness (l = 56 µm) in beam direction, the applied load (P = 2.0 N) and the Hertz' contact width (2a = 2.66 µm) to derive the contact hardness as follows:

$$Hc = \frac{P}{l \cdot 2a} \quad \text{(S19)}$$

Inserting the individual parameters, a contact hardness H_C of ~13.4 GPa is obtained from the experimental data.

6) Some specific technical points require clarification. For example, in lines 158–160 the authors claim that the growth of ZrN columnar grains is templated across the amorphous ZrCu interlayers, leading to continuously widening grains in the multilayer film. However, based on the presented images, it appears that primarily the surface roughness is replicated across the interlayers, rather than a clear crystallographic templating. The authors should clarify this interpretation and provide stronger evidence if crystallographic templating is indeed occurring.

We thank Reviewer #2 for this critical observation and agree with their interpretation of the SEM fracture surfaces. Therefore, we have removed the sentence dealing with crystallographic templating in Sec. 2.1.

Second, the growth of the ZrN columnar grains is templated across the amorphous ZrCu in the ML film (Fig. 2d, Fig. S4), leading to continuously widening ZrN grains with increasing film thickness, similar to the SL thin film

7) In line 162, the term "in situ indentation" is used for the nanoindentation experiment; however, it would be useful for the authors to explain more clearly what is meant by this term in this context and how it differs from conventional nanoindentation experiments.

We thank Reviewer #2 for this critical observation. Of course, "*in situ* indentation" refers to the *in situ* indentation experiment coupled with X-ray nanodiffraction, which is *in situ* from the perspective of the nanodiffraction. Therefore, according to the suggestion of Reviewer #2, we specified "*in situ* indentation" further as "*in situ* indentation coupled with X-ray nanodiffraction" throughout the manuscript. Following changes have been made:

Page 5, lines 167-169:

Load-displacement data obtained from the *in situ* indentation experiments coupled with X-ray nanodiffraction using the nanomechanical diamond probe (further denoted as NDP, Fig. 1) on the SL and ML films are presented in Fig. 2b.

Page 6, lines 187-197:

Figure 2. Results from micromechanical tests obtained from the monolithic (SL) and multilayered (ML) films. In (a), micromechanical properties of the films are presented, from left to right, the indentation modulus E_r, the nanoindentation hardness H, the elastic modulus E, fracture stress σ_F and fracture toughness K_{IC} obtained from the micromechanical bending tests, as well as the composite yield stress obtained by spherical nanoindentation. The load-displacement curves recorded from the *in situ* indentation experiments coupled with X-ray nanodiffraction (*cf.* Fig. 1) are presented in (b). In (c) and (d) fracture surfaces of unnotched microcantilevers are displayed, while in (e) and (f) the FIB-polished *post mortem* cross-sections from the center of the imprint are shown for SL and ML films, respectively. The scale bar in (d) is applicable to (c) and (d) while the scale bar in (f) is applicable to (e) and (f). Apparent deviations from a rectangular cross-section in (c) and (d) are due to the necessity to image these surfaces at an angle, thus they are insignificant.

Page 11, lines 318-322:

Figure 4. CSnanoXRD data from the NDP, SL and ML films obtained during *in situ* indentation experiments coupled with X-ray nanodiffraction at a load of 2.0 N. The σ_{yy}, σ_{zz} and σ_{yz} components of the stress tensor are presented in (a) and (b), (c) and (d) and (e) and (f) for the ML and SL in contact

with the NDP, respectively. Additionally, in (g) and (h) the $\sigma_{\text{von Mises}}$ calculated from the stress components are displayed for the experiments involving the SL and ML, respectively.

Page 12, lines 330-332:

This σ_{yz} stress level suggests a significant influence of the metallic glass on the deformation response of the ML film during the *in situ* indentation experiment coupled with X-ray nanodiffraction.

8) There are also some minor inconsistencies in figure descriptions, for example Fig. 3f is not mentioned in the figure caption, which should be corrected.

The authors thank Reviewer #2 for observing this error. Fig. 3f is now mentioned in the figure caption as follows:

Figure 3. Microstructural changes in the SL and ML as well as in the nanocrystalline tip probe at a load of 2.0 N. In (a) and (b) the SAXSM micrographs obtained from the SL and ML are shown. The highlighted region in (a) represents a step in the Si/ZrN interface. In (c) and (d) micrographs depicting the azimuthally averaged FWHM are presented, respectively. The dotted black line in (c) and (d) represents a FWHM of ~0.65 deg. The schematic experimental setup (e) is shown alongside the progressive evolution of the FWHM interaction *width* and *depth* during incremental indenter loading presented in (f).

9) The conclusions section could be strengthened, as the key outcomes of the work are currently not summarized as clearly as they could be. Given the novelty and importance of the study, the authors should explicitly highlight the main scientific findings and their broader implications.

According to the suggestion of Reviewer #2 we have amended the final paragraph of the discussion containing the summary and conclusions from our work. We specifically added (i) the dual-sided stress analysis in the single-asperity contact, (ii) that the influence of the various interfaces could be identified and quantified, as well as (iii) that 30% of the elastic energy stored in the SL are dissipated during deformation of the ML. The following changes were made on pages 23-24, lines 665-680:

In summary, the approach presented here allowed us to quantify the mechanical and structural changes, including the multiaxial nanoscale dual-sided stress analysis in the single-asperity contact. Contrary to prior works, the nc diamond thin film on top of the sc diamond indenter tip enabled monitoring the mechanical response of both the indenter tip and the indented thin films within a single experiment with 80 nm resolution in bulk thin films. The influence of the various interfaces on the stress distribution was quantified and could be related to the elastic mismatch of the individual constituents. Additionally, it was shown experimentally how *tough interlayers function as stress dissipators* during multi-axial loading. Furthermore, this work demonstrates the mechanical and structural superiority of multi-layered soft–hard architectures, being able to dissipate 30% of the stored elastic energy compared to their monolithic counterparts, which might have been dismissed as inferior based solely on conventional laboratory mechanical characterization. The ML ZrN–ZrCu thin film effectively mitigates stress concentrations

induced by the imprinting indenter through plastic deformation of the comparatively softer ZrCu interlayers, thereby preventing crack propagation across the ZrN/Si interface. These findings have implications not only for the design of protective thin films but also provide a mechanistic understanding of the protective function of hierarchical biological composites.

In summary, this manuscript contains excellent and highly novel science enabled by advanced experimental techniques, and it provides valuable insight into nanoscale stress evolution at interfaces. I strongly recommend publication; however, the authors should carefully restructure and streamline the manuscript to improve clarity, figure organization, and separation of results and discussion so that the significance of the work can be more effectively communicated.
Again, the authors appreciate the positive judgement of our work by Reviewer #2 very much and are thankful for their critical appraisal of our work.

Reviewer #3 (Remarks to the Author):

The manuscript "How Interfaces Limit Nanoscale Stress Concentrations and Prevent Catastrophic Failure in Single-Asperity Contacts" by Meindlhumer et al. discusses the creation of a nanomechanical diamond probe and its integration with in-situ synchrotron X-ray nanodiffraction to directly map multiaxial stress fields on both the indenter and thin-film sides of a single-asperity contact, demonstrating that ductile ZrCu interlayers in a ZrN–ZrCu multilayer architecture diffuse stresses, reduce the stored elastic energy by ≈30 % and suppress crack propagation relative to a monolithic ZrN film with the support of FEM simulations.

I recommend that the manuscript be resubmitted only after substantial (major) revisions have been undertaken.

The authors thank Reviewer #3 for the critical assessment of our work.

1. Clarity and Writing Style

- The manuscript contains several long, information-dense sentences and typographical errors (e.g., "ceate" → "create"). Streamlining prose and a thorough proofreading pass are required to improve readability.

We thank Reviewer #3 for their detailed insight. We have performed a thorough proof-reading pass to eliminate all typographical errors. Furthermore, we have revised several long sentences, to improve the readability.

- Avoid redundancy; for instance, the paragraph describing micro-cantilever experiments can be condensed to a brief statement of the experimental outcome, referencing the four pertinent studies without extensive narrative.

We thank Reviewer #3 for this critical insight. According to the suggestion of Reviewer #3, we have reduced the narrative describing microcantilever bending experiments and the citation density. The following changes have been made in Sec. 1, pages 2-3, lines 81-88:

To quantify the impact of the thin film design strategies outlined above, the state of the art methods for determining the average micro-mechanical properties of advanced thin films include analysis by nanoindentation, micropillar compression[25,28,29] and *in situ* microcantilever bending tests in the [SEM17,21,22,35.] For the latter method, by testing both unnotched and notched cantilevers, Young's modulus, fracture stress and fracture toughness, respectively, can be extracted from the experiments. Nevertheless, despite showing improvement in fracture resistance, the vast majority protective nanoceramic thin films, even those with the most advanced architectural designs, still exhibit brittle, linear-elastic failure upon crack initiation under tensile loading due to their ceramic nature[17,21,22,35] (with only a few notable exceptions[22,44]).

2. Scope and Focus of the Introduction

- The introductory section spans numerous fields but does not clearly articulate the specific knowledge gap addressed by the present work. A concise statement of the gap—namely, the lack of dual-sided stress analysis in single-asperity contacts—should be foregrounded.

According to the suggestion of Reviewer #3, we have drawn additional focus to the dual-sided stress analysis of single-asperity contacts in the introduction. The following changes have been made in Sec. 1on page 3, lines 109-113:

Due to the limitation of the X-ray nanodiffraction approach to polycrystalline materials, the complementary response of the single-crystalline (sc) diamond indenter tip in these experiments has remained inaccessible[54–58]. Therefore, a dual sided experimental analysis of the single-asperity contact has not been possible up to now, leaving a crucial gap in the existing knowledge.

And further changes have been made in Sec. 1 on page 4, lines 124-126:

This novel approach enables, for the first time, the quantification of evolving multi-axial stress distributions in both the indenter tip and the indented material with unprecedented spatial resolution. Thus, a dual-sided stress analysis of the single-asperity contact is enabled, in addition to the evaluation of morphological and microstructural changes across the contact area.

- Lists of biomaterials with layered architectures and exhaustive reference compilations are unnecessary in this context. Restrict citations to the most directly relevant works to maintain focus.

According to the demand of Reviewer #3, we have removed the context of biomimetic materials in the introduction. The following sentence has been removed from the revised manuscript: Such thin ~~film microstructures are sometimes referred to as biomimetic[34,35], mimicking the architecture of biological structural materials[36] such as nacre[36–39], the dentine enamel junction[40], cortical bone[37,39] or other natural materials[41–43].~~

3. Distinction from Prior Work

The manuscript currently does not differentiate this study from the authors' earlier on investigations of synchrotron-based in-situ nanoindentation thin and multilayer films. Explicitly emphasize that the novelty lies in simultaneous stress mapping on both indenter and film surfaces, enabling a comprehensive, dual-sided assessment.

Following the comment 2 of Reviewer #3, in order to draw focus of the reader to the stress analysis of the single-asperity contact, we have added already amended the introduction of the manuscript. Additionally, we have explicitly specified the novelty of the dual-side approach at the beginning of the discussion section, page 16, lines 440-442:

First, we examine the implications of converting the indentation tip into a nanomechanical probe, a feat unattainable in previous experimental approaches using sc diamond indenter tips[45–49].

Furthermore, the reader is reminded that both the indenter tip and the indented thin films are investigated on page 16, lines 446-448:

To interpret the collected multi-axial stress data evaluated from dual-side analysis of the single-asperity contact (Fig. 4-5), the hardness of the cylindrical contact[59] is considered in analogy to the Meyer hardness[60], which is nowadays accepted as a hardness criterion in indentation.

Finally, we also added this distinction from prior work also to the end of the discussion section on page 23, lines 665-667:

In summary, the approach presented here allowed us to quantify the mechanical and structural changes, including the multiaxial nanoscale dual-sided stress analysis in the single-asperity contact. Contrary to prior works, the nc diamond thin film on top of the sc diamond indenter tip enabled monitoring the mechanical response of both the indenter tip and the indented thin films within a single experiment with 80 nm resolution in bulk thin films.

However, in addition to the analysis of the single-asperity contact, our analysis revealed the dissipation of the stress concentrations within the brittle-tough multilayer quantitatively for the first time. This is the second novelty presented within our work.

2. Structural Organization and Signposting

The manuscript alternates between experimental results, modeling comparisons, and broader implications without clear transitions. Adopt a more hierarchical structure with explicit subsection headings to guide the reader through the logical progression of the work.

According to the suggestions of Reviewer #2 and #3 we have restructured the Results and Discussion sections. First, we have combined the microstructural changes and the stress distributions evaluated from the *in situ* X-ray nanodiffraction data, to reduce the number of subsections. Second, we have moved the section "*2.5 Implications of the different nanomechanical response of the SL and ML thin films*" and integrated it into the discussion. Thus, the progression of the subsections within the Results is now:

2.1 Laboratory micromechanical and electron microscopy analyses

2.2 In situ Synchrotron X-ray nanodiffraction Analysis of Microstructural Changes and Stress Distributions in the NDP, SL and ML Thin Films

2.3 Analytical and Numerical Recreation of the Experimentally Obtained Stresses in the NDP, SL and ML films

We strongly believe that the reorganization of the manuscript allows us to follow the results and discussion sections in a more focused and structured way. Sec. 2.5 of the original manuscript is now integrated into the discussion section of the revised manuscript from pages 18-20, lines 506-572. Thereby, a more logical progression in following the work was achieved.

3. Methodological Scope

- The inclusion of cantilever-bending tests is not justified, given that comparable data exist in the literature. Provide a rationale for this additional method or consider its removal to streamline the narrative.

We agree with Reviewer #3 that the results of microcantilever bending tests performed on nanoceramic thin films are widely reported in the existing scientific literature. However, to the

best of our knowledge, *no study* has been published on the exact *same materials* investigated in this work, which justifies their inclusion here. Furthermore, as outlined in the introduction, nanoindentation and microcantilever bending tests are among the *most prominent laboratory techniques* for characterizing the mechanical properties of nanoceramic thin films (see, e.g., the recent review by P. Mayrhofer *et al*., Ref. 17, https://doi.org/10.1016/j.pmatsci.2024.101323). However, based on the laboratory characterization of the ML and SL thin films presented in Fig. 2a, no significant differences in mechanical properties between the investigated films were identified using either method. This absence of differences between the ML and SL samples in state-of-the-art laboratory characterization contrasts with the postmortem SEM investigation shown in Fig. 2, where a completely different failure behavior was observed. Therefore, the current limitations of micromechanical testing in the laboratory (Sec. 2.1) become evident when contrasted with the pronounced differences between the thin films revealed by *in situ* X-ray nanodiffraction (Sec. 2.2) as a characterization tool.
This is the rationale for including the microcantilever bending tests already present within the original manuscript: to provide a complete state-of-the-art laboratory characterization of both the ML and SL thin films as a benchmark against the *in situ* X-ray nanodiffraction methodology.

- Figure 2 presently lacks scale bars on the individual panels; these should be incorporated directly into each image rather than solely in the caption.
We thank Reviewer #3 for this suggestion. The values of the scale bar labels have been added to Fig. 2 in accordance with the advice of Reviewer #3. However, when panels share the same resolution, we believe that including a scale bar in each individual panel is not necessary.

- Font sizes and styles are inconsistent across figures (e.g., Fig. 3f).
The styles within the figures have been standardized across the entire manuscript in accordance with Reviewer #3's request. However, certain elements of signposting—such as the use of different colors or font sizes—have been intentionally retained at the authors' discretion to improve clarity and visual guidance within the figures.

6. Integration of Supplementary Material
Critical methodological details and key results are relegated to the supplementary information, forcing readers to toggle between documents. Relocate essential experimental descriptions and primary data to the main text to ensure self-contained comprehension.
We thank Reviewer #3 for highlighting that additional methodological details could facilitate manuscript readability. At the same time, Reviewer #2 suggested shortening the manuscript and relocating some content to the supplementary material. To reconcile these perspectives, we have included in the main text only the methodological information essential for understanding the results, while more detailed procedures—particularly those previously reported in our earlier publications—are provided in the supplementary material.
We believe this approach aligns with the scope of Communications Materials as a multidisciplinary journal with a broad readership, where the focus should remain on the physical interpretation of the observed mechanical phenomena. While the manuscript presents methodology that extends beyond the state of the art, the core X-ray nanodiffraction techniques have been outlined in prior works (Ref. 45-48, 88, 91). We have, however, added further methodological clarifications in response to Reviewer #3's comments to ensure no essential information is omitted. We believe, readers interested in the full technical details will make the effort to find those in the supplementary material, while all essential information is included in the main text.

7. Numerical Modeling Strategy

Three analytical/numerical models (Hertzian, M'Ewen, and FE) are employed despite the FE model offering superior detail. Clarify the purpose of retaining the simpler models.

We agree with Reviewer #3 that the finite element model gives deeper insight in case of recreating the experimentally observed deformation and the stress response of the ML film. However, as shown in the manuscript, for a contact where between two ceramic materials, plasticity is limited to regions of extremely high compressive stress close to the contact. Here, the analytical solution provided in the manuscript has value in describing the stress distributions. While the contact-mechanical approach provided by Hertz yields the contact half width a and the contact pressure p_0, the work by M'Ewen allows to identify the 2D distribution of stresses following the cylindrical contact at the same half width a and contact pressure p_0. This is explicitly stated in the original manuscript. Therefore, we want to remind Reviewer #3 that only one analytical model was used for the analytical description of the stress distribution. Since the analytical treatment by M'Ewen agrees reasonably well with the experimental data in the simple case of the SL film and does not need any significant computational power, we believe it to be a sustainable approach to treat the contact mechanical problem in a simplified manner, which clearly has some value in itself. To clarify this to the reader, following changes were made to the discussion section on page 18, lines 503-505:

Following these arguments, the analytical treatment by M'Ewen can be effectively used to easily assess the cylindrical contact of ceramic materials with limited plastic deformation, without the need for elaborate FE models.

8. Role of Friction and Plasticity

- The coefficient of friction at the contact interface, a parameter that influences both stress distribution and energy dissipation, is neither measured nor discussed.

We agree with Reviewer #3 that friction in principle be important. However, the effect of friction and tangential traction was already discussed in literature by Huang *et al.* (https://doi.org/10.1016/j.compscitech.2006.10.005), Liu et al. (https://doi.org/10.1016/j.jmps.2005.07.003) and Wang et al. (https://doi.lorg/10.1016/j.actamat.2004.01.016) using both experimental and numerical data. All these studies consistently agree that friction changes the stress distribution in the indented material only marginally and is therefore negligible. For sake of completeness, we have repeated the finite element models using friction coefficients μ of 0, 0.5 and 1.0 and found similarly that the stress distributions obtained from the contact of the NDP with the ML film change noticeably only at the highest and unrealistic friction coefficient (μ=1.0). Regarding the details of the newly employed FE models we respectfully refer Reviewer #3 to our reply to their comment #10.

Additionally, Johnson (Ref. 57, https://doi.org/10.1017/CBO9781139171731) provides an analytical solution for the tangential traction $q(x)$ without slip as a function of the contact pressure p_0 and the contact width a (Chapter 5.4, page 119 and following). This analytical solution is depicted as follows

$$q(x) = \frac{\beta p_0}{\pi a}\left[(a^2 - x^2)^{0.5} \ln\left(\frac{a+x}{a-x}\right) + x \ln\left(\frac{??+(a^2-x^2)^{0.5}}{a-(a^2-x^2)^{0.5}}\right)\right], \quad \text{(R1)}$$

where β is Dundurs' second parameter (https://doi.org/10.1177/002199836700100306). Using the elastic parameters of the investigated However, the tangential traction is limited by slip in

such way that $|q(x)| \leq \mu p(x)$, where μ is the friction coefficient and $p = $ 2?? $\pi l a (a - x^2)^{0.5}$ is the distribution of the normal pressure. Further investigations by Spence (https://doi.org/10.1007/BF00126993) could quantify that slip occurs outside x>0.7a in the case that $^{\beta}\sim 0.99$. In the present case, using the elastic constants provided in the article, $\beta \sim -0.2$.
μ

Assuming now a friction coefficient of 0.2, following normal and tangential traction distributions are evaluated within the contact area:

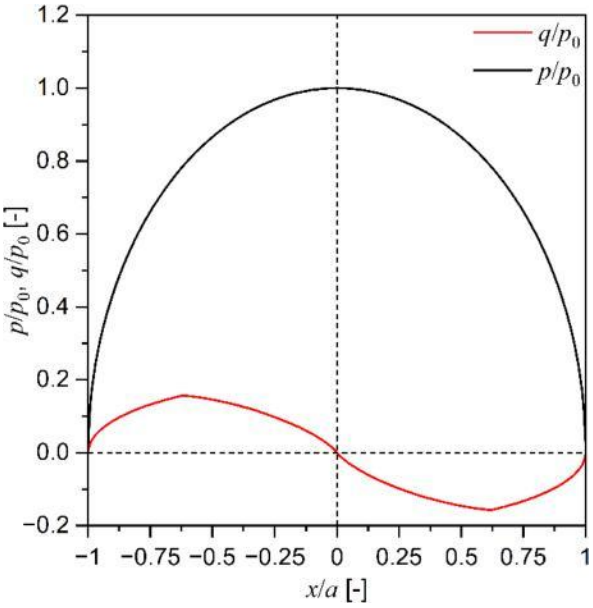

Fig. R1: Lateral distribution of the normalized tangential traction q/p_0 (red) and normal traction p/p_0 (black).

As one can see, the normalized tangential traction never exceeds $0.15p_0$. Furthermore, nanocrystalline diamond is well known for its ultra-low friction coefficient against other materials, which is typically in the order of 0.05 against various counter bodies (e.g. cemented carbide, ball bearing steels, stainless steel). To the best knowledge of the authors, data from tribological experiments with using ZrN and nc diamond as tribological pairing are not yet published, however, the friction coefficient between CrAlN (another transition metal nitride) and Si_3N_4 at 2 N load was found in the order of 0.05 (https://doi.org/10.1007/s11665-021-05498-6). Finally, friction coefficients of diamond against steel, ZrO_2 and Si_3N_4 have been measured by Qi *et al*. (https://doi.org/10.1002/sia.6994), where for the latter two pairings a friction coefficient of ~0.08 was found. These findings, together with the considerations of Johnson lead to the conclusion that the tangential traction is at maximum $|q\ (x)| \leq 0.1p\ (x)$ and can therefore be neglected. However, to draw the attention of the potential audience and to treat the mattter carefully, the following changes have been made to the manuscript:

Sec. 2.3, page 12, lines 350-354:

Please note that the complete datasets of the FE model including a parametric study with varying friction coefficients is present in Suppl. Note 7. However, since no significant influence of interfacial friction was observed, the numerical data of the frictionless contact between the NDP and the ML film are presented and compared with the experimental data of the ML film Fig. 5b, 5d and 5e.

Sec. 3, pages 17-18, lines 493-503:

Additionally, The FE model presented in Fig. 5 is in excellent agreement with the experimental data, solidifying our interpretation of the experimental stress distributions. Following another consideration, when two cylinders of dissimilar materials are in contact, the applied normal pressure leads to an additional tangential traction acting along the contact surfaces[65], which is limited by the normal pressure times the coefficient of friction of the paired materials[65]. Nanocrystalline diamond is well known for its low friction coefficient when paired with nearly any other material, where typical friction coefficients are below[78–80], or in the order of ~0.1. Furthermore, since the σ_{yy} magnitudes in the NDP obtained by the FE element model are nearly independent of the friction coefficient at the NDP/ML interface (*cf.* Suppl. Note 7), it is implied that tangential traction is not responsible for the tensile σ_{yy} found in the NDP.

- Plastic deformation is mentioned only briefly. A more thorough characterization—e.g., post-mortem microscopy—would substantiate claims regarding energy dissipation mechanisms.

We agree with Reviewer #3 that plastic deformation is not discussed extensively. However, the submission deals with (mostly) ceramic materials, where plastic deformation is only possible under (very) large compressive stresses. Even at the highest load of 2 N, the diamond is deformed exclusively elastically, since a hardness of 61.6±6.1 GPa was measured, which equals a yield stress of 20.5-22 GPa (the characterization of hardness is detailed in our reply to point 13 raised by Reviewer #3).

Regarding plasticity in the thin films, the experimental stress results are already supported by *postmortem* scanning electron microscopy (Fig. 2, Fig. S2) and *in situ* small-angle X-ray scattering microscopy (SAXSM) as well as *in situ* FWHM microscopy, which give additional qualitative insight into the deformation behaviour within the thin films. The observed microstructural changes include compaction and defect accumulation, indicated by decrease and increase of SAXS intensity and FWHM, respectively (Fig. 3). This change is permanent and can be also observed after the experiment, as can be seen for the SL and ML films in Fig. S11 and S17, respectively.

However, we respectfully, but sternly disagree that the observed energy dissipation is just a claim. As already discussed in the original manuscript, the SL and ML films differ by three 20 nm thick layers of ZrCu. Therefore, for a thin film with 2 µm thickness, the volumetric differences between the thin films are negligible (20 nm of ZrCu are equal to 1% of the film's thickness). However, since the SL and ML films in contact with the NDP are loaded to the same loads, and the NDP is loaded elastically, we have added the stored elastic energy of the NDP in contact with the SL and the ML thin film. Consequently, the difference in stress magnitude at the contact (Figs. 4-6) has to be made up by the higher contact area to retain the force equilibrium in the NDP. Therefore, the tip has to have the same stored elastic energy at the same applied force in contact with either the SL or the ML film. Reversing this argument, the difference in stored elastic energy observed for the ML and SL films has to be dissipated by plastic deformation in the 20 nm thick ZrCu interlayers. The three ZrCu interlayers are the only substantial morphological difference between the SL and ML films are. Again, therefore, these interlayers have to be responsible for the difference in stored elastic energy. Consequently, this difference in stored elastic energy has to be interpreted as increased dissipation by plastic

deformation of the ZrCu interlayers. Following change has been made in Sec. 3, Discussion, pages 22-23, lines 623-646:

Finally, the elastic strain energy stored in the NDP, SL and ML films is considered in Fig. 7c. Since the elastic strain energy is inversely proportional to the Young's modulus E (nc diamond:

E~460 GPa[60], ZrN: E~200 GPa), the elastic strain energy in the NDP is ~2.3 times lower than in the ZrN (Fig. 7c). Given, that the elastic energy can be also expressed as $W = \int P dx$, where

P is the load and x is the displacement, similar strain energy densities can be estimated from the load-displacement data presented in Fig. 2d under the assumption of linear elasticity. That is actually the case for the NDP in contact with both the SL and ML films (*cf.* Fig. 7c). The comparably lower stress magnitudes in the NDP in contact with the ML film (Figs. 4-6) are made up by the comparably higher contact area again as can be seen from Fig. 3, cross-validating the experimental observations drawn from the thin films. On the contrary, a lower stored strain energy is present in the ML film after loading beyond 0.2 N. In total, the strain energy in the ML film at 2.0 N is only ~69% of the strain energy stored in the SL. Since plastic deformation is highly limited in the nanoceramic ZrN in both films and only possible in very small volumes at the highest multi-axial load, this strain energy difference is safely interpreted as the dissipation of strain energy through plastic deformation of the ZrCu interlayer. Consequently, the ~30% of dissipated plastic work within the ZrCu (Fig. 7c), can be regarded as a toughening mechanism, reducing the overall stress buildup and thereby enhancing the failure tolerance.

9. Symmetry-Based Error Evaluation

The inherent symmetry of the single-asperity contact permits an internal consistency check. Compare shear-stress fields on opposite sides of the interface to quantify experimental uncertainty and validate the analytical approach.

We agree with Reviewer #3 that the inherent symmetry of the single-asperity contact permits an internal consistency check. In addition to the shear stresses being point symmetric across the contact, the normal stresses introduced during loading are symmetric across the contact plane. This symmetry is found in the experimental data, the analytical approach and the FE model and is already presented in the original and revised manuscript in Fig. 5g and 5h for the contact of the NDP and the SL and ML films, respectively. Regarding the experimental uncertainty, X-ray nanodiffraction has been proven to be scientifically valid and viable several times before, both in contact mechanical situations and fracture mechanical experiments (see Refs. 45-48, 88, 91), https://doi.org/10.1038/srep22670, https://doi.org/10.1016/j.matdes.2020.108478, https://doi.org/10.1016/j.actamat.2020.05.056, https://doi.org/10.1002/adem.202100130, https://doi.org/10.1016/j.matdes.2020.109365, http://dx.doi.org/10.1038/s43246-025-00752-z). In all these studies, the experimental data have been found to be consistently in excellent agreement with theoretical considerations, as well as analytical and numerical models.

However, to adopt the comment of Reviewer #3, following change has been made to the manuscript on page 12, lines 327-329:

In agreement with the inherent symmetry of the single-asperity contact, the shear stress magnitudes are point symmetric across the contact center[65].

Furthermore, to prove here for the exclusive benefit of Reviewer #3 that the inherent consistency is given, we analysed the shear stress magnitudes along virtual sections at a 30 deg angle of the contact point in both the NDP and the SL and ML thin films, as shown in the schematic of Fig. R2a. The results of this intersection are presented in Fig. R2b-e. As can be seen from Fig. R2b and R2c, the shear stresses of the NDP in contact with the SL and ML films are point symmetric across the contact and inversely axisymmetric across both the horizontal and vertical symmetry planes. Furthermore, the inverse symmetry across the vertical axis was evaluated by summing the positive and negative shear stress left and right of the contact center in both NDP and the SL and ML films. After summation, the residual shear is presented for the NDP in contact with the SL and ML films in Figs. R2d and R2e, respectively. While there is some variation of the residual shear stress itself in the experimental data, most probably due to the finite X-ray gauge volume, finite residual misalignment of the experiment and the exact choice of the nominal contact point, the results presented in Figs. R2d and R2e show ~0 residual shear for the NDP in contact with SL and ML films, respectively. Thus, the validity of the experimental X-ray nanodiffraction approach is confirmed once more, despite already extensive literature. Please note also that all values already have associated uncertainties, explicitly included in Fig. R2, as well as Fig. 5-7 in the manuscript.

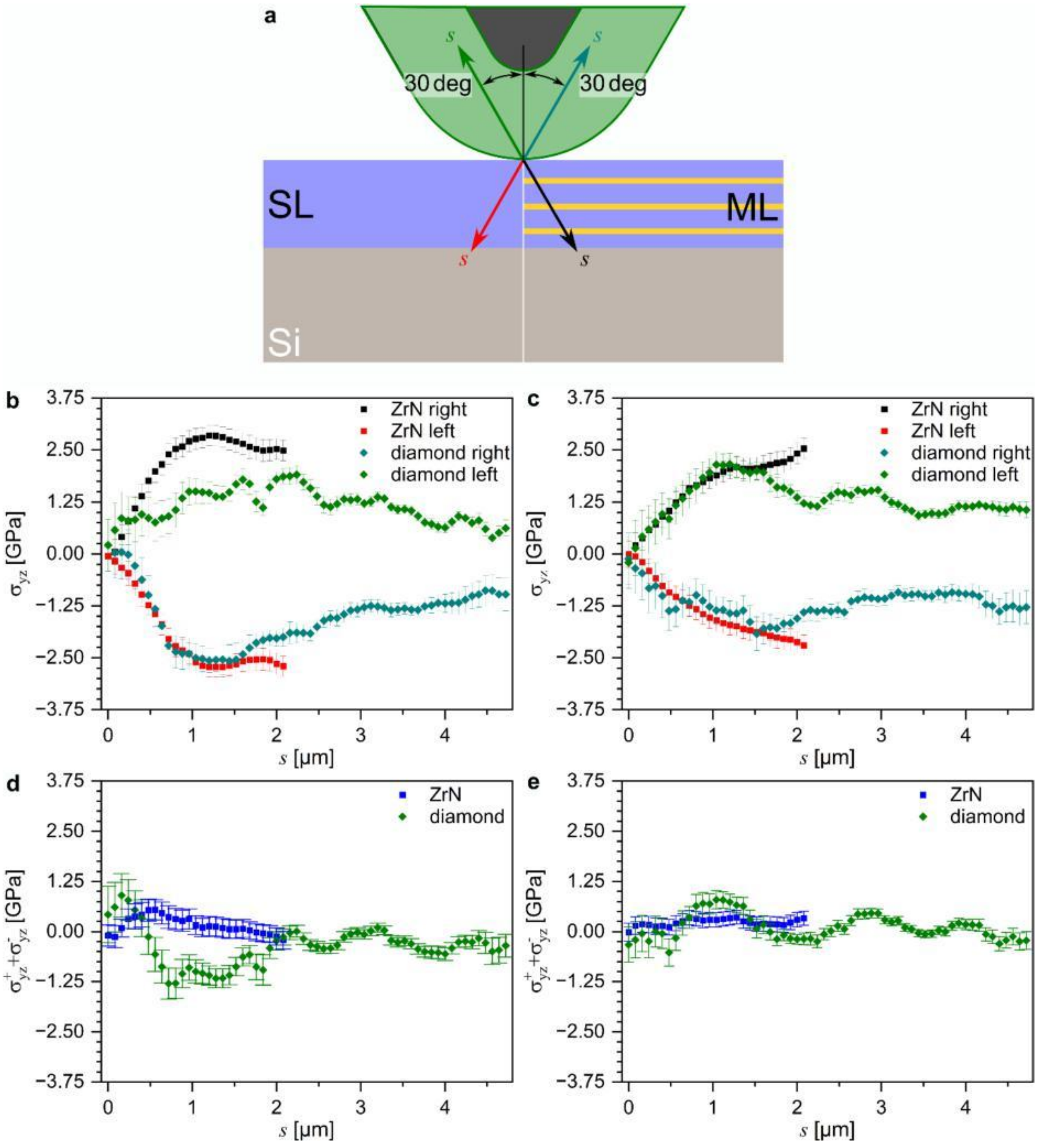

Fig. R2: Symmetry evaluation of the experimental shear stress magnitudes. In (a), a schematic is presented to highlight the line intersections *s* tilted 30 deg from the symmetry plane of the single-asperity contact along which the shear stress was evaluated. In (b) and (c), the shear stresses extracted along these intersections are presented for the NDP in contact with the SL and 1VIL films, respectively, proving the point symmetry of the shear stresses in the single-asperity contact. Additionally, in (d) and (e), the symmetry along the vertical axis of contact is shown for the NDP in contact with the SL and the 1VIL thin films, respectively. This symmetry is proven by summing the negative and positive shear magnitudes evaluated left and right of the contact in the NDP, SL and 1VIL films.

10. Finite-Element Implementation Details

- Specify the FE package employed, mesh density, convergence criteria, and boundary conditions. Detail the constitutive models (elastic, plastic, hardening rules) and parameter values (e.g., yield stress, modulus) for both ZrN and ZrCu layers, as well as for the

nanocrystalline-diamond indenter. Some elastic parameters are in the supplementary materials.

In agreement with Reviewer #3, we have updated the experimental description of the FE model in the manuscript and the supplementary material.

The following changes have been made to methods section the manuscript on page 28, lines 809-822:

The contact problem given by the NDP and the SL thin film was be interpreted by the elastic solution of the cylindrical contact given by Hertz[1,57] using the exact solution for the 2D stress fields given by M'Ewen[56]. This approach is summarized in the Suppl. Note 12. The contact problem was further simulated using the finite element (FE) method for the ML film in contact with the NDP using the commercial software package Abaqus 2023 (Dassault Systèmes Simulia, Vélizy-Villacoublay, France). Unlike conventional process models, the simulation was designed to accommodate the eigenstrains[58,59,93] inherited from the deposition of the SL and ML thin film layers. This involved defining the eigenstrain field assumed to vary only along the surface normal (z-axis) expressed as a high-order polynomial function as outlined in Suppl. Note 13. The NDP was simulated without considering residual stress in agreement with the experimental data (Suppl. Notes 3 and 4).

The indentation process was simulated by FE modelling of the contact between the indenter and the multilayer. All details of the simulation are summarized in Suppl. Note 13, while elastic constants are given in Table S1.

Furthermore, following changes have been made to Supplementary Note 13 detailing the FE model in the supplementary on pages 50-51:

The indentation process was simulated by FE modelling of the contact between the indenter and the multilayer. The coated layers (ZrN, CuZr, and the Si substrate) and the indenter (sc and nc diamond) were meshed using 3D, 8-node linear solid continuum elements (C3D8 and C3D8R) to accurately capture the stress tensor during deformation while satisfying computational efficiency. To achieve this, the indenter was represented as a cylindrical body with a radius of 5.8 µm, consisting of an inner layer of sc diamond with a radius of 2 µm and an outer layer formed from nc diamond with a thickness 3.8 µm corresponding to the physical dimensions of the knife edge. The domain encompassing the ZrCu-ZrN thin film layers and the Si substrate was modelled as a single part with distinct material properties. The dimensions of the ML film in the yz-plane were set at 20 by 6.06 µm. A fine mesh density with an element size of 125 nm was employed throughout the contact zone and critical deformation regions to ensure numerical accuracy and proper resolution of the stress gradients during indentation. Additionally, the cylindrical indenter tip and orthogonal body of the ML film were created with an thickness of 125 nm equal to the size of two cubic hexahedral elements. A symmetry condition defined at all surfaces of the hexagonal body except the indentation surface satisfied

modelling the continuity of the model domain. The bottom face of the Si substrate was fully constrained in the vertical displacement direction ($u_2 = 0$). The outer faces along the *y*-axis were constrained from moving in the *y*-direction ($u_1 = 0$), and the front and back faces along the *x*-axis were constrained in the *x*-direction ($u_3 = 0$) to simulate a continuous coating. A linear elastic-ideal plastic constitutive model (zero strain hardening) was utilized for the all materials, while material properties of this finite element model are given in Table S1.

Table S1. Material properties of the multilayer thin film layers

	Young's Modulus [GPa]	Poisson's Ratio [-]	Yield Strength [GPa]
ZrN[22]	380	0.26	7.0
ZrCu[26]	55	0.35	1.8
Si[27]	155.0	0.20	4.3
nc diamond[8,48]	460	0.15	22
sc diamond[49]	1120	0.07	.

Furthermore, the interaction between the indenter and the top ZrN coating surface was defined using a surface-to-surface contact formulation with finite sliding kinematics. To systematically investigate the effect of energy dissipation and shear stress transfer at the interface, the coefficient of friction (μ) was parametrically analyzed at values of 0.0 (frictionless), 0.5, and 1.0 utilizing an isotropic penalty friction formulation. The analysis was solved implicitly using the standard within the commercial Abaqus software. Nonlinear geometric effects were enabled to account for large deformations and contact updates during the deep indentation process. Standardized convergence criteria within the commercial Abaqus software were utilized for force and displacement residuals, with an initial increment size of 0.01 and a minimum allowable time increment of 10^{-8} to ensure strict numerical stability during the highly nonlinear contact phase.

- The current FE model treats the indenter as a rigid cylinder, neglecting possible plasticity in the nanocrystalline diamond coating. At a minimum, model the indenter as an elastic solid with a high yield strength to capture realistic stress transfer.

According to the comment of Reviewer #3, we adopted a new FE model, that treats the NDP as a half cylinder with a radius of 5.8 µm consisting of two layers. The inner layer is considered as linear-elastic diamond with a Young's modulus of 1120 GPa and a Poisson's ratio of 0.07, while the outer layer is modelled as linear elastic-ideal plastic nc diamond with a thickness of 3.8 µm and an elastic modulus of 460 GPa, a Poisson's ratio of 0.15 and a yield stress of 22 GPa. All elastic constants have been either adopted from literature (please also see our reply above) or were directly retrieved from experimental data within this publication. For example, the yield stress of the nc diamond was adopted from the hardness of the diamond thin film using

a Tabor factor of 2.8-3. The elastic and plastic parameters of all materials employed in the FE model are summarized in Table S1. Furthermore, in addition to the frictionless contact, friction coefficients of 0.5 and 1.0 have been employed. In agreement with literature sources (https://doi.org/10.1016/j.compscitech.2006.10.005, (https://doi.org/10.1016/j.jmps.2005.07.003, https://doi.lorg/10.1016/j.actamat.2004.01.016), we have found that the effect of interface friction on the obtained stress distributions is negligible for realistic friction coefficients. The results of the newly formed model are presented in Fig. 5 and in Suppl. Fig. S20-S22. The ollowing changes have been made to the manuscript: Page 14, lines 386-394:

Furthermore, the analytical data fit the experimental case studies very well in the NDP, as can be seen for the contact with the SL and the ML film in Figs. 5g and 5h, respectively. In particular, a_{zz} is represented accurately, even in magnitude by the analytical solution for both experiments (Figs. 5g and h), while a shift towards more tensile stress is observed in the experimental data for a_{yy} (Figs. 5g and h). Since it was already established that (i) the nc diamond was deformed only elastically during the experiments (*cf.* Suppl. Notes 3, 4 and 5, see also Fig. 6a and 6f, Fig. S17), (ii) the NDP follows the analytical considerations reasonably well and (iii) cracking occurs in the Si substrate in both experiments, the diamond was considered rigid in the model, while the deformation of the Si substrate was integrated within the FE model.

The numerically recreated a_{yy} stress component is presented in Fig. 5b. In addition to the stress distribution in the NDP, also the a_{yy} magnitudes in the sc diamond can be investigated within the FE model (Fig. 5b). A strong gradient from compressive to tensile stress is seen in the center of the numerically recreated NDP, in perfect agreement with the experimental data. Additionally, slight tensile stresses are found in the sc diamond (Fig. 5b).

Page 15, lines 413-421:

Regarding the modelled a_{zz} stress component presented in Fig. 5d, a maximum compressive stress of ~-12.5 GPa is found directly in the center at the interface between the ZrN and the NDP, which matches remarkably well to the experimental data (Fig. 4, Fig. 5d). Also, the sc diamond is under compressive stress, with magnitudes exceeding −7.5 GPa throughout the investigated volume (Fig. 5d). Slight deviations between the experiment and the FE model can be attributed to the boundary conditions of the modelled bilayer indenter tip. Furthermore, the outline of the stress profile fits perfectly with the experiment. Tensile stresses along the z-direction are only found in Si at the edges of the modelled area, which can be attributed to with the boundary conditions of the FE model.

Page 15, lines 426-433:

Finally, virtual vertical sections of experimental and numerical stress data are shown in Fig. 5h. Here again, the numerical data show excellent agreement with the experimental case studies,

particularly in the NDP region in contact with the ML film. (Fig. 5h). In particular, the transition from compressive to tensile stress observed for σ_{yy} is represented accurately, while slight shifts towards more compressive and tensile stresses are found for σ_{zz} in the NDP and ZrN at larger distance from the contact, respectively. These slight differences may be attributed to the boundary conditions and fracture in the Si substrate for the NDP and the ZrN, respectively.

Additionally, the following changes have been made to Suppl. Note 7, pages 36-39 of the supplementary material:

The finite element simulation of the indentation process facilitated the investigation of multi-axial stresses in the ML thin film layers subjected to indentation, since the more complex case with elastic-plastic ZrCu interlayers could not be covered by the simplified analytic solution. The contour plots in Fig. S20, S21 and S22 illustrate the 2D distributions of σ_{yy}, σ_{zz} and σ_{yz} components of applied and residual stresses at maximum indentation load (2.0 N) and after unloading (post indentation) for frictionless contact and friction coefficients of $\mu = 0.5$ and 1.0, respectively. They demonstrate good qualitative and quantitative agreement with experimental results given in Figs. 4 and 5. Additionally, simulation results reveal stress hotspots during loading and post-loading conditions, which are not found in the experimental data, possibly because these features could be too small in comparison to the X-ray gauge volume (*cf.* Methods, Suppl. Note 13).

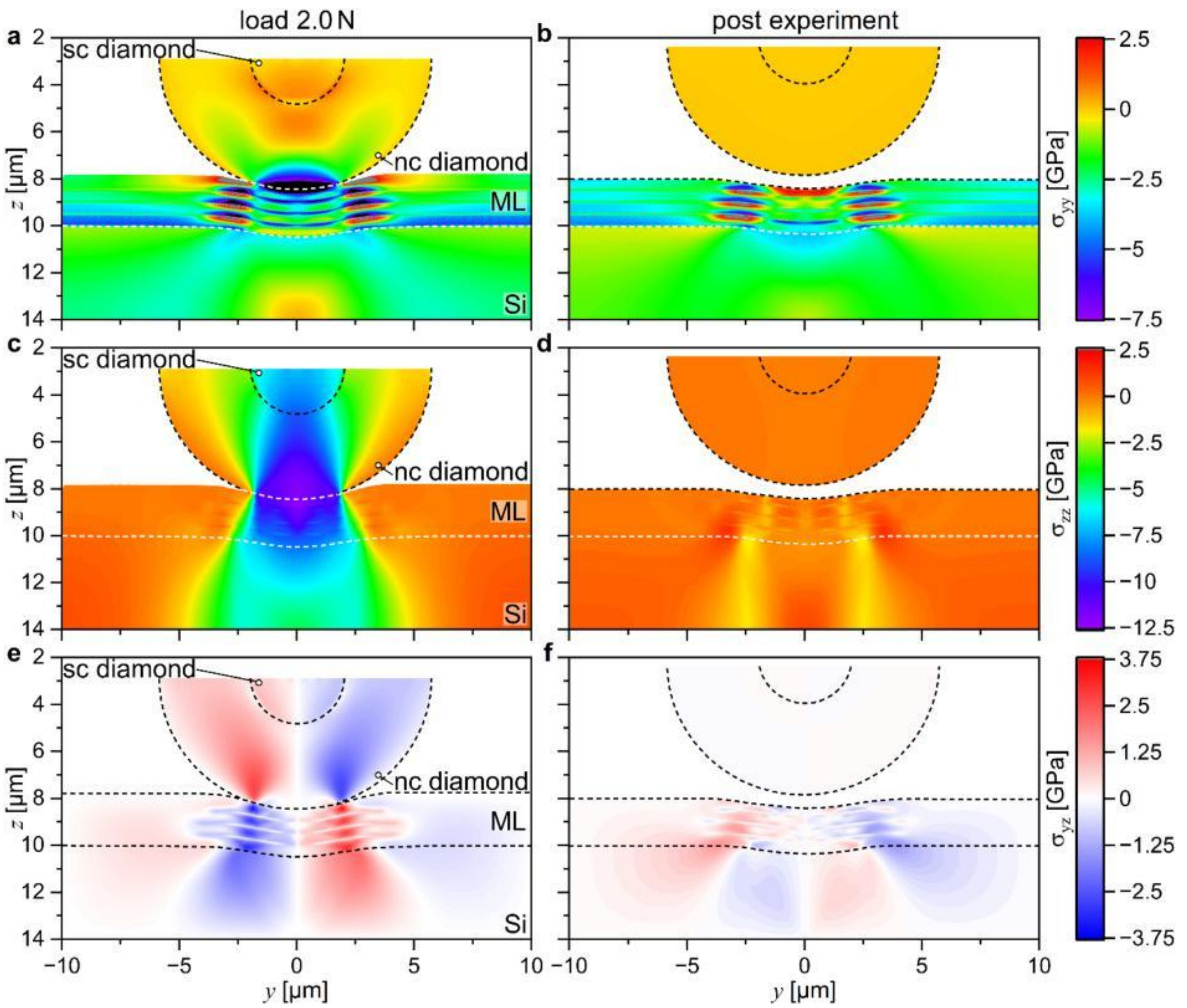

Figure S20. Distribution of σ_{yy}, σ_{zz} and σ_{yz} stress components of applied and residual stresses obtained from frictionless contact at 2.0 N load and after the experiment are presented in (a-c) and (d-f), respectively.

First of all, when comparing the results obtained for the different friction coefficients (Fig. S20-S22), we see that the increasing tangential traction induced by the friction at the interface between nc diamond and ZrN noticeably affects the resulting stress distributions only at the highest friction coefficient of $\mu = 1.0$. But, even at the highest friction coefficients, the stress distributions resulting from the tangential tractions remain much smaller than the normal contact stresses. This is in agreement with existing literature dealing with nanoindentation stress distributions, which found that the influence of the friction force is negligible for indentation considering reasonable friction coefficients below 0.3[34–36]. Additionally, comparable literature data suggests that the friction coefficient between two ceramics is typically in the order of 0.1 or lower[37,38]. Therefore, the following presentation of the results extracted from the FE model will be limited to the numerical data obtained under frictionless contact.

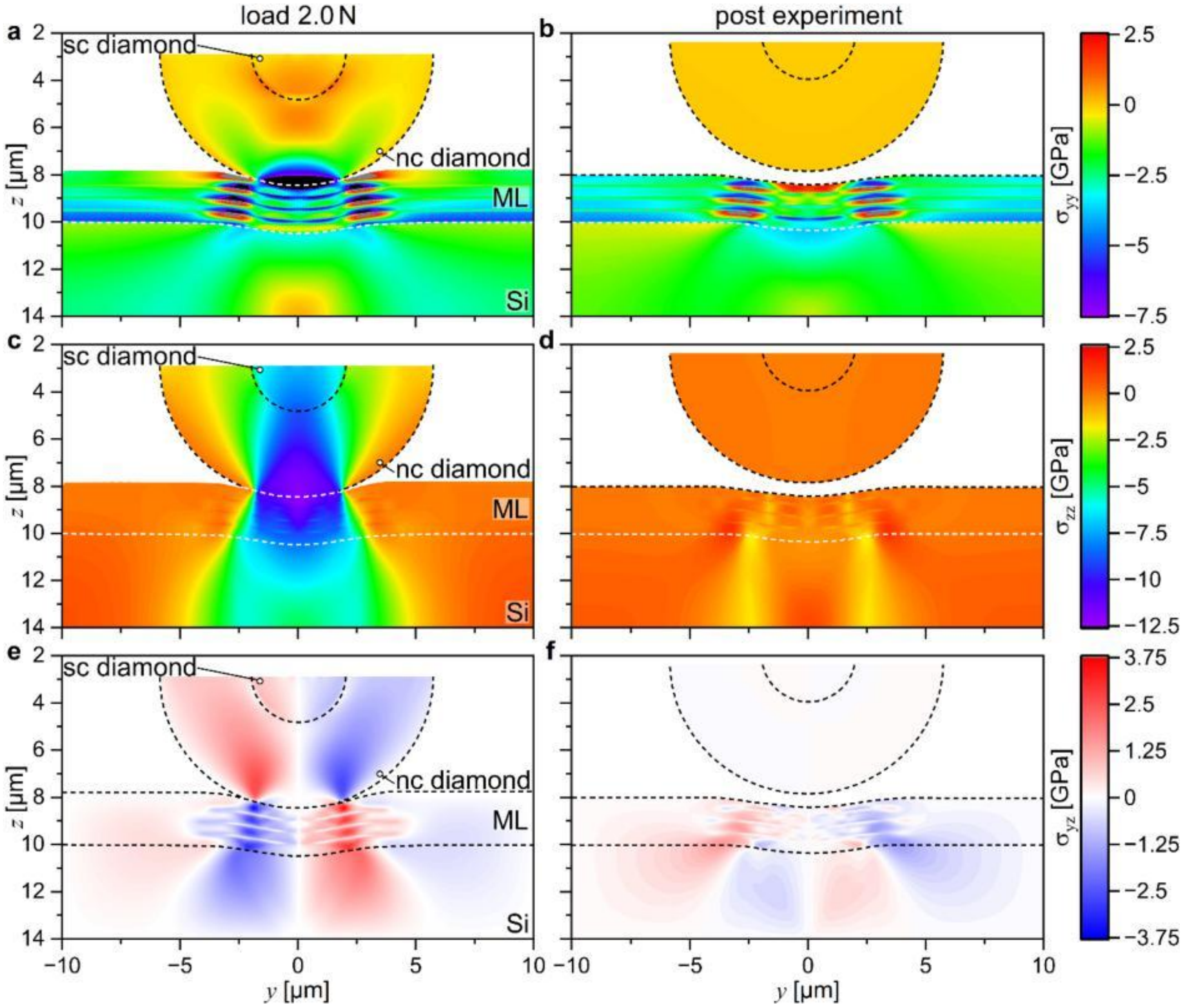

Figure S21. Distribution of σ_{yy}, σ_{zz} and σ_{yz} stress components of applied and residual stresses obtained from contact with a friction coefficient of μ = 0.5 at 2.0 N load and after the experiment are presented in (a-c) and (d-f), respectively.

In case of the ML film, the results presented in Fig. S20 indicate that the distribution of indentation and residual stresses reflect the combined effects of contact compression and the overall three-point bending of a double-clamped cantilever[20] govern the response of the sample. Of course, the bending-like response is limited by the continuous support given by the Si substrate and can be only used for comparison. Each layer of the ZrN multilayer exhibits bending character, since the presence of ZrCu interlayers represents a weakened transmission of tractions between these layers.

In case of the diamond tip (Fig. S20), an excellent agreement of the numerically obtained stress distributions with the experimental data (Fig. 4) and the analytical model (Fig. S19) is observed. Furthermore, the influence of the sc diamond substrate is highlighted, as the FE model is able to recreate the tensile σ_{yy} magnitudes observed close to the nc/sc diamond interface.

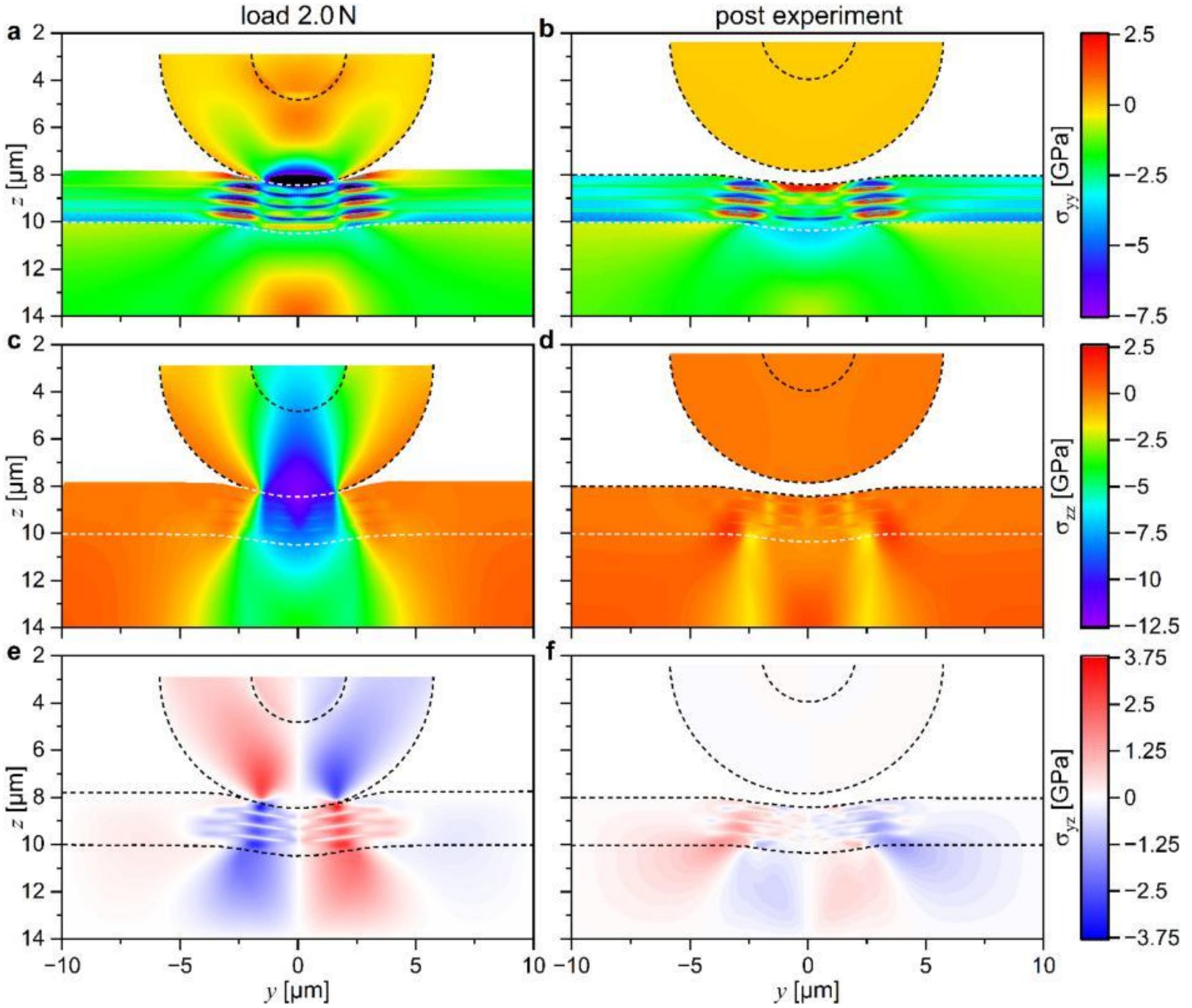

Figure S22. Distribution of σ_{yy}, σ_{zz} and σ_{yz} stress components of applied and residual stresses obtained from contact with a friction coefficient of μ = 1.0 at 2.0 N load and after the experiment are presented in (a-c) and (d-f), respectively.

Furthermore, the experimental study did not allow the determination of stresses in the single crystal Si substrate and the sc diamond. Here, the simulation results reveal the stress distribution in the Si substrate layer and show that the σ_{yy}-component of stress attain high magnitudes in compression during knife edge indentation loading and exhibit a similar, but reduced distribution in the post-loading state, with an additional stress concentration noted in the sublayer of ZrN lying closest to the Si substrate. Additionally, in the sc diamond minor tensile

σ_{yy} stress magnitudes are observed during loading, while σ_{zz} is ~-5 to -7.5 GPa. The high magnitude of σ_{zz}-component compressive stresses during loading relaxes after unloading, but additional eigenstrains formed during indentation cause the formation of tensile residual stresses on both sides of the indentation. Finally, both the nc. and sc diamond appear stress free after unloading, confirming the absence of plasticity in the NDP during indentation.

11. Experimental Configuration and Replicability
The custom indentation setup is described insufficiently for replication. Include schematics, dimensional drawings, load-cell specifications, and alignment procedures.
According to the suggestion of Reviewer #3, a more detailed description of the experimental setup is now given in the revised manuscript and supplementary material. However, technical drawings of the indenter setup are considered excessive information by the authors and will not be added to the revision. Following changes were made to Sec. 4 on page 26, lines 755-766: The CSnanoXRD experiments were performed at the nanofocus extension of the beamline ID13 of the European Synchrotron Radiation Facility (ESRF) in Grenoble, France[86,87]. A pair of multi-layer Laue lenses working with a vertical and horizontal focus size of 75 nm and a focal depth of 50 µm, were used along with the dedicated indentation setup[45] available at the beamline. A full description of the indentation setup is provided in the Suppl. Note 11. An X-ray photon energy of 13.0 keV equivalent to a wavelength of 95.3725 pm was employed.

Prior to the experiment, the interface between the sc and nc diamonds, as well as the interface between the thin films and the Si substrates were individually aligned parallel to the incident X-ray beam by performing a set of absorption line scans along the y and z-axes at various sample orientations φ and ξ using a passivated, implanted, planar silicon diode to measure transmitted X-ray intensities. The optimal sample position and tilt were determined by maximizing the absorption contrast between the individual layers and is detailed in Suppl. Note 11.

Additionally, following text was added to Suppl. Note 11 on page 45 detailing the experimental setup, the positioning stages, load cell and displacement sensor:

The custom indentation setup positioned at the nanofocus extension of the ID13 beamline at the ESRF is presented in Fig. S26. The indenter is equipped with two SLC1720 linear positioning axis (Smaract GmbH, Oldenburg Germany) as well as a CGO-60.5 goniometer stage (Smaract GmbH, Oldenburg Germany). This combination of positioners allows an independent sample alignment and positioning along the x, y, and φ axes. N-310 NEXACT OEM Miniature Linear Motor (PI (Physik Instrumente) L.P., Shrewsbury, Massachusetts, USA) was used for the vertical (z) movement of the indenter tip, which was monitored by a RLB Miniature Incremental Magnetic Encoder Module (RLS d.o.o., Ljubljana, Slovenia) with a displacement resolution of 250 nm. The force was monitored using a KD24 load cell (ME-Systeme GmbH, Germany with a nominal range of 2 N and an accuracy class of 0.1%).

The indenter tip was aligned parallel to the incident X-ray beam performing series of absorption scans along the y and z axes at various positions of the φ and ξ rotational axes of the hexapod positioner, respectively (Fig. S26). The optimal orientation of the tip was identified by maximizing the absorption contrast between the nc diamond and air as well as between the nc and sc diamond. The sample was oriented by additional absorption scan along the z-axis at various tilts along the inner ξ rotational axis of the indenter setup (Fig. S26). Again, the optimal

orientation was determined by maximizing the absorption contrast between the thin film and the air.

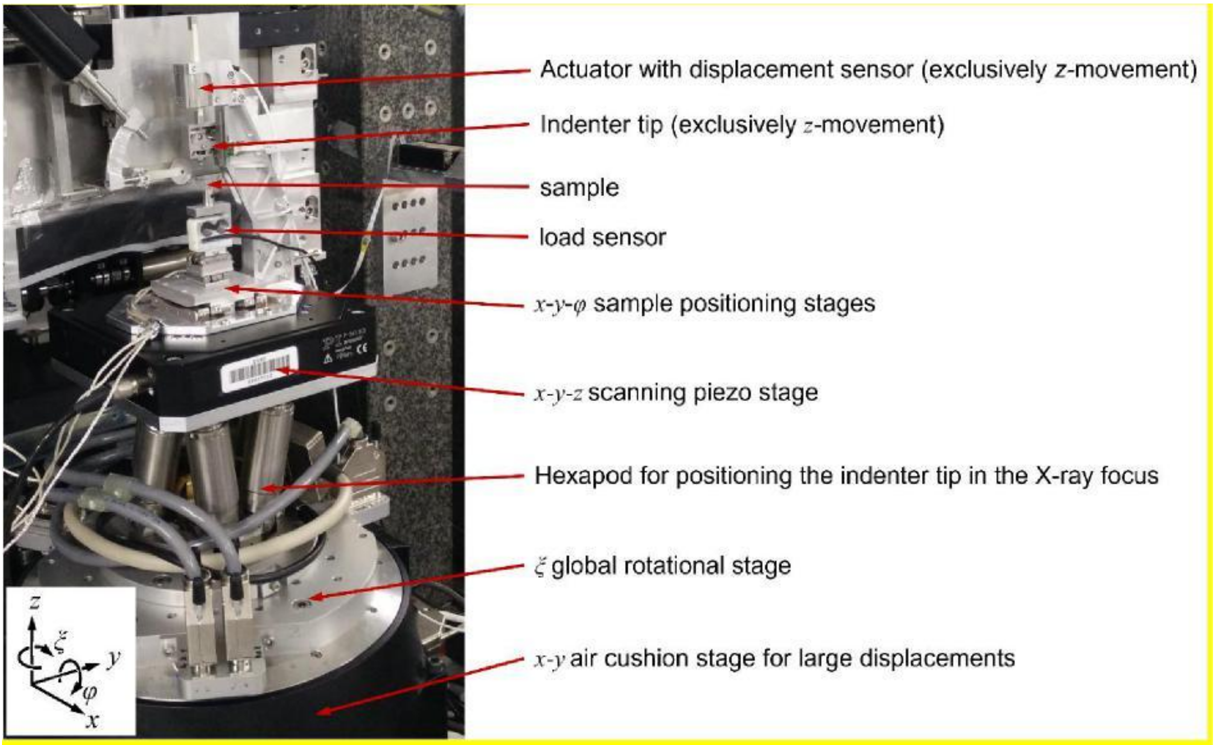

12. Figure Captions

The caption for Fig. 6 is overly brief and does not convey the experimental context or the key observations.

According to the suggestion of Reviewer #3, we have extended the figure caption of Figure 6 as follows:

Figure 6. A detailed stress comparison of the SLand ML in contact with the NDP. A schematic of the

individual analyses presented is shown in (a), representing the line intersections of the 2D stress fields presented in Fig. 3 in the center of the contact between the SL and ML films and the NDP. In (a), the σ_{yy} line profiles depicting the residual stress in the SL and ML as well as the NDP before the experiments. Note that the NDP is nearly stress-free before the experiment (a). The σ_{yy} line profiles across the indenter-sample interface are shown for the SL and the ML in contact with the NDP for the individual load steps of 0.2, 0.5, 1.0 and 2.0 N in (b), (c), (d) and (e), respectively, while the residual stress accumulation in the SL and ML films as well as the NDP after the experiment is presented in (f). The σ_{yy} and σ_{zz} stress values accumulated at the contact and the ZrN/Si interface are shown for the SL and the ML in (g) and (h), respectively, as shown in the schematic in (a).

13. Probe Characterization

Mechanical properties of the nanocrystalline-diamond coating—elastic modulus, hardness, and any residual stresses—are not quantified, yet they critically influence stress transfer across the interface.

We agree with Reviewer #3 that the hardness of the nanocrystalline diamond coating was not characterized in the original manuscript. To adopt this, nanoindentation hardness was measured on nc diamond deposited on sc Si using exactly the same parameters in the same deposition

system. The Young's modulus of nc diamond deposited using exactly the same parameters was already measured in Ref. 51 (https://doi.org/10.1116/6.0003235), which was also added to the manuscript ensuring proper characterization of the NDP. The following changes have been made to the manuscript:

Sec. 2.1, on page 5, lines 163-166:

Additionally, nanoindentation testing was carried out on the nc diamond deposited on sc Si to assess the hardness of the NDP, which was evaluated as 61.6±6.1 GPa, while the Young's modulus of 460±53 GPa of the same thin film was already measured in a prior study[60].

Sec. 4 on page 24, lines698-702:

The nanocrystalline (nc) diamond thin film was deposited on a wedge-shaped single crystalline (sc) diamond indenter tip with a tip radius of 2 µm, a length of 85 µm and wedge opening and side opening angles of 60 and 90 deg, respectively (Synton MDP, Nidau Switzerland) by modified HF-CVD[79] and additionally onto sc Si wafer with a diameter of 30 mm and a thickness of 530 µm.

Sec. 4 on page 26, lines 744-749:

Nanoindentation tests on the nanocrystalline diamond were performed using a G200 nanoindenter (KLA Corporation, Milpitas, California, USA) equipped with a continuous stiffness measurement (CSM) unit and different indenter tip geometries (Synton-MDP, Nidau, Switzerland). Diamond Berkovich indentation experiments were carried out in strain-rate controlled mode (0.05 s^{-1}) to a maximum indentation depth of 500 nm. Hardness was evaluated according to the Oliver–Pharr method[37] and averaged over the depth range of 420–480 nm.

However, the residual stress in the tip is characterized before and after both experiments and was already presented both in Fig. 6a and 6f, respectively, as well as in the supplementary material (Supplementary Notes 3 and 4). The evaluated stress data before the experiment show that the tip is nearly stress-free. This is in agreement with Ref. 51 of the revised manuscript, where nc diamond was produced using exactly the same deposition parameters and characterized using cross-sectional X-ray nanodiffraction and microcantilever bending tests. Mild tensile residual in the order of 0.25-0.5 GPa stress was evaluated from the same nc diamond deposited on Si using exactly the same deposition parameters. The intrinsic stress component estimated in Ref. 51 to ~0.6-0.85 GPa. Since the diamond tip as a substrate has a potentially smaller coefficient of thermal expansion compared to Si, ~0 residual stress at room temperature is expected in case of the diamond tip. Therefore, since the residual stress is negligible in the NDP before the experiments, we do not expect a critical influence on the stress transfer during the experiment. To adopt to the comment of Reviewer #3 following adjustment was made to Supplementary Note 3, including the origins and magnitude of residual stress in the NDP on page 9 of the supplementary material:

Before contact, the nc diamond forming the nanomechanical probe was essentially free of residual stress, as can be seen from the 2D contour plots of the σ_{yy} and σ_{zz} stress components presented in Fig. S6c and d, respectively. Residual stress of nc diamond deposited on Si with

the exactly same deposition conditions was characterized by cross-sectional X-ray nanodiffraction and quantified as ~0.5 GPa[8]. Taking into account the thermal expansion coefficients of Si[9] and nc diamond[10], the thermal and intrinsic stress components were estimated as -0.1 to -0.35 GPa and +0.6-0.85 GPa, respectively. Taking into account the lower thermal expansion coefficient of single crystalline diamond[11–14], a thermal stress component of ~-0.9 GPa can be estimated for nc diamond deposited on sc diamond. Consequently, summing up the thermal and intrinsic stress components for the nc diamond grown on sc diamond, the experimentally observed absence of residual stress (Fig. S6c and d) is in agreement with the growth conditions present in the literature.

15. Citation Density
The introduction and discussion contain an extensive list of peripheral references that dilute the focus on the central contribution. Reduce citation overload by retaining only the most directly relevant studies.
According to the suggestion of Reviewer #3, we have removed some of the citations in the manuscript, helping the potential reader to focus on the key findings of the study. Particularly, we have removed citations regarding biomaterials in the introduction (please see also our reply to query 2 of Reviewer #3).

16. Generalization to Biological Composites
Drawing parallels between the present findings and biological composites (nacre, bone) is conceptually appealing but speculative. Temper the discussion of such analogies, and if possible, provide quantitative comparisons to substantiate the claim.
We thank Reviewer #3 for his critical insight. According to this suggestion, we have added a quantitative comparison of our results to the work of Gim *et al.* (Ref. 77, http://dx.doi.org/10.1038/s41467-019-12743-z). The following changes have been made to the manuscript on page 23, lines 649-658:
Exemplarily, Gim *et al.*[77] proved how the organic layers in nacreous aragonite lead to a strain redistribution, thus preventing catastrophic failure of the macroscale architecture. During loading with a nanoindenter, prismatic calcite and monolithic aragonite experienced catastrophic failure by sub-surface fracture at nominal stresses of 1.4 GPa and showed low energy dissipation. On the other hand, failure was mitigated in the nacreous aragonite by fracture within the individual platelets held together by the flexible biopolymer[77] at ~80 % nominal stress, while energy dissipation was increased by a factor of 2.6 compared to monolithic aragonite. This is consistent with the behavior observed in Figs. 4 and 5, where the maximum σ_{yy} and $\sigma_{\text{von Mises}}$ levels in the ML film are reduced by ~ 10% compared to the SL film (Figs. 4-6), while the overall stability of the architecture is increased.

Editorial comments:

Titles and Captions: We notice the Figure 3,5,7, panel f, h, b is not defined in the legend. Please ensure that all figure panels are defined.

We reviewed all figure captions and the following changes have been made:

Figure 3. Microstructural changes in the SL and ML as well as in the nanocrystalline tip probe at a load of 2.0 N. In (a) and (b) the SAXSM micrographs obtained from the SL and ML are shown. The highlighted region in (a) represents a step in the Si/ZrN interface. In (c) and (d) micrographs depicting the azimuthally averaged FWHM are presented, respectively. The dotted black line in (c) and (d) represents a FWHM of ~0.65 deg. The schematic experimental setup (e) is shown alongside the progressive evolution of the FWHM interaction *width* and *depth* during incremental indenter loading presented in (f).

Figure 5. Comparison of the experimental data with analytically-derived and FE-modelled stress distributions. The σ_{yy}, σ_{zz} and σ_{yz} stress distributions derived by the linear-elastic analytical solution given by M'Ewen[64] are shown in comparison with the experimental data from the NDP in contact with the SL film in (a), (c) and (e), respectively, while the modelled σ_{yy}, σ_{zz} and σ_{yz} stress distributions are presented against the experimental data of the ML in (d), (e) and (f), respectively. Additionally, virtual sections along the symmetry plane were taken from the SL and ML in contact with the NDP and compared with the analytical and numerical data in (g) and (h), respectively. In (g), $\sigma_{r,ZrN}$ represents the average residual stress present in the SL before the experiment (Suppl. Note 3).

Figure 7. Geometrical response data and stored elastic energies within the SL and ML in contact with the NDP. In (a), the averaged intensities of the ZrN 111 DS ring retrieved from the SL and ML are presented together with a schematic of the NDP. The individual positions of curvature measurements for both films are also shown in (a), while the curvatures retrieved from the contact and the interface in the SL and ML films are presented in (b). The stored elastic energy per line element is shown for the SL and the ML in (c), the difference represents the energy release of the ML film. The scale bar in (a) equals 2 µm.

Author Information: Please ensure that each affiliation contains the institution name, city, and country.

We have reviewed all affiliations and added the full postal addresses to all affiliations. We also found that the cities and countries of all co-authors were in good order.

Titles and Captions: Please ensure that all Figures/Schemes/Tables are cited, and that citations are in numerical order within the main text. [EA can specify if a certain item is out of order] We have reviewed the titles, captions and citations of all figures and put them in numerical order within the text.

Code Availability. Guidance: For all studies using custom code or scripts in the generation or analysis of datasets, a separate statement must be included after the Methods section, under the heading 'Code availability', indicating whether and how the code can be accessed, including any restrictions to access. This section should also include information on the versions of any software used, if relevant, and any specific variables or parameters used to generate or analyse the current datasets. For manuscripts that contain custom code or report new software - We strongly encourage authors to deposit all code associated with the paper in a repository where they can be freely and enduringly accessed. Obtain a DOI and add to the reference list. Cite the reference in the text alongside the URL.

A code availability statement was added to the manuscript at page 29, lines 866-868:

Code availability statement

The codes that support the findings of this study are available from the corresponding authors upon reasonable request.

The authors thank again the three Reviewers for their critical insight and answered all of their questions. The submission contains *"the use of NDP, which allows tracking of stresses in the indenter alongside the thin film "* and *"the metallic glass ZrCu layers in the ML function as stress dissipators and reduce stress concentration, thereby preventing crack propagation across the ZrN-Si interface "* (Reviewer #1). Additionally, it *"presents an exceptionally novel and technically advanced study that combines state-of-the-art in situ synchrotron X-ray nanodiffraction with a custom nanoindentation approach to directly probe stress evolution at single-asperity contacts"* (Reviewer #2) as well as it *"discusses the creation of a nanomechanical diamond probe and its integration with in-situ synchrotron X-ray nanodiffraction to directly map multiaxial stress fields on both the indenter and thin-film sides of a single-asperity contact"* (Reviewer #3). Furthermore, *"The combination of modern characterization tools with carefully designed multilayer architectures results in a dataset that is both rich and scientifically valuable "* (Reviewer #2). Again, we consider this work of interest to the general readership of *Communications Materials* and hope for your positive response.

Answer to the Reviewer and List of Changes in the Revised Manuscript

This is the second revision of the manuscript entitled "How Interfaces Limit Nanoscale Stress Concentrations and Prevent Catastrophic Failure in Single-Asperity Contacts". The authors are very grateful to the three Reviewers for their valuable comments, suggestions, and critical remarks. They are highly appreciated because they give us an independent view on our work, point out errors and pitfalls, which we have not realized, and help us to formulate the manuscript in a more understandable way leading to an overall improved version of our manuscript. All Reviewers' comments and suggestions were carefully considered and adopted.

In the response to the reviewers below, we explain how we handled the Reviewers' comments point by point. For a better read- and traceability, our answers and explanations directly addressed to the reviewers are written in turquoise; the text copied in the manuscript – if still taken from the as-submitted version of the manuscript are in black; changes in the second revision of the manuscript are highlighted in green, while the changes following the first revision remain highlighted in yellow. Removed text from the first revision is omitted in the manuscript. Text removed during the second revision is crossed in the response and the manuscript.

Reviewer #1 (Remarks to the Author):

The authors have adequately addressed my questions from the first review.

The authors thank Reviewer #1 for the positive judgement of our work and are pleased that the revisions were sufficient.

Reviewer #3 (Remarks to the Author):

The authors are to be commended for engaging in constructive and insightful scientific discussions in response to the issues raised during the review process. However, given that all three reviewers identified concerns regarding the manuscript structure, and that this structure has remained largely unchanged, it is recommended to streamline the manuscript. Specifically, the focus should be narrowed to the central theme—namely, the indentation of multilayers—while peripheral content (such as beam-bending analyses, incomplete or partial numerical models, and comparisons to biological materials) should be removed. Such revisions would allow the manuscript to concentrate on its core contributions, while any omitted material could be more appropriately referenced through a thesis or related publications. The space gained through this reduction could then be used to transfer relevant content from the supplementary materials into the main text.

According to the suggestion of Reviewer #3 we have streamlined the content of the manuscript in Sec. 2.1, page 5. Specifically, we have retained the results from the cantilever bending tests to Suppl. Note 2, while only the Vickers and spherical nanoindentation data remain in Fig. 2. Additionally, following changes have been made on page 6, lines 187-199:

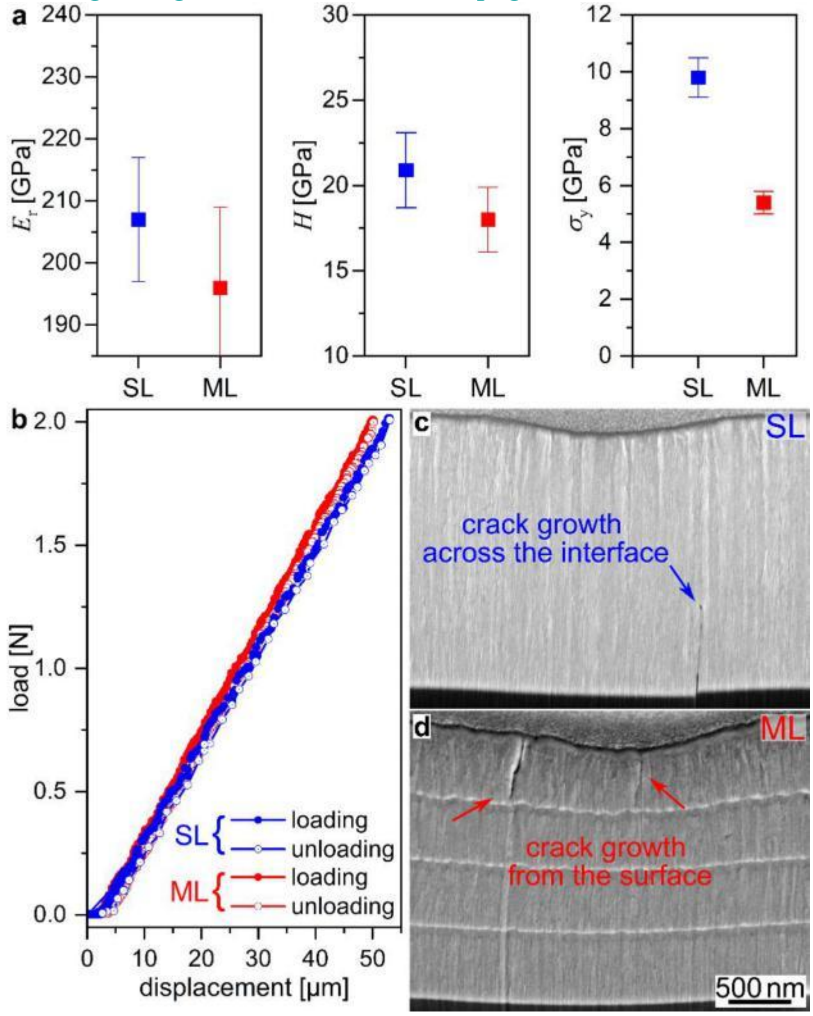

Figure 2. Results from micromechanical tests obtained from the monolithic (SL) and multilayered (ML) films. In (a), micromechanical properties of the films are presented, from left to right, the indentation modulus E_r, the nanoindentation hardness H, the elastic modulus E, fracture stress σ_F and fracture toughness K_{IC} obtained from the micromechanical bending tests, as well as the composite yield stress σ_y obtained by spherical nanoindentation. The load-displacement curves recorded from the *in situ* indentation experiments coupled with X-ray nanodiffraction (*cf.* Fig. 1) are presented in (b). In (c) and

(d) fracture surfaces of unnotched microcantilevers are displayed, while in (c) and (d) the FIB-polished *post mortem* cross-sections from the center of the imprint are shown for SL and ML films, respectively. The scale bar in (d) is applicable to (c) and (d) while the scale bar in (f) is applicable to (e) and (f).

Apparent deviations from a rectangular cross section in (c) and (d) are due to the necessity to image these surfaces at an angle, thus they are insignificant.

Additionally, the authors removed any mention of biological materials from the manuscript in the abstract and the discussion section.

Abstract

Contact between materials occurs through microscale asperities, where stress evolves under highly localized, multiaxial deformation. Despite the significant importance to numerous scientific fields, to date, direct experimental quantification of these stress fields across the materials in contact remained elusive. Here we combine *in situ* scanning synchrotron X-ray nanodiffraction with 80 nm spatial resolution and a custom indentation setup to resolve nanoscale stress fields developing at the contact between a diamond indenter and both single-layer ZrN and multilayered ZrN–ZrCu thin films. We introduce a nanomechanical probe comprising a single-crystalline diamond wedge coated with nanocrystalline diamond to simultaneously probe stress accumulation within the indenter. X-ray scattering and scanning electron microscopy, micromechanical testing, analytical and elastic-plastic finite element modeling corroborate the nanoscale stress maps obtained for both sides of the tip-surface contact region. The individual interfaces along the projected load paths diffuse stresses in both the tip and the multilayer, thereby controlling the overall mechanical response at the asperity. Consequently, the stored elastic energy is reduced by 30% in the multi-layered film in comparison with the monolithic ZrN. Finally, the measured nanoscale stress distributions elucidate the fundamental mechanisms by which artificial and biological multi-layered materials effectively dissipate energy and prevent catastrophic failure.

Sec. 3, Page 23: lines 651-660:

Exemplarily, Gim *et al.*[76] proved how the organic layers in nacreous aragonite lead to a strain redistribution, thus preventing catastrophic failure of the macroscale architecture. During loading with a nanoindenter, prismatic calcite and monolithic aragonite experienced catastrophic failure by sub surface fracture at nominal stresses of 1.4 GPa and showed low energy dissipation. On the other hand, failure was mitigated in the nacreous aragonite by fracture within the individual platelets held together by the flexible biopolymer[76] at ~80 % nominal stress, while energy dissipation was increased by a factor of 2.6 compared to monolithic aragonite. This is consistent with the behavior observed in Figs. 4 and 5, where the maximum σ_{yy} and $\sigma_{von\ Mises}$ levels in the ML film are reduced by ~ 10% compared to the SL film (Figs. 4 6), while the overall stability of the architecture is increased.

However, the analytical model recreating the contact between the SL film and the NDP is retained in the manuscript, since it aligns completely within the scope of the manuscript and was approved by Reviewers #1 and #2. The suggestion to further “narrow the manuscript to the

central theme" appears to be in contrast with the evaluations of Reviewers #1 and #2, both of whom considered the revised manuscript to be well organized and appropriate in scope.

In addition, the presentation of figures requires improvement. The font sizes appear inconsistent and disproportionate; for example, the labels "SL" and "ML" at the top of figures are significantly larger than comparable labels within the layers, despite smaller fonts being sufficiently legible (as demonstrated in Figure 5). Furthermore, Figure 5 combines differing plotting styles (panels g and h), and the use of orange text on a green background reduces readability.
According to the suggestion of Reviewer #3, all font sizes have been harmonized in all Figures.

Finally, the treatment of plasticity warrants further clarification. Figure 5 indicates that the stresses in the ZrCu layers exceed the reported yield stress (1.8 GPa) in multiple instances. This suggests that plastic deformation should occur, with adjacent layers constraining this behavior and thereby generating shear stress waves within the plastic regime. Indeed, the finite element results in Figure 5 show pronounced shear stress waves in the plastic domain, exceeding those observed in the elastic regime. However, the experimental results do not reflect this distinction, instead showing similar shear stress wave behavior in both elastic and plastic domains. This discrepancy should be addressed and explained in greater detail.
The authors thank Reviewer #3 this additional comment. Classical continuum theory of metal plasticity states that the attainment of certain measure of shear stress triggers its onset. This means that under confining hydrostatic pressure there would be no flow. Therefore, a single stress component value should not be taken as an indicator for plastic flow. We clarified that to the potential reader in Sec. 2.3 on page 15, lines 404-406:

Please note that the individual stress components may exceed the yield stress of ~1.8 GPa of ZrCu[50,59], since the hydrostatic component of the stress tensor limits plastic flow.

Regarding the absence of the short-range stress concentrations in the experimental data, we now discuss the experimental limitations with respect to all stress components. Following changes were made in Sec. 2.3 on page 15, lines 407-412:

A likely explanation for this discrepancy can be found in the experimental limitations, such as the finite X-ray gauge volume, the roughness of the individual interfaces or slight misalignment against the φ and ξ rotational axes (see Fig. 1, Methods), all of which prevent the resolution of these ultra short-range stress concentrations in the experimental data. This is not only true for the σ_{yy} stress component, but for also for σ_{zz} and σ_{yz}.

Editorial comments:

- Please revise the manuscript structure to narrow the focus to the central theme. Peripheral content can be added to the supplementary material. See Reviewer's #3 suggestions

We have retained the micromechanical bending tests and removed any mentioning of biological composites. For details, revisit comment 1 of Reviewer #3.

- Please use similar format and font sizes for all figures.

We revised all figures and applied similar appropriate formatting and font sizes for all of them.

- Please add a paragraph explaining the discrepancy between computational and experimental results shown in Fig. 5.

A paragraph was added to the manuscript detailing the difference between the experimental and numerical stress results. For details, please revisit comment 3 of Reviewer #3.

- https://doi.org/10.15151/ESRF-ES-964909431 - this link in data availability invalid, please provide the correct link.

We thank you for pointing out this ambiguity. The authors provide now specific DOIs for the individual datasets generated for this study. Following changes have been made to the data availability statement:

The raw data generated in this study have been deposited in the ESRF database under accession codes https://doi.org/10.15151/esrf-dc-2439292038 and https://doi.org/10.15151/ESRF-ES-1426505856. Access can be obtained upon request. Processed research data for this work are available from the authors upon reasonable request.

The authors thank again the three Reviewers for their critical insight and answered all of their questions. The submission contains *"the use of NDP, which allows tracking of stresses in the indenter alongside the thin film "* and *"the metallic glass ZrCu layers in the ML function as stress dissipators and reduce stress concentration, thereby preventing crack propagation across the ZrN-Si interface "* (Reviewer #1). Additionally, it "*presents an exceptionally novel and technically advanced study that combines state-of-the-art in situ synchrotron X-ray nanodiffraction with a custom nanoindentation approach to directly probe stress evolution at single-asperity contacts"* (Reviewer #2) as well as it "*discusses the creation of a nanomechanical diamond probe and its integration with in-situ synchrotron X-ray nanodiffraction to directly map multiaxial stress fields on both the indenter and thin-film sides of a single-asperity contact"* (Reviewer #3). Furthermore, "*The combination of modern characterization tools with carefully designed multilayer architectures results in a dataset that is both rich and scientifically valuable "* (Reviewer #2). Again, we consider this work of interest to the general readership of *Communications Materials* and hope for your positive response.
